# P2X_7_R influences tau aggregate burden in human tauopathies and shows distinct signalling in microglia and astrocytes

**DOI:** 10.1016/j.bbi.2023.09.011

**Published:** 2023-11

**Authors:** Paula Beltran-Lobo, Martina M. Hughes, Claire Troakes, Cara L. Croft, Huzefa Rupawala, Daniel Jutzi, Marc-David Ruepp, Maria Jimenez-Sanchez, Michael S. Perkinton, Michael Kassiou, Todd E. Golde, Diane P. Hanger, Alexei Verkhratsky, Beatriz G. Perez-Nievas, Wendy Noble

**Affiliations:** a King’s College London, Institute of Psychiatry, Psychology and Neuroscience, Department of Basic and Clinical Neuroscience, 5 Cutcombe Road, London SE5 9RX, UK; b London Neurodegenerative Diseases Brain Bank, Institute of Psychiatry, Psychology and Neuroscience, Kings College London, London, UK; c UK Dementia Research Institute, UCL Institute of Neurology, University College London, London, UK; d The Francis Crick Institute, London, UK; e UK Dementia Research Institute, Institute of Psychiatry, Psychology & Neuroscience, King's College London, London, UK; f Neuroscience, IMED Biotechnology Unit, AstraZeneca, Cambridge CB21 6GH, UK; g School of Chemistry, Faculty of Science, University of Sydney, Sydney, New South Wales, Australia; h Department of Pharmacology and Chemical Biology, Department of Neurology, Emory Center for Neurodegenerative Disease, Emory University, Atlanta, GA, USA; i Faculty of Biology, Medicine and Health, The University of Manchester, Manchester, UK; j Achucarro Center for Neuroscience, IKERBASQUE, 48011 Bilbao, Spain; k Department of Forensic Analytical Toxicology, School of Forensic Medicine, China Medical University, Shenyang, China; l Department of Stem Cell Biology, State Research Institute Centre for Innovative Medicine, LT-01102 Vilnius, Lithuania; m University of Exeter, Department of Clinical and Biomedical Science, Hatherly Laboratories, Prince of Wales Road, Exeter EX4 4PS, UK

**Keywords:** Alzheimer’s disease, Tauopathy, P2X_7_R, Astrocyte, Microglia, RNAScope, Human brain

## Abstract

The purinoceptor P2X_7_R is a promising therapeutic target for tauopathies, including Alzheimer’s disease (AD). Pharmacological inhibition or genetic knockdown of P2X_7_R ameliorates cognitive deficits and reduces pathological tau burden in mice that model aspects of tauopathy, including mice expressing mutant human frontotemporal dementia (FTD)-causing forms of tau. However, disagreements remain over which glial cell types express P2X_7_R and therefore the mechanism of action is unresolved. Here, we show that P2X_7_R protein levels increase in human AD post-mortem brain, in agreement with an upregulation of *P2RX7* mRNA observed in transcriptome profiles from the AMP-AD consortium. P2X_7_R protein increases mirror advancing Braak stage and coincide with synapse loss. Using RNAScope we detect *P2RX7* mRNA in microglia and astrocytes in human AD brain, including in the vicinity of senile plaques. In cultured microglia, P2X_7_R activation modulates the NLRP3 inflammasome pathway by promoting the formation of active complexes and release of IL-1β. In astrocytes, P2X_7_R activates NFκB signalling and increases production of the cytokines CCL2, CXCL1 and IL-6 together with the acute phase protein Lcn2. To further explore the role of P2X_7_R in a disease-relevant context, we expressed wild-type or FTD-causing mutant forms of tau in mouse organotypic brain slice cultures. Inhibition of P2X_7_R reduces insoluble tau levels without altering soluble tau phosphorylation or synaptic localisation, suggesting a non-cell autonomous role of glial P2X_7_R on pathological tau aggregation. These findings support further investigations into the cell-type specific effects of P2X_7_R-targeting therapies in tauopathies.

## Highlights

• P2X_7_R mRNA and protein increase with disease prior to substantial synaptic loss.
• P2X_7_R induces distinct inflammatory signalling in astrocytes and microglia.
• Specific P2X_7_R antagonism in tauopathy models reduces tau aggregate burden.
• P2X_7_R contributes to disease pathogenesis likely by non-cell autonomous mechanisms.

## 1 Introduction

Tauopathies, including Alzheimer’s disease (AD) and some forms of frontotemporal dementia (FTD), are characterised by heterogenous deposition of phosphorylated tau aggregates in different neural cell types and brain regions (Kovacs, 2020). AD, which also shows extensive amyloid-β (Aβ) accumulation in senile plaques and perivascular regions, is the most common neurodegenerative disease and the principal cause of senile dementia (Scheltens et al., 2021). The main neuropathological features of tauopathies are accompanied by extensive loss of synapses and neurons (Reid et al., 2020, Serrano-Pozo et al., 2011) alongside complex changes in microglial (Paolicelli et al., 2022) and astrocytic (Escartin et al., 2021) phenotypes and functions, which are considered key contributors to the onset and progression of disease (Caamano-Moreno and Gargini, 2022, Henstridge et al., 2019, Verkhratsky et al., 2019).

Brain injury, associated chronic neuroinflammatory responses, and neurodegeneration are accompanied by the release of purines such as ATP into the extracellular milieu, where they are recognised by purinergic receptors (P2Rs) to induce intracellular signalling (Bours et al., 2006, Verkhratsky and Burnstock, 2014, Volonte et al., 2003). Among P2R subtypes, P2X_7_R is unique within the family in that it is activated by comparatively high concentrations of ATP (EC_50_ ∼ 100 µM) (Khakh and North, 2012) which allows the flux of cations. Excessive or prolonged stimulation of P2X_7_Rs leads to channel pore dilatation, allowing passage of small (900 Da) hydrophilic molecules (Kopp et al., 2019, McCarthy et al., 2019). Peripheral functions of P2X_7_R include regulation of immune responses and cytokine release amongst others (Di Virgilio et al., 2017). P2X_7_R is also expressed in the CNS and although the cell-type specific localisation and signalling of P2X_7_R in the brain remain incompletely understood, P2X_7_R is implicated in several neurological diseases with altered glial responses including AD and other tauopathies (Carvalho et al., 2021, Beltran-Lobo et al., 2023).

*P2RX7* mRNA and protein are upregulated in end-stage post-mortem AD brain, although no correlation between histopathological (Braak) disease stage and receptor expression was ever analysed (di Lauro et al., 2022, Martinez-Frailes et al., 2019, Martin et al., 2019, McLarnon et al., 2006); likewise P2X7Rs are upregulated in mouse models of amyloidosis (Lee et al., 2011, Ryu and McLarnon, 2008) and tauopathies (Carvalho et al., 2021). Pharmacological inhibition or genetic knockout of P2X_7_R reduces Aβ burden (Diaz-Hernandez et al., 2012, Martin et al., 2019), misfolded (Ruan et al., 2020) and other modified forms of tau (Carvalho et al., 2021, di Lauro et al., 2022) in mouse models of Aβ or tau pathology. Pharmacological antagonism of P2X_7_R ameliorates cognitive deficits in amyloid precursor protein (APP) transgenic mice and in mice injected with human Aβ (Chen et al., 2014, Martin et al., 2019), as well as in tauopathy mouse models expressing FTD-causing tau mutations (Carvalho et al., 2021, di Lauro et al., 2022). These data strongly implicate P2X_7_R in tauopathy pathogenesis *in vivo*. However, the cell-type specific expression and cell-type specific functional consequences of P2X_7_R activation are still debated (Illes et al., 2017, Miras-Portugal et al., 2017).

In this study, we examined P2X_7_R in tauopathies, showing that increases in P2X_7_R protein with advancing Braak stage coincide with synapse loss in different brain regions, prior to end-stage AD. These findings are supported by transcriptomic data from the Accelerating Medicines Partnership-Alzheimer’s disease (AMP-AD) consortium showing a significant upregulation of *P2RX7* mRNA in AD relative to controls as well as in CRND8 (mutant human APP) and rTg4510 (mutant human tau) -expressing transgenic mice with ageing. We use RNAScope to overcome issues with antibody specificity to show that *P2RX7* mRNA localises to both microglia and astrocytes, including in the vicinity of Aβ plaques in the AD brain. We found that P2X_7_R is an upstream modulator of NLRP3 inflammasome complexes and interleukin (IL)-1β production in microglia whereas in astrocytes, P2X_7_R activation drives NFκB signalling and increases levels of CCL2, CXCL1, IL-6 and lipocalin-2 (Lcn2). Finally, in an *ex vivo* tauopathy model, we show that chronic P2X_7_R blockade markedly reduces pathological tau inclusions without significantly affecting soluble tau phosphorylation or tau localisation at synapses, suggesting the possibility of P2X_7_R involvement in the non-cell autonomous clearance of tau aggregates by glia. These data support further exploration of cell-type specific effects of P2X_7_R antagonists in tauopathies.

## 2 Methods

### 2.1 Post-mortem human brain

Post-mortem human prefrontal cortex Brodmann area 9 (BA9) and temporal cortex (BA21) from non-neurologically impaired donors and pathologically confirmed cases of sporadic AD were obtained from the London Neurodegenerative Diseases Brain Bank at King’s College London. Age-matched cases were investigated as Control (Braak stage 0, I, II), Moderate AD (Braak stage III-IV) and Severe AD (Braak stage V-VI) (Table S1).

### 2.2 Human and mouse RNA-sequencing data

RNA-sequencing (RNA-seq) data from the Mayo cohort including temporal cortex of AD, progressive supranuclear palsy (PSP) and age-matched controls was obtained from the AMP-AD Knowledge Portal (https://adknowledgeportal.org). Experimental details can be found in the data portal’s website. This case-control series collected at the Mayo Clinic Brain Bank and the Banner Sun Health Institute consisted of subjects of European descent with pathologically confirmed AD or PSP, or pathological aged controls, as defined by Allen et al. (2016).

RNA-seq data from CRND8 and rTg4510 mice at 3,6,12 and 20 months or 2.5, 4.5 and 6 months, respectively, were obtained from the AMP-AD Knowledge Portal (https://adknowledgeportal.org). Experimental details can be found in the data portal’s website. CRND8 mice express human APP harbouring the Swedish (K670N and M671L) and Indiana (V717F) familial AD-causing mutations (Chishti et al., 2001). rTg4510 mice regulatably (Tet-off) express human tau harbouring the P301L frontotemporal dementia-causing *MAPT* mutation (Santacruz et al., 2005).

### 2.3 Mouse primary neural cell cultures

Primary glial cultures were prepared from CD1 neonatal mouse pups (P1-P3) and seeded on T75 flasks pre-coated with 50 µg/mL PDL (Sigma-Aldrich) as described (Schildge et al., 2013). To isolate astrocytes, cultures were maintained in complete DMEM (Gibco) supplemented with 10 % (v/v) foetal bovine serum (FBS), 2 mM glutamax and 100 U/mL penicillin/100 µg/mL streptomycin at 37 °C and 5 % CO_2_ until confluent, with overnight shaking at 5 days *in vitro* (DIV) in a humidified CO_2_ incubator shaker at 200 rpm at 37 °C to remove microglia. Astrocyte-enriched cultures were trypsinised in TrypLE (Invitrogen) at 37 °C and seeded at a density of 2x10^5^ cells/well, 4x10^5^ cells/well and 2x10^4^ cells/well on PDL coated 12-, 6- or 96-well plates, respectively, until they reached 12–13 DIV. For microglia, the cell culture medium in T75 flasks was replaced by complete DMEM supplemented with 5 ng/mL GM-CSF (Peprotech) from 7 to 14 DIV to enhance microglial proliferation (Nicol et al., 2018). Microglial-enriched cell suspensions were shaken at 200 rpm for 3 h at 37 °C. The cell suspension was centrifuged at 180 g for 5 min at 21 °C and cells seeded in complete DMEM without GM-CSF in 12- or 96-well PDL-coated plates at 1x10^5^ or 1x10^4^ cells/well respectively, and maintained until 15–16 DIV. Growth medium was replaced by neurobasal serum-free medium (Gibco) supplemented with 2 % (v/v) B27 (Thermo-Fischer), 2 mM glutamax, 100 U/mL penicillin and 100 µg/mL streptomycin 24 h before treatment. Astrocytes were stimulated with 300 µM 2′(3′)-O-4-benzoylbenzoyl-ATP (BzATP) (Sigma-Aldrich) for 4 h. Microglia were primed with 100 ng/ml lipopolysaccharide (LPS) (*E. coli* O26:B6, Sigma-Aldrich) for 3 h, the medium was replaced, and cells were activated with 1 mM ATP (Sigma-Aldrich) for 20 min. For microglial ASC speck formation assays, cells were pre-treated with 100 µM of the caspase-1 inhibitor Ac-YVAD-CMK (Sigma-Aldrich) for 1 h prior to stimulation with ATP (Swanton et al., 2020). Astrocytes and microglia were pre-treated with the P2X_7_R antagonist Compound 2 (C_19_H_17_N_5_), also known as A-804598 (O'Brien-Brown et al., 2017) for 1 h at a range of concentrations prior to the addition of BzATP or ATP. The concentrations of Cp2 used were based on previous publications examining changes in microglial function following P2X_7_R antagonism (He et al., 2017, Janks et al., 2018). All experiments included vehicle-treated (0.1 % DMSO) and untreated controls.

### 2.4 Mouse organotypic brain slice cultures

Organotypic brain slice cultures (BSCs) were prepared from P7-P8 CD1 mouse pups as described (Croft et al., 2017). Three consecutive 350 µm coronal slices per well were cultured in Millicell culture inserts (Merck Millipore) in sterile slice culture medium (19.3 mM NaCl, 5 mM NaHCO_3_, 2.7 mM CaCl_2_·2H_2_O, 2.5 mM MgSO_4_·7H_2_O, 0.5 mM ascorbic acid, 0.9 % (w/v) basal medium eagle, 40 mM glucose, 1 mM HEPES, 1% (v/v) glutamax, 0.5 % (v/v) penicillin/streptomycin, 0.033% (v/v) insulin and 25 % (v/v) horse serum. Three hours after plating, the culture medium was replaced with 1 mL of pre-warmed fresh sterile culture medium containing adeno-associated virus (AAV)2/TM8-WT- hTau0N4R-EGFP (1x10^11^ VGs/mL) (WT human tau or WT hTau) or AAV2/TM8-P301L/S320F-hTau0N4R- EGFP (1x10^11^ VGs/mL) (mutant hTau) as described (Croft et al., 2019, Goodwin et al., 2021). Fresh, virus-free, medium was added after 2 days and every 2–3 days thereafter. After a stabilisation period of 14 DIV, which allowed the acute effects of axotomy to be resolved (Croft and Noble, 2018), region-matched BSCs transduced with WT or mutant hTau were treated with 10 µM Cp2 or 0.1% DMSO (vehicle control). The concentration of Cp2 used here was based on our experiments in cultured mouse microglia and astrocytes when considering the relative thickness of slices and that the drug needs to be bioavailable to all cells. Cp2, or an equivalent volume of DMSO, was diluted in fresh culture media. In addition, a 10 µL drop containing 10 µM Cp2 or vehicle was added on top of each insert, distributed evenly across the three slices. BSCs were chronically treated with six doses of P2X_7_R antagonist or vehicle by exchanging the culture medium with fresh medium containing Cp2 or vehicle from 14 DIV, every 2–3 days, until harvesting at 28 days DIV.

### 2.5 Collection of total protein homogenates/lysates

Frozen post-mortem grey matter from AD (n = 35 BA9, n = 10 BA21) and control (n = 26 BA9, n = 4 BA21) brain was homogenised using a handheld mechanical tissue homogeniser (Omni International) at 100 mg/mL in ice-cold lysis buffer containing 10 mM Tris-HCl (pH 7.5), 75 mM NaCl, 0.5 % sodium dodecylsulphate (SDS) (w/v), 20 mM sodium deoxycholate and 1 % (v/v) Triton-X-100 supplemented with 10 mM ethylenediaminetetraacetic acid (EDTA), protease and phosphatase inhibitors (Roche). Following brief sonication for 10 sec, samples were centrifuged at 25,000 g for 20 min at 4 °C. Supernatants were collected as total homogenates and stored at −80 °C until required.

Microglia and astrocytes were washed once with Dulbecco’s PBS (Gibco) and lysates were collected in RIPA buffer containing 50 mM Tris-HCl (pH 8.0), 150 mM NaCl, 0.1 % (w/v) SDS, 0.5 (w/v) sodium deoxycholate, 1 % (v/v) NP-40, freshly supplemented with 10 mM EDTA, protease and phosphatase inhibitors (Roche).

The protein content was determined by BCA assay (Pierce) and samples were equalised, prior to dilution in 2X SDS sample buffer (National Diagnostics) or lithium dodecyl sulphate (LDS) 4X sample buffer (Invitrogen) and 10X reducing agent (Invitrogen) for total human protein homogenates or cell lysates, respectively and heated to 95 °C for 5 min prior to western blotting. To prevent heat-induced aggregation of membrane proteins such as P2X_7_R, samples were heated at 70 °C for 10 min.

### 2.6 Synaptoneurosome extraction

Synaptoneurosomes (SNS) and cytosolic fractions were isolated as described by Perez-Nievas et al. (2013). Briefly, SNS were prepared from 250 mg of frozen human BA9 brain grey matter (AD n = 35, control n = 25) or six BSCs (pooled from two wells) homogenised in ice-cold buffer A (25 mM HEPES pH 7.9, 120 mM NaCl, 5 mM KCl, 1 mM MgCl_2_, 2 mM CaCl_2_, 1 mM DTT, protease and phosphatase inhibitors) using a Teflon-glass mechanical tissue grinder at 170 rpm. Human tissue was first filtered through 80 µm pore filters. Both human and mouse BSCs were dissociated using 5 µm pore filters and centrifuged at 1000 g for 10 min at 4 °C to pellet the SNS. The supernatant was centrifuged at 100,000 g for 45 min and the supernatant collected as the cytosolic fraction. The SNS-containing pellet was washed in cold buffer A with centrifugation at 1,000 g for 10 min. The pellet was solubilised in buffer B (50 mM Tris pH 7.5, 1.5 % (v/v) SDS, 2 mM DTT) and boiled for 5 min. Following centrifugation at 15,000 g for 15 min, the supernatant was collected as the SNS fraction.

The protein concentration of total, SNS and cytosolic fraction was determined using BCA assays and protein concentration equalised prior to dilution in sample buffer and heating to 95 °C for 5 min.

### 2.7 Isolation of sarkosyl-insoluble tau

Sarkosyl-insoluble tau was isolated from post-mortem human brain (BA9) or mouse BSCs (three pooled wells of 9 slices in total) using previously described methods (Croft et al., 2019). Briefly, human brain or mouse BSCs were harvested in ice-cold homogenisation buffer (50 mM TBS, 10 % (w/v) sucrose, 2 mM EGTA, protease and phosphatase inhibitors) at 100 mg/mL and mechanically dissociated using a handheld mechanical tissue homogeniser (Omni International) or a Teflon glass homogeniser, respectively. The supernatant was collected following centrifugation at 4 °C for 20 min at 13,000 g, and an aliquot retained as the low-speed supernatant (LSS). Sarkosyl was added to the remaining supernatant to a final concentration of 1% and this was nutated at ambient temperature for 30 min prior to centrifugation at 100,000 g for 1 h at 21 °C using an Optima MAX-XP ultracentrifuge (Beckman Coulter). The supernatant was collected as the high-speed supernatant (HSS) containing sarkosyl-soluble tau. The pellet was washed with 1 % sarkosyl in homogenisation buffer and centrifuged at 100,000 g for 15 min at 21 °C. The wash supernatant was discarded and the pellet, containing sarkosyl-insoluble tau was resuspended in 2X SDS sample buffer (National Diagnostics).

### 2.8 SDS-PAGE and western blotting

Equal amounts of protein extracts were separated by electrophoresis using 10 % polyacrylamide gels or 4–12 % Bis-Tris gradient gels (Invitrogen) using Tris-Glycine-SDS (National Diagnostics) or MES SDS (Invitrogen) running buffer, respectively, in XCell SureLock Mini-Cells (Invitrogen) and then transferred to nitrocellulose membranes (GE Healthcare). After blocking with Odyssey Blocking Buffer (Li-Cor) for 1 h at RT, membranes were incubated with primary antibodies overnight at 4 °C.

The following primary antibodies were used: Aβ clone 6E10 (1:200, BioLegend, 803001), Aldh1L1 (1:100, UCDavis/NIH NeuroMab facility, N103/39), β-actin (1:5,000, Abcam, Ab8226), lipocalin-2 (1:500, R&D systems, AF1857), neuronal specific enolase (NSE) (1:10,000, DAKO, M0873), phosphorylated Ser536-NFκB p65 (1:1,000, Cell signalling, 3033), total NFκB p65 (1:1,000, Cell signalling, 6956), P2X_7_R (1:200, Alomone, APR-004), PHF1 (1:1,000, kindly gifted by Peter Davies), PSD-95 (1:1,000, Cell signalling, 3450), synaptophysin (1:1,000, Santa Cruz, Sc-17750), total tau (1:10,000, DAKO, A0034). Membranes were washed in 0.2 % (v/v) TBS-Tween 20 and incubated with fluorophore-conjugated secondary antibodies diluted in TBS for 1 h at RT. Following washing signals were detected with an Odyssey® near infra-red detection scanner (Li-Cor Biosciences, CLX-1638). Signals were visualised and quantified using ImageStudio Lite (Li-Cor). Signals of interest were normalised to a loading control in the same sample.

### 2.9 RNAscope and IHC

5 μM sections from control and AD BA9 were prepared from formalin-fixed paraffin-embedded (FFPE) blocks. FFPE HeLa cell pellet slides were purchased from ACDbio (ACDbio). ISH was performed according to the protocol for the RNAscope 2.5 High Definition – Red Assay (ACDbio). In brief, slides were baked in a HybEz hybridisation system (ACD Bio) or a hybridiser HB-1D oven (Akribis Scientific Limited) at 60 °C for 30 min. FFPE-sections were deparaffinised in xylene and 100 % ethanol. After drying the slides for 5 min at RT, H_2_O_2_ (pre-treatment 1) was added for 30 min or 10 min at ambient temperature for FFPE-human tissue or HeLa cell pellets, respectively. Slides were incubated in 90–100 °C antigen retrieval solution for 30 min, washed in ultrapure water twice, dehydrated using 100 % ethanol and treated with protease plus (pre-treatment 3) for 40 min at 40 °C prior to two further washes in ultrapure water. FFPE-fixed sections were incubated with the corresponding mRNA probes for 2 h at 40 °C after which samples were washed twice in 1X washing buffer. Signal amplification was performed by a series of incubations using six amplification probes (Amp1-6) for alternating periods of 30 and 15 min at 40 °C (Amp1-4) or ambient temperature (Amp5-6) as indicated by the supplier. Detection was performed by incubating the FFPE sections with a substrate mixture of RED-A and RED-B (1:60 ratio) for 10 min at ambient temperature protected from light. Tap water was used to remove excess substrate. To stain nuclei, a 50 % (v/v) Gill’s hematoxylin I solution (Sigma-Aldrich) was applied to the slides for 2 min. Following rinsing in tap water, slides were incubated with 0.02 % ammonia water for 10 s followed by further rinsing. Sections were dried at ambient temperature and mounted using a permanent mounting medium (ACDbio).

After *P2RX7* mRNA detection by RNAscope, sections underwent IHC following an adapted protocol from (Grabinski et al., 2015). In brief, hybridised sections were washed twice in PBS and non-specific antibody binding blocked with 2.5 % (v/v) normal horse serum (NHS) (Vectorlabs) or 0.5 % Triton-X-100 + 2.5 % (v/v) NHS for 1 hr at ambient temperature for labelling with antibodies against GFAP (1:100, DAKO, Z0334), β-III tubulin (1:50, Sigma-Aldrich, T878), CD68 (1:50, DAKO, M0876) or Aβ (1:50, BioLegend, 803001). Tissue sections were incubated overnight at 4 °C with primary antibodies diluted in 1 % (v/v) NHS in PBS. Following PBS washing, sections were incubated with HRP-conjugated polymer IgG secondary antibody (Vectorlabs) or a biotinylated IgG secondary antibody (Vectorlabs) diluted in 1 % (v/v) NHS for 1 h at ambient temperature. The vectastain ELITE ABC reagent (Vectorlabs) was added to sections for 30 min according to the manufacturer’s instructions. DAB (Vectorlabs) and blue-grey HRP (Vectorlabs) chromogenic substrates were prepared and added for 2–10 min. Sections were rinsed in tap water before mounting using permanent mounting medium.

### 2.10 Quantitative PCR (qPCR)

RNA was isolated using the Absolutely RNA Miniprep Kit (Agilent) according to the manufacturer’s instructions, except that precipitation was performed overnight at −20 °C. In total, 600 ng of RNA were reverse-transcribed into cDNA at 37 °C for 1 h in 50 µL reactions using the high-capacity RNA-to-cDNA kit (Applied Biosystems) and heat-inactivated at 95 °C for 5 min. Reverse transcribed material corresponding to 7–8 ng/ µL of cDNA was amplified with Takyon qPCR Master Mix blue dTTP for SYBR (Eurogentec) and the corresponding primers (600 nM each) in a total volume of 20 µL using a Rotor-Gene 6000 cycler (Qiagen). Primer sequences are listed in Supplementary Table S2.

### 2.11 Immunocytochemistry

The number of ASC specks within microglia and the subcellular localisation of NFκB within astrocytes were assessed by immunocytochemistry in cells seeded in 96-well plates. After treatment, cells were washed with DPBS (Gibco) and fixed in 4 % (v/v) PFA for 20 min at RT prior to further washing. Cells were permeabilised and non-specific binding blocked using 0.1 % (v/v) Triton-X-100, 5 % (w/v) BSA in TBS for 1 h at ambient temperature before incubating with an anti-ASC antibody (1:200, Adipogen, AG-25B-0006) or anti-NFκB (p65 subunit, 1:400, Cell signalling, D14E12) overnight at 4 °C. AlexaFluor 488 conjugated secondary antibody (Invitrogen) was added for 1 h at RT. Nuclei were stained with Hoechst-33342. Cells were imaged using a confocal Opera Phenix microscope (Perkin Elmer). The number of ASC specks per well was normalised to the number of nuclei and the mean nuclear intensity of NFκB was normalised to the total cell intensity per well using Harmony High-Content imaging analysis software (Perkin Elmer).

### 2.12 Measurement of cytokines

Human cytokine arrays were performed using equal protein concentrations of the cytosolic fraction of post-mortem brain homogenates (BA9) using the Human Proteome Profiler Array Panel A (R&D systems) according to the manufacturer’s instructions. Positive and negative controls were included in the array to allow signal standardisation and quantitative analysis. Results are expressed as percentage relative to control (Braak 0-II).

IL-1β released into microglial culture supernatant was measured using ELISA DuoSet^TM^ kits (R&D systems) following the manufacturer’s instructions.

### 2.13 Statistical analysis

Data were analysed using Graphpad Prism 8.0. following d’Agostino and Pearson (n > 50) or Shapiro-Wilk (n < 50) normality tests, differences between three or more groups were analysed using a non-parametric Kruskal-Wallis H test with Dunn’s post-hoc analysis or one-way analysis of variance (ANOVA) with Dunnett’s post-hoc analysis. Unpaired student *t*-test or Welch’s *t*-test was used to evaluate differences between two groups under the assumption of equal or unequal variances, respectively. To analyse the effect of two independent variables on the mean of a normally distributed quantitative variable, a two-way ANOVA with Sidak’s multiple comparison test was performed. Data is presented as mean ± standard error of the mean (SEM) or standard deviation (SD) as indicated. Results were considered statistically significant when p-value < 0.05.

## 3 Results

### 3.1 P2X_7_R is increased in AD brain

Prior reports have described an upregulation of *P2RX7* mRNA (McLarnon et al., 2006) and protein (Martin et al., 2019, di Lauro et al., 2022) in end-stage AD relative to non-demented controls, however changes were not related to Braak stage. To expand these findings in a larger cohort of cases and gain insights into P2X_7_R changes at different disease stages, we quantified P2X_7_R protein in Brodmann area (BA) 9 and BA21, regions showing relatively late and early synaptic degeneration, respectively (Dekosky and Scheff, 1990, Scheff and Price, 1993).

Given that several commercially available antibodies against P2X_7_R have been deemed nonspecific (Anderson and Nedergaard, 2006) and some P2X_7_R isoforms escape knockout *in vivo* (Nicke et al., 2009, Sim et al., 2004), a panel of commonly used antibodies against human P2X_7_R were first tested in HEK293 cells transiently expressing FLAG-tagged human P2X_7_R (Fig. S1**a-b**). The antibodies from Novus Biologicals and Thermo-Fisher showed poor specificity in this test, whereas the antibodies from Alomone and Proteintech detected exogenously expressed human P2X_7_R (Fig. S1**c-j**). We therefore selected the P2X_7_R antibody manufactured by Alomone for western blotting of total homogenates from BA9 and BA21.

**Fig. 1:**
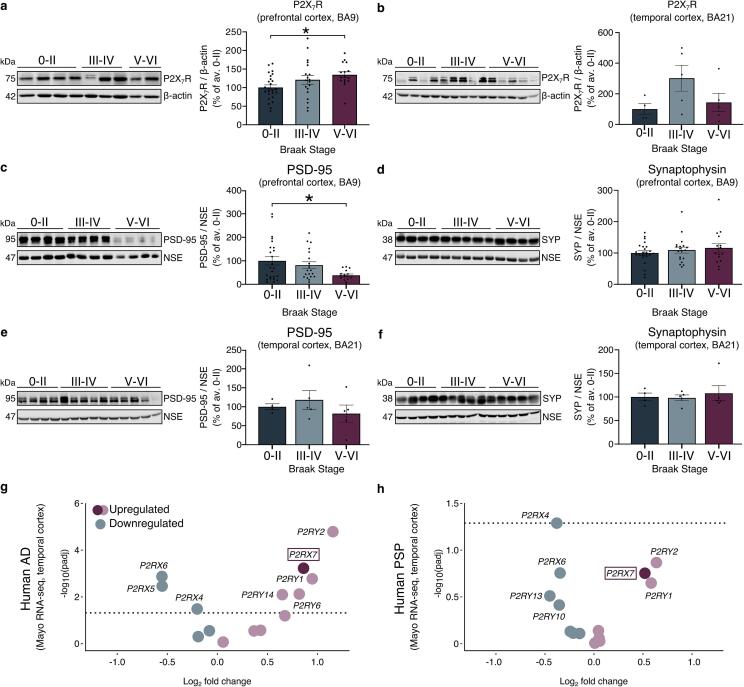
**P2X_7_R expression is elevated prior to end-stage AD.** Representative immunoblots using an antibody against P2X_7_R of total brain homogenates from **(a)** BA9 prefrontal cortex and **(b)** BA21 temporal cortex. β-actin was included as a loading control. Bar charts show quantification of P2X_7_R protein relative to β-actin in each sample as percentage of average control (Braak 0-II). Representative immunoblots of the post-synaptic and pre-synaptic markers **(c,e)** PSD-95 and **(d, f)** synaptophysin (SYP) in BA9 and BA21 total homogenates. Bar charts show quantification of synaptic proteins relative to neuron-specific enolase (NSE) in each sample as percentage of average control (Braak 0-II). Data is mean ± SEM. Following **(a,c,d)** d’Agostino and Pearson or **(b,e,f)** Shapiro-Wilk normality tests, data was analysed using a parametric one-way ANOVA with Dunnett́s multiple comparison test or a non-parametric Kruskal-Wallis test with Dunńs multiple comparison test. **(a,c,d)** n = 25 (Braak 0-II), 19 (Braak III-IV), 16 (Braak V-VI). **(b,e,f)** n = 4 (Braak 0-II), n = 5 (Braak III-IV), n = 5 (Braak V-VI). *p < 0.05. **g,h)** Volcano plots showing changes in expression of *P2R* transcripts in temporal cortex from **(g)** AD and **(h)** PSP brain versus age-matched controls obtained from the Mayo RNA-seq cohort of the AMP-AD consortium. Dotted lines indicate the cut-off p-value of 0.05.

Synaptic malfunction is the best correlate of neurodegeneration and the clinical symptoms of AD (McInnes et al., 2018), and BA9 is one of the brain regions last affected by synaptic degeneration in AD (DeKosky and Scheff, 1990). We examined P2X_7_R in BA9 to determine changes that occur prior to substantial synapse loss and neurodegeneration. Characterisation of BA9 samples showed the expected accumulation of tau and Aβ with advancing stage, including at synapses, alongside morphological changes in Aβ-associated GFAP^+^-astrocytes and Iba1^+^-microglia (Fig. S2). We found that P2X_7_R protein was significantly elevated in BA9 from Braak stage V-VI cases relative to Braak 0-II controls **(**Fig. 1**a)**. Examination of the temporal cortex (BA21), which is affected earlier and more significantly, showed an apparent but non-significant transient increase in P2X_7_R levels in Braak III-IV samples relative to Braak 0-II controls and Braak V-VI samples (Fig. 1**b**). Immunoblotting of the pre- and post-synaptic markers, synaptophysin (SYP) and post-synaptic density (PSD)-95 in BA9 samples showed a significant loss of PSD-95 at late Braak stages and no significant changes in synaptophysin between groups **(**Fig. 1**c,d)**. In BA21, there was a trend towards decreased PSD-95 in Braak V-VI samples relative to controls, but no apparent changes in synaptophysin **(**Fig. 1**e,f)**. These data emphasise the importance of region-specific studies when examining P2X_7_R levels in AD brain and suggest that increases in P2X_7_R protein occur alongside or prior to the initial stages of synapse loss in distinct regions and are not solely a feature of advanced disease.

To independently confirm P2X_7_R upregulation in AD, we examined transcriptional changes in members of the P2R family, including ionotropic P2XRs and metabotropic P2YRs (Fields and Burnstock, 2006), using the AMP-AD Mayo RNA-seq dataset (Allen et al., 2016). We observed a significant upregulation of *P2RX7* mRNA in AD temporal cortex relative to aged controls, alongside alterations in other P2X (4,5,6) and P2Y (1,2,6,14) receptors **(**Fig. 1**g**, Table S3**)**. Similarly, *P2RX7* was also found among the most upregulated transcripts in the primary tauopathy progressive supranuclear palsy (PSP) relative to controls, although this did not reach statistical significance **(**Fig. 1**h,** Table S3**)**.

### 3.2 *P2RX7* mRNA is expressed in astrocytes and microglia proximal to plaques in AD brain

To better understand which neural cells upregulate P2X_7_R in AD, we characterised the cell-type specific localisation of P2X_7_R in the human brain, an issue that remains controversial in the field (Illes et al., 2017, Miras-Portugal et al., 2017). Since the P2X_7_R antibody validated *in vitro* for use in western blot did not yield conclusive immunolabelling of human brain sections, RNAscope *in situ* hybridisation (ISH) was used as an orthogonal method to map the cell-specific distribution of *P2RX7* mRNA in human control and AD brain in combination with immunostaining of neuronal and glial markers. Preliminary testing of RNAscope using probes against *PPIB, UBS-C* (positive controls) and *DapB* (negative control) in HeLa cells and human brain sections validated the assay workflow and confirmed an optimal mRNA quality and accessibility which allowed the specific detection of *P2RX7* mRNA in human brain sections (Fig. S3).

**Fig. 2:**
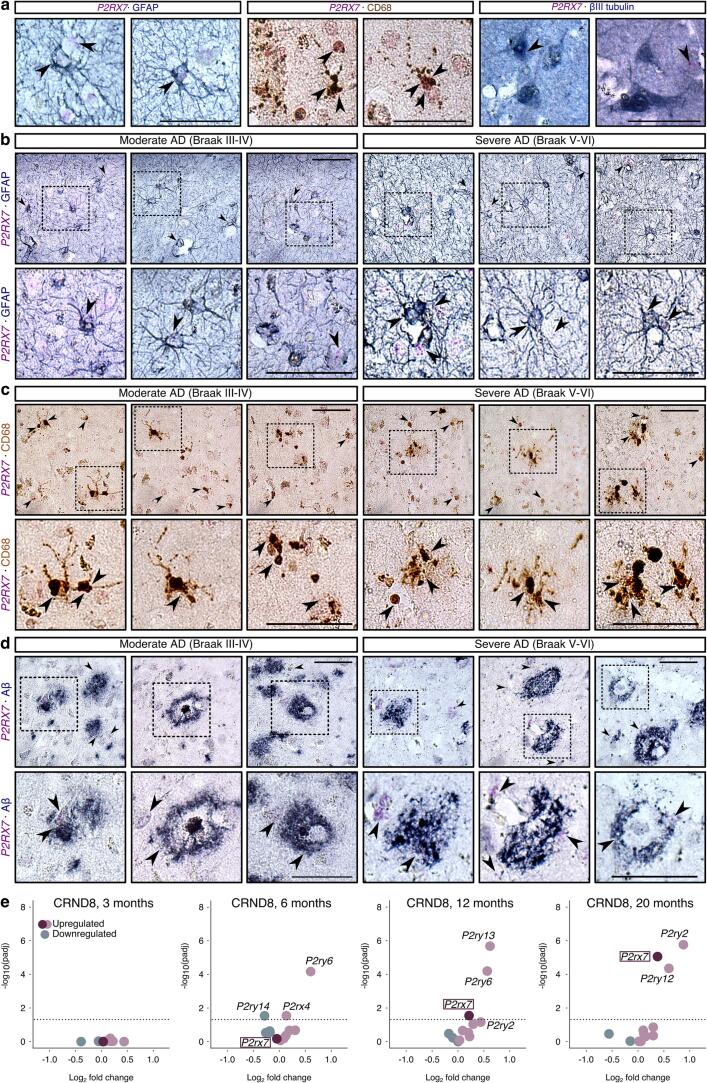
***P2RX7* mRNA localises to GFAP^+^ astrocytes and CD68^+^ microglia surrounding Aβ plaques in AD brain. a)** Representative *in-situ* hybridisation-immunohistochemistry (ISH-IHC) images of brain sections from frontal cortex (BA9) of human control brain (Braak stage 0-II) hybridised with RNAscope probes against *P2RX7* mRNA (red puncta, indicated by arrowheads) and immunolabelled with antibodies against GFAP and βIII-tubulin (blue/grey chromogenic stain) and CD68 (diaminobenzidine [DAB] stain). n = 3. Representative images of *P2RX7* RNAscope performed in sections from three Braak stage III-IV and three Braak stage V-VI AD cases immunolabelled with antibodies against **(b)** GFAP**, (c)** CD68 and **(d)** Aβ (6E10). Arrowheads indicate representative *P2RX7* mRNA puncta in GFAP or CD68 immunopositive cells, or in the vicinity of Aβ deposits. Insets indicate regions of each image displayed at a higher magnification. n = 3. Scale bar: 10 µm. e) Volcano plots showing changes in expression of *P2r* transcripts in transgenic CRND8 mouse brains versus non-transgenic controls at 3, 6, 12 and 20 months obtained from the AMP-AD Knowledge Portal (https://doi.org/10.7303/syn3157182). Dotted lines indicate the cut-off p-value of 0.05. (For interpretation of the references to colour in this figure legend, the reader is referred to the web version of this article.)

*P2RX7* mRNA in human control frontal cortex sections (Braak 0-II) overlapped or was observed in the immediate vicinity of CD68^+^ microglia and GFAP^+^ astrocytes, with few signals observed in β-III tubulin^+^ neurones **(**Fig. 2**a)**. In Braak stages III-IV and V-VI AD brain, *P2RX7* mRNA was detected in the cell body and processes of GFAP^+^ astrocytes in the grey matter and in astrocytic endfeet surrounding cerebral capillaries **(**Fig. 2**b)**. *P2RX7* was abundant in CD68^+^ cells of myeloid linage including ramified microglia present in Braak III-IV cases and as clusters of cells with an enlarged cell body and shortened ramifications in Braak V-VI tissues **(**Fig. 2**c)**, following a similar distribution to that observed surrounding Aβ deposits as shown in Fig. S2 and described by others (Franco-Bocanegra et al., 2019). Indeed, *P2RX7* was found proximal to diffuse and dense-core plaques in AD (Fig. 2**d**). Due to current limitations of the RNAScope technology in combination with immunohistochemistry for glial markers, alongside with poor P2X_7_R antibody specificity, it is technically challenging to accurately determine the reactivity states of glia in which P2X_7_R is expressed. Nevertheless, our results support an astrocytic and microglial P2X_7_R localisation, including proximal to Aβ accumulations in AD brain.

Notably, RNA-seq data from CRND8 transgenic mice over-expressing human *APP* (K670N/M671L and V717F mutations) (Chishti et al., 2001, McFarland et al., 2021) showed an age-dependant increase in *P2rx7* mRNA **(**Fig. 2**e,** Table S4), coinciding with progressive amyloidopathy, which further supports an association between P2X_7_R and senile plaques in AD, in agreement with previous reports (Martin et al., 2019, Martinez-Frailes et al., 2019).

### 3.3 P2X_7_R activation induces distinct signalling in microglia and astrocytes

To better understand the signalling pathways induced upon P2X_7_R stimulation, primary astrocyte and microglia cultures were exposed to P2X_7_R ligands. We first confirmed that both of these cell types express P2X_7_R protein (Fig. S4**a-c**).

**Fig. 3:**
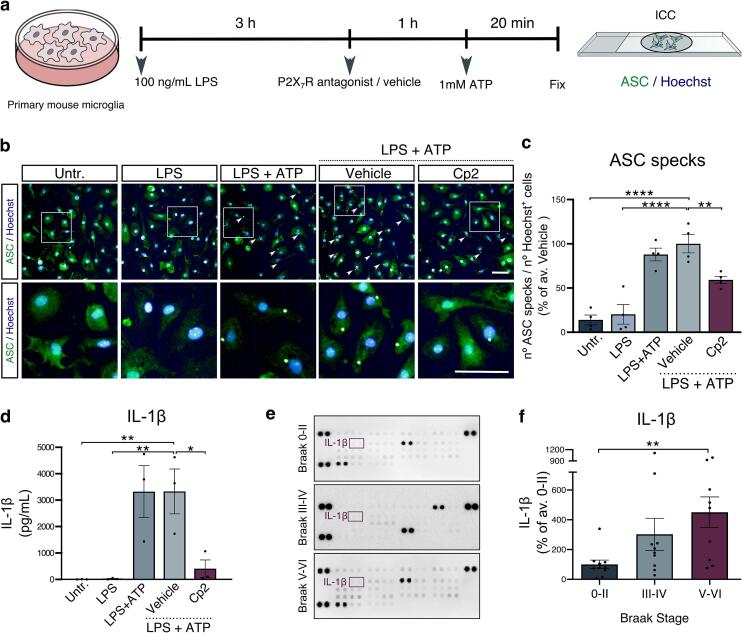
**Microglial P2X_7_R activation induces the formation of ASC specks and release of IL-1β. a)** Schematic depicting the stimulation of microglia with 100 ng/mL LPS for 3 h, after which medium was replaced and cells pre-treated with 0.1% DMSO (vehicle) or 1 µM Cp2 for 1 h prior to the addition of 1 mM ATP for 20 min. Untreated and LPS-primed cells were included as additional controls. **b)** Representative confocal images of microglia immunolabelled using an antibody against ASC (green) under basal conditions (untr.), following stimulation with LPS only, LPS and ATP only, or with LPS and ATP in the presence of vehicle (DMSO) or Cp2. ASC specks are indicated by white arrowheads. Hoechst-33342 was used as to stain nuclei. Insets indicate representative regions of each image displayed at higher magnification. Scale bar: 50 µm. **c)** Quantification of the number of ASC specks normalised to the number of Hoechst^+^ nuclei per condition, displayed as a percentage relative to vehicle-treated cells exposed to LPS + ATP (n = 4). **d)** Bar graph shows quantification of the amounts of IL-1β in the supernatant of microglial cultures primed with LPS, followed by pre-treatment with vehicle or Cp2 and stimulated with ATP. Untreated, LPS-primed and LPS + ATP only conditions were also included (n = 3). **e)** Representative cytokine array of cytosolic fractions isolated from post-mortem BA9 AD and control brain at different Braak stages (0-II, III-IV, V-VI). IL-1β coordinates are indicated in purple. **f)** Quantification of IL-1β amounts in Braak stage III-IV and V-VI BA9 displayed relative to Braak 0-II tissues. n = 10 per group (Braak 0-II, III-IV, V-VI). Following Shapiro-Wilk normality test, data was analysed using **(c,d)** one-way ANOVA with Dunnett’s multiple comparison test or **(f)** Kruskal-Wallis test with Dunńs multiple comparison test. Data is mean ± SEM. *p < 0.05, **p < 0.01,****p < 0.0001. (For interpretation of the references to colour in this figure legend, the reader is referred to the web version of this article.)

P2X_7_R acts as an ATP-dependant upstream modulator of the NOD-, LRR- and pyrin domain-containing protein 3 (NLRP3) inflammasome pathway in macrophages (Mariathasan et al., 2006). To investigate the P2X_7_R-mediated regulation of inflammasome complexes in microglia, cells were stimulated with ATP after priming with the bacterial endotoxin LPS. Priming with LPS induced the expression of the inflammasome sensor NLRP3 **(**Fig. S4**d)**. P2X_7_R-mediated effects were dissected by exposing microglial cultures to the specific P2X_7_R antagonist compound 2 (Cp2) (O'Brien-Brown et al., 2017) prior to stimulation (Fig. 3**a**). The formation of oligomerised forms of the apoptosis-associated speck-like protein with CARD domain (ASC), referred to as ASC specks (Stutz et al., 2013), which are also immunoreactive for NLRP3 **(**Fig. S4**e)**, were evaluated as a readout of NLRP3 inflammasome complex formation. ATP evoked a significant increase in the number of ASC specks relative to untreated and LPS-primed microglia. Pre-treatment with Cp2 reduced the number of ASC specks by approximately 50% indicating that P2X_7_R activation in microglia contributes to the formation of ASC specks in response to ATP (Fig. 3**b,c**). To confirm that P2X_7_R activation leads to the formation of active oligomerised inflammasome complexes, levels of IL-1β, one of the end-products of the inflammasome pathway and potent pro-inflammatory cytokine (Swanton et al., 2018) were examined in culture medium. In microglia primed with LPS, stimulation with ATP elicited a significant increase in the secretion of IL-1β into the cell culture supernatant relative to the amounts detected under basal conditions or following LPS priming alone. Pharmacological inhibition of P2X_7_R reduced the levels of secreted IL-1β (Fig. 3**d**). We also examined inducible nitric oxide synthase (iNOS) and tumour necrosis factor α (TNFα), which are transcriptionally regulated (Raffaele et al., 2020, Saha et al., 2006) central mediators of microglial inflammatory responses (Bachiller et al., 2018, Starossom et al., 2012, Combs et al., 2001). Microglial priming with LPS increased mRNA levels of TNFα and iNOS, however these changes were not affected by Cp2 treatment and therefore appear to be independent of ATP-mediated P2X_7_R activation **(**Fig. S4**f,g)**. Nonetheless, Cp2 treatment caused an apparent, but not significant, decrease in the levels of lactate dehydrogenase (LDH) released from LPS + ATP-treated cells, suggesting that P2X_7_R blockade leads to protection following microglial stimulation (Fig. S4**h**). These results are therefore consistent with a specific role of P2X_7_R in the activation of the NLRP3 inflammasome upon exposure to ATP in primed microglia. Notably, like P2X_7_R protein, IL-1β levels were also found to be increased in homogenates of Braak stage V-VI BA9 tissue samples relative to that of age-matched controls (Braak 0-II) (Fig. 3**e,f**), in agreement with findings from others (Griffin et al., 1989). Moreover, the NLRP3 inflammasome-processed cytokine IL-18 (Swanton et al., 2018) was also found upregulated in severe AD (Braak V-VI) tissues alongside other mediators (summarised in Table S5).

**Fig. 4:**
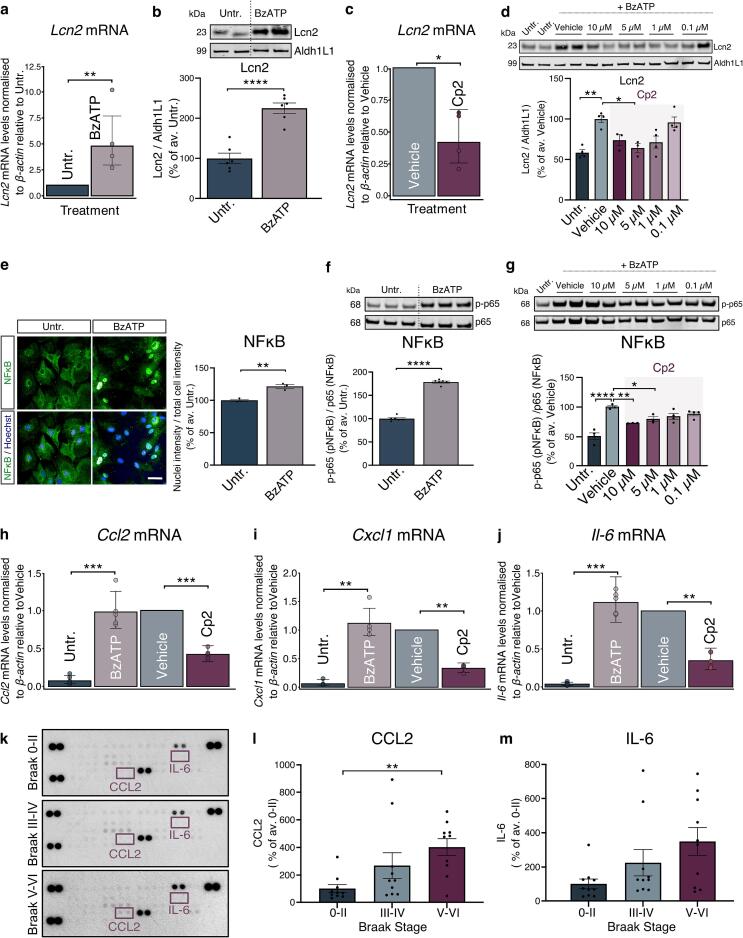
**Astrocytic P2X_7_R regulates the expression of Lcn2, activates NF**κ**B and induces cytokine production. a)** mRNA levels of *Lcn2* in astrocytes stimulated with BzATP (300 µM, 4 h) expressed as relative abundance to control (n = 4). **b)** Representative immunoblots of Lcn2 and Aldh1L1 in control or BzATP stimulated astrocytes. Bar graph shows the quantification of Lcn2 levels normalised to Aldh1L1 in BzATP stimulated astrocytes expressed as percentage of control (n = 6). **c)** mRNA levels of *Lcn2* in astrocytes pre-treated with 0.1 % DMSO (vehicle) or the P2X_7_R antagonist Cp2 (1 µM) for 1 h prior to stimulation with BzATP expressed as relative abundance to control (n = 4). **d)** Representative immunoblots of Lcn2 and Aldh1L1 in vehicle-treated or astrocytes treated with the indicated concentrations of Cp2 for 1 h prior to stimulation with BzATP. Bar graph shows the quantification of Lcn2 normalised to Aldh1L1 expressed relative to vehicle (n = 3–4). **e)** Representative images of NFκB labelling (green) in control and BzATP stimulated astrocytes (upper panel). Hoechst-33342 was used as a nuclear stain. Merged images are displayed in the lower panel. Scale bar: 50 µm. Bar chart shows quantification of the mean nuclear intensity of NFκB relative to mean total cell intensity per well expressed relative to control (n = 3). **f-g)** Representative immunoblots of Ser536 phosphorylated (p-)NFκB p65 subunit and total NFκB (p65) in **(f)** BzATP-stimulated and control astrocytes and **(g)** unstimulated astrocytes, treated with vehicle or with the indicated concentrations of Cp2 for 1 h prior to stimulation with BzATP. Bar graph displays the quantification of p-NFκB normalised to NFκB relative to **(f)** untreated (n = 6) or **(g)** vehicle (n = 3–4). **h-j)** mRNA levels of **(h)***ccl2* (n = 5)*,***(i)***cxcl1* (n = 4) **(j)***il-6* (n = 5) in control astrocytes and astrocytes stimulated with BzATP, treated with vehicle or Cp2 (1 µM) for 1 h prior to stimulation with BzATP. Data is expressed as relative abundance to vehicle. **k)** Representative cytokine array from BA9 control and AD brain at different Braak stages (0-II, III-IV, V-VI). CCL2 and IL-6 coordinates are indicated in purple. n = 10 per group. **l-m)** Quantification of **(l)** CCL2 and **(m)** IL-6 amounts in Braak III-IV and V-VI AD BA9 relative to Braak 0-II. Following a Shapiro-Wilk normality tests, **(a,c,h-j)** p-values were obtained from log-transformed values using unequal variance Welch́s *t*-test. Data is mean ± SD. Data analysed using **(b,e,f)** unpaired *t*-test, **(d,g)** one-way ANOVA with Dunnett́s multiple comparison test or **(l, m)** Kruskal-Wallis test with Dunńs multiple comparison test**.** Data is mean ± SEM. *p < 0.05, **p < 0.01, ***p < 0.001, ****p < 0.0001. (For interpretation of the references to colour in this figure legend, the reader is referred to the web version of this article.)

In astrocytes, P2X_7_R is linked with priming to inflammatory stimuli (Lagos-Cabre et al., 2017) and cytokine dysregulation (Munoz et al., 2020, Panenka et al., 2001). To further explore whether P2X_7_R activation alters astrocyte reactivity (Escartin et al., 2021) and signalling, the ATP analogue and potent P2X_7_R agonist BzATP (Khakh and North, 2012) was used to stimulate P2X_7_R in primary astrocytes in the presence and absence of Cp2. Levels of the reactive astrocyte marker lipocalin-2 (Lcn2) (Suk, k. , 2016, Zamanian et al., 2012), a sensitive readout of astrocyte reactivity *in vitro* (Staurenghi et al., 2021) were first evaluated. Exposure to BzATP (300 µM, 4 h) resulted in elevated mRNA and protein levels of Lcn2 (Fig. 4**a,b**), both of which were significantly reduced upon P2X_7_R inhibition with Cp2 (Fig. 4**c,d**).

Since P2X_7_R activates the NLRP3 inflammasome in microglia (Fig. 3), we initially sought to evaluate whether P2X_7_R could also modulate this pathway in astrocytes (Freeman et al., 2017). However, we failed to detect any constitutive or BzATP-induced expression of NLRP3, or the formation of ASC specks upon exposure of astrocytes to BzATP **(**Fig. S4**i,j),** suggesting that primary mouse astrocytes lack a functional NLRP3 inflammasome, at least under the conditions evaluated here.

NFκB induces central pro-inflammatory cascades associated with astrocyte responses in AD (Bales et al., 1998, Kitamura et al., 1997). BzATP treatment enhanced nuclear localisation of NFκB in astrocytes, alongside a two-fold increase in phosphorylation of the p65 subunit of NFκB (Fig. 4**e,f)** which was significantly reduced in a dose-dependent manner upon pre-treatment with Cp2 (Fig. 4**g**). This effect occurred in the absence of cell death as measured by LDH release (Fig. S4**k**) and is consistent with a role for P2X_7_R in the activation of NFκB in astrocytes. Notably, the phosphorylation status and subcellular localisation of NFκB remained unaltered in primary microglial cultures exposed to BzATP (Fig. S4**l,m**), suggesting that the P2X_7_R-mediated modulation of NFκB is exclusive to astrocytes, at least under these conditions. Further evaluation of downstream inflammatory signals showed that BzATP increased the production of the chemokine C-C motif ligand 2 (CCL2), chemokine C-X-C motif ligand 1 (CXCL1) and IL-6 mRNA in astrocytes, and their production was attenuated upon P2X_7_R antagonism with Cp2 (Fig. 4**h-j**). We have previously shown that CXCL1 is elevated in the BA9 region of AD brain (Perez-Nievas et al., 2021). Here we further show that CCL2 protein is upregulated in BA9 from AD (Braak V-VI) relative to controls (Braak 0-II), while IL-6 protein followed an increasing trend that did not reach statistical significance (Fig. 4**k-m).**

These data suggest that P2X_7_R induces distinct signalling in astrocytes and microglia that could participate in the neuropathological process in AD. Specifically, P2X_7_R is linked to NLRP3 inflammasome activation in microglia and is associated with NFκB-driven responses in astrocytes.

### 3.4 P2X_7_R antagonism reduces tau aggregate load ex vivo

**Fig. 5:**
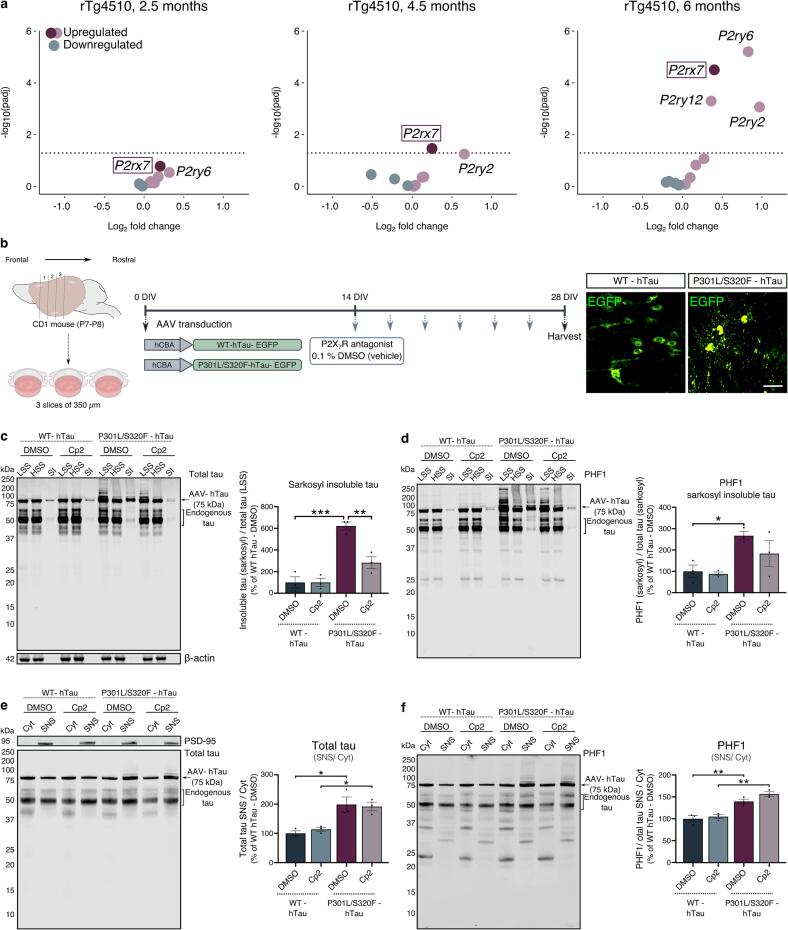
**P2X_7_R inhibition reduces tau aggregate levels in BSCs expressing P301L/S320F human tau. a)** Volcano plots showing changes in expression of *P2r* transcripts in cortex from rTg4510 mice versus non-transgenic controls at 2.5, 4.5 and 6 months, obtained from the AD-AMP Knowledge Portal (https://doi.org/10.7303/syn3157182). Dotted lines indicate the cut-off p-value of 0.05. **b)** Schematic represents the preparation of organotypic brain slice cultures (BSCs) from CD1 mice at P7-P8. BSCs were transduced with 1x10^11^ vg/mL rAAV2/8 expressing EGFP-tagged 0N4R WT or (P301L/S320F) human tau (hTau) under the hybrid cytomegalovirus enhancer chicken β-actin (hCBA) promoter at 0 DIV. Region-matched slices were treated with 10 µM Cp2 at 14 DIV and every 2–3 days thereafter with each media change until 28 DIV. Control slices were treated identically with vehicle (0.1 % DMSO). Representative confocal images of BSCs transduced with EGFP-tagged WT or P301L/S320F-hTau at 28 DIV. Scale bar: 50 µm. Representative immunoblots of low-speed supernatant (LSS), high-speed supernatant (HSS) and sarkosyl-insoluble (SI) fractions probed with antibodies against **(c)** total tau (DAKO), β-actin and **(d)** tau phosphorylated at Ser396/404 (PHF1). Bar graphs display the quantification of **(c)** insoluble tau, determined as the proportion of total tau in the SI fraction relative to total tau in LSS from the same sample and **(d)** phosphorylated insoluble tau, calculated as the amount of PHF1 relative to total tau in the SI fraction, shown relative to vehicle-treated slices expressing WT-hTau. Representative immunoblots of synaptoneurosome (SNS) and cytosolic (cyt) fractions of BSCs immunoblotted with antibodies against **(e)** total tau, PSD-95 and **(f)** PHF1. Bar charts display the ratio of **(e)** total tau and **(f)** PHF1-immunoreactive tau normalised to total tau in SNS relative to the cyt compartment, expressed as percentage relative to BSCs expressing WT-hTau and treated with DMSO. Following Shapiro-Wilk normality test, data was analysed using two-way ANOVAs with Sidaḱs multiple comparison test. n = 3. Data is mean ± SEM. *p < 0.05, **p < 0.01,***p < 0.001.

Given the upregulation of P2X_7_R in human tauopathies (Fig. 1), we sought to explore transcriptomic changes in rTg4510 transgenic mice overexpressing mutant human P301L tau (Ramsden et al., 2005, Santacruz et al., 2005). While a non-significant upregulation in *P2rx7* mRNA was observed at 2.5 months, we uncovered an age-dependant increase in *P2rx7* transcripts from 4.5 months relative to wild-type littermate controls (Fig. 5**a**, Table S6). The *P2rx7* mRNA increases coincide with the progressive deposition of tau pathology in these mice and occur prior to synapse and neuron loss (Ramsden et al., 2005, Santacruz et al., 2005). This upregulation together with evidence that P2X_7_R modulates signalling cascades in astrocytes and microglia that are implicated in pathological changes in tau (Joly-Amado et al., 2020, Kitazawa et al., 2011, Mann et al., 2022, Perez-Nievas et al., 2021) prompted us to examine the effects of P2X_7_R antagonism in a disease-relevant model of tauopathy.

For this purpose, organotypic mouse brain slice cultures (BSCs) were transduced with viral vectors to drive the expression of EGFP-tagged WT 0N4R human tau or mutant 0N4R human tau carrying the FTD-causing *MAPT* mutations P301L and S320F which show increased propensity to aggregate *in vitro* (Rosso et al., 2002, Strang et al., 2018) and *ex vivo* (Croft et al., 2019). These constructs are referred to as WT-hTau or P301L/S320F-hTau, respectively **(**Fig. 5**b)**. Expression of P301L/S320F-hTau, but not WT-hTau leads to the rapid accumulation of hyperphosphorylated and sarkosyl-insoluble tau alongside dense somatic tau inclusions, as previously described (Croft et al., 2019) (Fig. 5**Sa-e**).

Treatment of BSCs with 10 µM Cp2 at every media change (every 2–3 days) from 14 DIV until harvesting at 28 DIV led to a significant reduction in the amounts of insoluble (aggregated) tau in P301L/S320F-hTau expressing slices, relative to vehicle-treated control (Fig. 5**c**). Quantification of total and phosphorylated tau species in the low-speed supernatant (LSS), containing both soluble and insoluble forms, and soluble tau species in the high-speed supernatant (HSS) did not show any effects of Cp2 treatment when compared to vehicle-treated BSCs (Fig. S5**e-h**). In line with the apparent lack of effect on tau phosphorylation at the PHF1 epitope (Fig. 5**d**), P2X_7_R antagonism did not reduce the accumulation of synaptic tau observed in BSCs expressing P301L/S320F-hTau **(**Fig. 5**e,f)**. To explore this further, we examined the association between P2X_7_R levels and soluble or insoluble tau in samples of BA9 from human post-mortem AD brain. While there was no apparent correlation between P2X_7_R levels and insoluble PHF1^+^ tau in the BA9 region of AD brain, a significant inverse correlation was found between P2X_7_R and the abundance of soluble phosphorylated tau (Fig. S6), demonstrating that when P2X_7_R levels are high, there is less soluble tau in AD brain. Together, these data provide evidence that P2X_7_R-driven activity specifically affects the abundance of aggregated disease-associated forms of tau, perhaps suggesting a non-cell autonomous role for glial P2X_7_R in the accumulation or persistence of tau aggregates.

## 4 Discussion

In the present study we have explored the expression pattern and distribution of P2X_7_R in AD at different Braak stages and control brains, and we investigated the molecular pathways downstream of P2X_7_R activation in glia. Furthermore, we evaluated the therapeutic potential of P2X_7_R antagonism in tauopathies by assessing the effects of chronic P2X_7_R blockade in an *ex vivo* tauopathy model. Our data shows that P2X_7_R is upregulated with increasing Braak stage in BA9 AD brain relative to controls. The upregulation observed in our large cohort (n = 60) of AD and control cases provides robust support for previous biochemical and histology-based studies that have described increase in P2X_7_R in AD in smaller sample sets (di Lauro et al., 2022, Martin et al., 2019, Martinez-Frailes et al., 2019). We also extended previous observations by showing that expression of P2X_7_R increases with histopathological Braak stages of the disease. The elevated levels of P2X_7_R were detected in parallel with the mislocalisation of pathological tau species to the synapses and the loss of post-synaptic markers in BA9 AD brain suggesting that P2X_7_R upregulation could be an early event preceding overt neurodegeneration.

Small, but not significant increases in P2X_7_R were also observed at earlier Braak stages in BA21, a region that shows synaptic degeneration before BA9 (DeKosky and Scheff, 1990). While the small set of BA21 samples used here did not reveal significant synaptic protein loss, we and others have demonstrated this previously in temporal lobe using larger sample sets (Kurbatskaya et al., 2016, Perez-Nievas et al., 2013). Increases in P2X_7_R were reflected at the transcriptomic level (RNA-seq dataset from the AMP-AD consortium), revealing a significant upregulation of *P2RX7* in temporal cortex in AD relative to age-matched controls. In line with the human data, *P2rx7* displayed an age-dependant increase in the mouse models of amyloidosis CRND8 (Chishti et al., 2001) and tauopathy rTg4510 (Santacruz et al., 2005) as the neuropathological burden progresses.

Furthermore, our study provides evidence for the localisation of *P2RX7* mRNA in microglia and astrocytes surrounding Aβ deposits in AD brain. Although this strongly advocates for the presence of P2X_7_R protein in these cell-types, it should be noted that P2X_7_R expression and function can be fine-tuned via both post-transcriptional and post-translational mechanisms (Sluyter, 2017). The mechanisms underlying the increase in P2X_7_R in disease remain unclear but may reflect damage-induced increases in AD brain of specific protein 1 (SP1) (Citron et al., 2008), a transcription factor that regulates the expression of P2X_7_R in astrocytes and microglia (Garcia-Huerta et al., 2012, Qin et al., 2017). An important future goal is to uncover the association between P2X_7_R and functional glial subtypes in AD by quantitatively examining the expression of *P2RX7* in disease-associated subpopulations of astrocytes (Habib et al., 2020), microglia (Mathys et al., 2019) or oligodendrocytes (Sadick et al., 2022), where *P2RX7* might also be expressed (Pietrowski et al., 2021, Lau et al., 2020). However, reactive astrocytes and microglia are molecularly heterogeneous and dynamic responses (Escartin et al., 2021, Paolicelli et al., 2022) and this complexity is difficult to accurately capture using RNAscope in combination with classical immunohistochemical methods.

Increased activity of P2X_7_R may result from elevated protein levels and could also be mediated by ATP released from cells in response to Aβ or tau accumulation, including autocrine or paracrine stimulatory loops (Carvalho et al., 2021, di Lauro et al., 2022, Ruan et al., 2020, Sanz et al., 2009, Orellana et al., 2011, Volonte et al., 2003). One hypothesis is that local activation of P2X_7_R could become more widespread as neuronal degeneration progresses during AD.

Our findings in primary cultures show that in the presence of specific ligand, activation of microglial and astrocytic P2X_7_R triggers pro-inflammatory signalling. In cultured microglia, P2X_7_R activation induces the formation of NLRP3 inflammasome complexes and IL-1β release, in agreement with previous reports that P2X_7_R regulates the NLRP3 inflammasome (Mariathasan et al., 2006) and subsequent IL-1β maturation (Solle et al., 2001). Indeed, P2X_7_R antagonism reduces levels of IL-1β, and Aβ deposits in APP transgenic mice (Diaz-Hernandez et al., 2012). Similarly, P2X_7_R-mediated release of IL-1β by microglia is described in a mouse model of tauopathy (Di Lauro et al., 2022) with IL-1β believed to influence tau phosphorylation through actions on GSK-3 (di Lauro et al., 2022, Ghosh et al., 2013), although others suggest the involvement of different mechanisms (Carvalho et al., 2021, Ruan et al., 2020). Nevertheless, these data implicate P2X_7_R upstream of microglial NLRP3 inflammasome-driven progression of Aβ and tau pathology and accompanying cognitive deficits in mice (Heneka et al., 2013, Ising et al., 2019, Stancu et al., 2019, Venegas et al., 2017). Our data further supports that microglial P2X_7_R could contribute to elevations in IL-1β and IL-18, end-products of the NLRP3 inflammasome pathway, in AD brain and peripheral fluids (Brosseron et al., 2014, Ising et al., 2019, Griffin et al., 1989, Ojala et al., 2009). While the assembly of active NLRP3 inflammasomes in microglia is well established, the role of this complex in astrocytes is much less understood (Heneka et al., 2018). Here we found that NLRP3 was neither constitutively expressed nor induced upon treatment with BzATP in astrocytes, in agreement with other reports using different stimuli (Gustin et al., 2015). However, NLRP3 appears to be upregulated under specific conditions such as mechanical strain and exposure to Aβ_1-42_ species *in vitro* (Albalawi et al., 2017, Ebrahimi et al., 2018) suggesting a context-dependant regulation of this inflammasome component. Moreover, BzATP stimulation did not lead to the formation of ASC specks in astrocytes, which are required for NLRP3, as well as absent in melanoma 2 (AIM2), inflammasome signalling (Barclay et al., 2022, Dick et al., 2016). Hence, our study suggests that P2X_7_R activation mediates different downstream signalling cascades in primary cultured mouse astrocytes and microglia, at least under the conditions we examine here.

Consistent with this, in astrocytes we found that P2X_7_R affects NFκB translocation and production of CCL2, IL-6, CXCL1 and the acute-phase protein Lcn2, all of which are downstream of NFκB (Khorooshi et al., 2008, Lee et al., 2012b, Liu et al., 2022, Spooren et al., 2010, Zhao et al., 2020). NFκB is increased in AD brain (Kitamura et al., 1997) and pharmacological inhibition of NFκB attenuates astrocyte reactivity in APP/PS1 and 5xFAD mice (Lindsay et al., 2021). P2X_7_R is reported to modulate NFκB by interactions with adaptor protein myeloid differentiation primary-response protein 88 (MyD88) and recruitment of interleukin-1 receptor-associated kinases (IRAK) (Liu et al., 2022) or by inducing MAPK phosphorylation (Panenka et al., 2001) following calcium entry (Neary et al., 2003), and indirectly promoting the transcriptional activity of NFκB in astrocytes (Saha et al., 2020, Saha et al., 2007). The cytokines CCL2 and CXCL1 were found here to be upregulated in the BA9 region of AD brain in agreement with previous reports (Barroeta-Espar et al., 2019, Perez-Nievas et al., 2021, Sokolova et al., 2009) suggesting a potential involvement of P2X_7_R in the increased presence of these cytokines in AD. In mouse models, CCL2 activity is linked with Aβ and tau deposition, and cognitive decline (el Khoury et al., 2007, Gutierrez et al., 2019, Joly-Amado et al., 2020, Kiyota et al., 2013, Yamamoto et al., 2005), and interactions between astrocytic CXCL1 and its receptor CXCR2 mediate Aβ- induced tau modifications and synapse toxicity *in vitro* (Perez-Nievas et al., 2021, Xia and Hyman, 2002). However, despite our verification that changes in cytokine levels observed in astrocyte and microglia cultures are reflected in changes in AD brain, caution should be taken when extrapolating these *in vitro* data to *in vivo* models and human disease.

Besides alterations in cytokine production, this study provides the first evidence of a link between P2X_7_R activity and the acute phase protein Lcn2 in astrocytes. Lcn2 is secreted from astrocytes under AD-mimicking conditions (Dekens et al., 2020, Staurenghi et al., 2021) and acts as an autocrine mediator of reactive astrocyte responses (Lee et al., 2009) that causes synaptic toxicity, neuronal death and cognitive deficits (Kim et al., 2017, Lee et al., 2012a, Staurenghi et al., 2021). Lcn2 was recently found to exacerbate tau pathology in P301S tau mice, which show P2X_7_R expression in astrocytes, potentially by increasing cellular uptake of tau seeds (Mann et al., 2022). Our data may indicate an important regulatory role for astrocytic P2X_7_R in these Lcn2-driven neurodegenerative cascades, particularly those involving tau.

Indeed, our evaluation of the P2X_7_R antagonist Cp2 in an *ex vivo* model of tauopathy showed that P2X_7_R inhibition caused marked reductions in pathological tau inclusions, adding to a growing body of literature linking P2X_7_R with pathological tau changes in disease including tau misfolding (Ruan et al., 2020) and phosphorylation at specific epitopes (Carvalho et al., 2021, di Lauro et al., 2022). Here, we observed a specific effect on tau aggregation load rather than tau phosphorylation, at least at Ser396/404. The difference between our observations could arise from our models showing molecular diversity of mutated tau forms which show distinct features of spreading and folding (Dujardin et al., 2018) and can lead to different phenotypes over time, as well as variation in the pharmacological and genetic approaches employed to manipulate P2X_7_R. Although the precise contribution of microglial and astrocytic P2X_7_R to the reduction of tau aggregates in this *ex vivo* model remains to be addressed, we hypothesise that in addition to interactions between P2X_7_R-mediated inflammatory signals and tau, P2X_7_R could regulate non-cell autonomous clearance mechanisms governing protein homeostasis. There is evidence that P2X_7_R activation alters autophagy in glia (Kim et al., 2018, Takenouchi et al., 2009), contributes to a dysfunctional ubiquitin–proteasome system (Bianchi et al., 2023) and impairs phagocytosis (Di Lauro et al., 2022), key glial mechanisms known to influence tau pathology *in vivo* (Andersson et al., 2019, Luo et al., 2015, Martini-Stoica et al., 2018, Myeku et al., 2016). Intriguingly, an inverse association between P2X_7_R protein levels and phosphorylated soluble, but not insoluble, tau species was observed in AD BA9 brain. Although it is not straightforward to link this observation to our functional findings *ex vivo*, it should be noted that recent studies in AD brain have highlighted the heterogeneity and diversity of tau pathology across individuals (Dujardin et al., 2018) which is associated with complex patterns of post-translational modifications altering tau aggregation and propagation (Wesseling et al., 2020). Thus, future investigations focusing on distinct molecular tau features might uncover novel associations between P2X_7_R and human tauopathies.

### 4.1 Conclusions

Here we provide proof that P2X_7_R is elevated in AD brain prior to substantial synapse loss and neurodegeneration. We demonstrate that *P2RX7 mRNA* is expressed by both microglia and astrocytes, answering a key question in P2X_7_R biology. Our findings indicate that the expression of *P2RX7* in these different cell types could be important for understanding the activation of P2X_7_R in disease since P2X_7_R induction drives distinct prominent inflammatory signalling cascades in microglia (via NLRP3 inflammasome complexes) and astrocytes (via NFκB). In organotypic brain slice cultures expressing mutant human tau we determine that P2X_7_R antagonism reduces tau aggregate burden without influencing tau phosphorylation or synaptic localisation. Together, our data highlight a potential non-cell autonomous function of P2X_7_R in clearing tau aggregates and support future investigations to uncover specific astrocyte and microglia-driven consequences of P2X_7_R activation for tau-associated neurodegeneration.

## 5 Ethics approval and consent to participate

All donors had provided written informed consent for the use of post-mortem brain for research purposes to the London Neurodegenerative Disease Brain Bank. All human tissue collection and processing were carried out under the regulations and licensing of the Human Tissue Authority, and in accordance with the UK Human Tissue Act, 2004. All animal work was conducted in accordance with the UK Animals (Scientific Procedures) Act 1986 and the European Directive 2010/63/EU under UK Home Office Personal and Project Licenses and with agreement from the King’s College London (Denmark Hill) Animal Welfare and Ethical Review Board.

## 6 Consent for publication

Not applicable.

## 7 Availability of data and materials

Raw data and uncropped blots are included as supplementary data files.

## 8 Competing interests

The authors declare they have no financial competing interests. MSP is an employee of Astra Zeneca.

## Funding

This work was funded by Alzheimer’s Research UK (ARUK-PhD2018-002) to WN and BGP-N, Van Geest Charitable Foundation funding to BGP-N, Astra Zeneca (WPAM216014SW) to WN, DPH and MSP, a Medical Research Council Transition Award to MJ-S (MR/V036947/1), the National Health and Medical Research Council (APP1154692) to MK, a Race Against Dementia Alzheimer’s Research UK fellowship (ARUK-RADF2019A-003) to CLC, and the UK Dementia Research Institute (UK DRI-6005) to M−DR, where CLC is also based, which receives its funding from UK DRI Ltd, funded by the UK Medical Research Council, Alzheimer’s Society and Alzheimer’s Research UK. The London Neurodegenerative Disease Brain Bank receives funding from the Medical Research Council and the Brains for Dementia Research programme, jointly funded by Alzheimer’s Research UK and Alzheimer’s Society. This study was also supported by the National Institute for Health and Care Research Exeter Biomedical Research Centre. The views expressed are those of the author(s) and not necessarily those of the NIHR or the Department of Health and Social Care.

## 10 Authors’ contributions

WN, BGP-N and PB-L designed the study and WN and BGP-N supervised the research. PB-L planned and performed most experiments and analysed data with help from MMH, CT, DJ, M–DR and CLC with critical input from MJ-S, MSP, TEG, DPH and AV. All authors read, edited, and approved the final manuscript.

## Declaration of Competing Interest

The authors declare that they have no known competing financial interests or personal relationships that could have appeared to influence the work reported in this paper.

## Data Availability

Primary data included as supplementary
