# Supplementary material for: P2X7R influences tau aggregate burden in human tauopathies and shows distinct signalling in microglia and astrocytes
Source: Brain Behav Immun. 2023 Nov;114:414–29. doi: 10.1016/j.bbi.2023.09.011 (PMC10896738; doi:10.1016/j.bbi.2023.09.011)
Supplement: Supplementary data 1 [file mmc1.pdf]

Beltran Lobo et al. Supplementary methods and data

METHODS

Cloning

To generate the pcDNA3-P2X7-GSG15-FLAG expression vector, the coding region of $P2X_7R$ was amplified from pLV-EF1a-P2X7-TwinStrep-IRES-IFP using the primers Hind3_Kozak-$P2X_7R$_fwd and BamH1-$P2X_7R$_rev (Supplementary Table 2) and inserted into the Hind3 and BamH1 sites of pcDNA3-GSG15-FLAG (described by Reber et al. (2016)). To create the parental lentiviral plasmid pLV-EF1a-P2X7-TwinStrep-IRES-IFP, the coding region of human $P2X_7R$ followed by a C-terminal TwinStrep tag was ordered by gene synthesis (General Biosystems) and inserted into the BamH1 and Xma1 sites of pLV-EF1a-IRES-IFP.

HEK 293T cell culture

HEK 293T cells (Invitrogen) were cultured in DMEM/F12 (Gibco) supplemented with 10 % FBS, 100 U/mL penicillin and 100 µg/mL streptomycin in T175 flasks and maintained at 37 ºC and 5 % CO_2. At 90 % confluency, cells were trypsinised in 1X trypsin/EDTA (Gibco) and seeded into 6- or 12-well plate containing sterile coverslips at $5x10^5$ or $1,6x10^5$ cell/well. Cell culture medium was replaced by fresh DMEM/F12 24 h after seeding and prior to transfection.

Transfection of the human *P2RX7*-FLAG vector (Supplementary Figure 2) was performed using Lipofectamine 2000 (Invitrogen) according to the manufacturer's instructions. In brief, 1 µg or 300 ng DNA were used for 6-well and 12-well plates, respectively, and the corresponding amount of lipofectamine was diluted separately in Opti-MEM (Gibco) and incubated for 5 min at ambient temperature. The mixes were combined and incubated for 10 min allowing the DNA-lipid complexes to form and then homogeneously distributed on the cells in a dropwise manner. Cells were harvested or fixed 48 h post-transfection.

Anti-$P2X_7R$ antibody validation

HEK 293T cell lysates were immunoblotted as described in materials and methods and membranes were incubated with anti-FLAG (1:10000, Sigma-Aldrich, F3165) and the following primary antibodies anti-$P2X_7R$: Alomone (1:200, APR-004), Novus Biologicals (1:200, NBP1-37775), Proteintech (1:500, 28207-1-AP) or Thermo-Fischer (1:100, PA5-29274).

Fixed HEK 293T cells were immunolabelled as described in materials and methods and incubated with anti-FLAG (1:1000, Sigma-Aldrich, F3165) and the following primary antibodies anti-$P2X_7R$: Alomone (1:100, APR-004), Novus Biologicals (1:100, NBP1-37775), Proteintech

(1:100, 28207-1-AP) or Thermo-Fischer (1:100, PA5-29274) and mounted using fluorescent mounting media with DAPI (Invitrogen). Cells were imaged using a 60x oil lens in an epifluorescence Eclipse Ti-3 camera inverted microscope (Nikon) using the NIS-Elements AR software (Ver. 5.01). Images were stored as JPEG files and processed in Fiji (Schindelin et al., 2012) for adjustment of brightness and contrast maintaining the same LUTs across different samples of the same experiment.

Immunolabelling of human brain sections

Frontal cortex sections (7 µm) of human AD and control brain (BA9) were prepared from 10 % FFPE blocks by the MRC London Neurodegenerative Diseases Brain. Sections were deparaffinised by two consecutive washes in 100% (v/v) xylene and 99.9 % (v/v) ethanol for 5 min each at RT. Sections were then immersed in distilled H_2O for 5 min at RT. To reverse epitope masking, sections were submerged in antigen retrieval solution containing 0.01 M sodium citrate (pH 6.0), boiled at 95-100 ºC for 5 min and heated at 65 ºC for 12 min in a microwave. Non-specific binding was blocked with 1 % (v/v) normal donkey serum (Sigma, D9663) in TBS for 1 h at RT and sections were incubated with antibodies against Aβ (1:500, BioLegend, 803001) and GFAP (1:200, DAKO, Z0334) or Iba1 (1:100, WAKO, 019-19741) in 0.1 % donkey serum in TBS at 4 ºC overnight. Sections were washed three times in TBS and incubated with fluorophore-coupled secondary antibodies, AlexaFluor 568 (1:250, Invitrogen) and AlexaFluor 488 (1:250, Invitrogen) in 0.1 % donkey serum in TBS for 1 h at RT protected from light, followed by 3 washes in TBS. Sections were incubated with 0.3 % (w/v) Sudan black (Acros Organics) in 70 % (v/v) ethanol for 10 min in the dark followed by 8 washes in TBS. Tissue sections were then mounted using fluorescent mounting media with nuclear DAPI stain (Invitrogen) and imaged using an slide scanner VS120 microscope (Olympus).

Immunolabelling of organotypic brain slice cultures

Organotypic brain slices were stained according to an extended version of Gogolla et al. (2006). In brief, BSCs were washed with ice-cold PBS prior to fixation with 4 % PFA and after every step thereafter and incubated at 4 ºC overnight. BSCs were fixed with ice-cold 20 % (v/v) methanol in PBS at 4 ºC overnight prior to permeabilisation with 1 % (v/v) Triton X-100 in PBS at 4 ºC overnight. To prevent nonspecific binding, slices were incubated with blocking solution containing 20 % (w/v) BSA in PBS overnight at 4 ºC. All these steps were performed with three slices attached to an individual insert. Individual BSCs were then cut out of the membrane and transferred to a 12-well plate (1 slice/well) and incubated with an antibody against phosphorylated tau at Ser202/Thr205 (1:100, AT8, MN1020) diluted in 5 % (w/v) BSA in PBS at 4 ºC for 72 h. BSCs were washed three times with 5 % BSA in PBS prior to incubation

with AlexaFluor 568 conjugated secondary antibody (1:200, Invitrogen), in 5 % BSA in PBS at 4 ºC overnight in the dark. BSCs were washed in 5 % BSA in PBS and mounted using fluorescent mounting medium with DAPI (Invitrogen).

BSCs were imaged using a Nikon Eclipse Ti Inverted Spinning Disk Confocal microscope with a Yokogawa CSU-1 disk confocal head unit and Andor Ixon3 EMCCD camera with a 20x lens and imaged using the NIS-Elements AR Software. All parameters including laser settings and calibration were constant through the image capturing. Image "Z" stacks covering the total depth of the slice were taken. A maximum intensity projection was generated by collapsing the Z stacks using Fiji. Images were stored as JPEG files and processed in Fiji for adjustment of brightness and contrast maintaining the same LUTs across different samples of the same experiment.

SUPPLEMENTARY FIGURES

Fig S1.

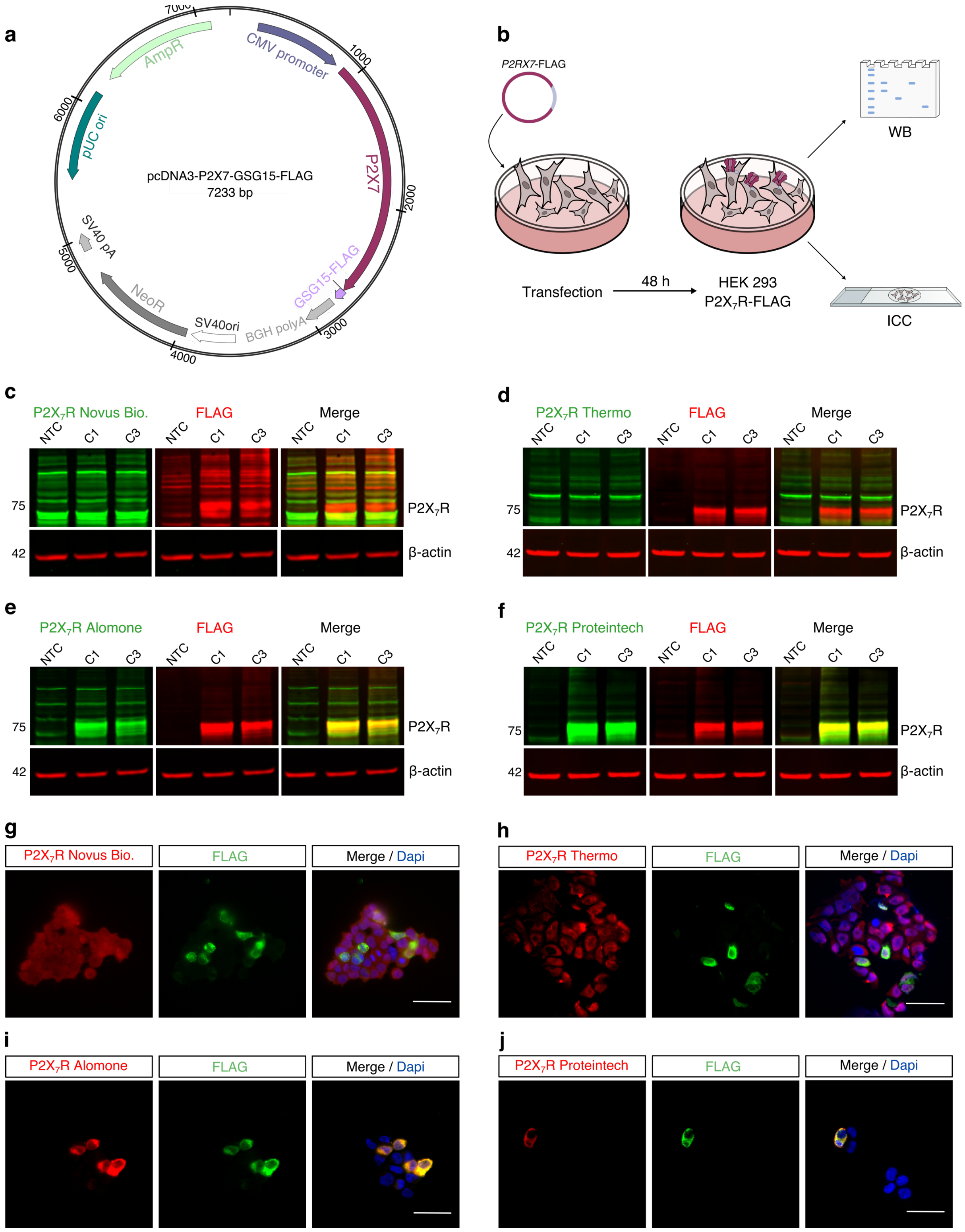

Supplementary Figure 1: Validation of commercial antibodies against $P2X_7R$. a) Plasmid map of the pcDNA3-P2X7-GSG15-FLAG construct used to overexpress human $P2X_7R$ tagged

with a FLAG peptide in HEK 293 cells. **b)** Diagram of the experimental workflow used to validate $P2X_7R$ antibodies: Alomone (APR-004), Novus Biologicals (NBP1-37774), referred to as Novus Bio, Proteintech (28207-1-AP) and Thermo-Fisher (PA5-29274), referred to as Thermo. HEK 293 cells were transfected with the $P2X_7R$-FLAG construct for 48 h and harvested or fixed for WB or ICC, respectively. Representative immunoblots are lysates from non-transfected controls (NTC) or HEK 293 cells following transfection with the $P2X_7R$ clones (C1 and C3). Lysates were immunoblotted using different antibodies against $P2X_7R$ (green) as follows: **(c)** Novus Bio, **(d)**, Thermo, (**e**), Alomone **(f)** Proteintech. All membranes were also immunoblotted with an antibody against FLAG (red). Representative immunofluorescence images of HEK 293 cells expressing $P2X_7R$-$FLAG^+$ and non-transfected ($FLAG^-$) cells from the same coverslip. Fixed cells were immunolabelled with antibodies against $P2X_7R$ (red) as follows: **(g)** Novus Bio, **(h)** Thermo, (**i**) Alomone and **(j)** Proteintech. DAPI was used to stain the nuclei (blue). Scale bar: 50 µm.

Fig S2.

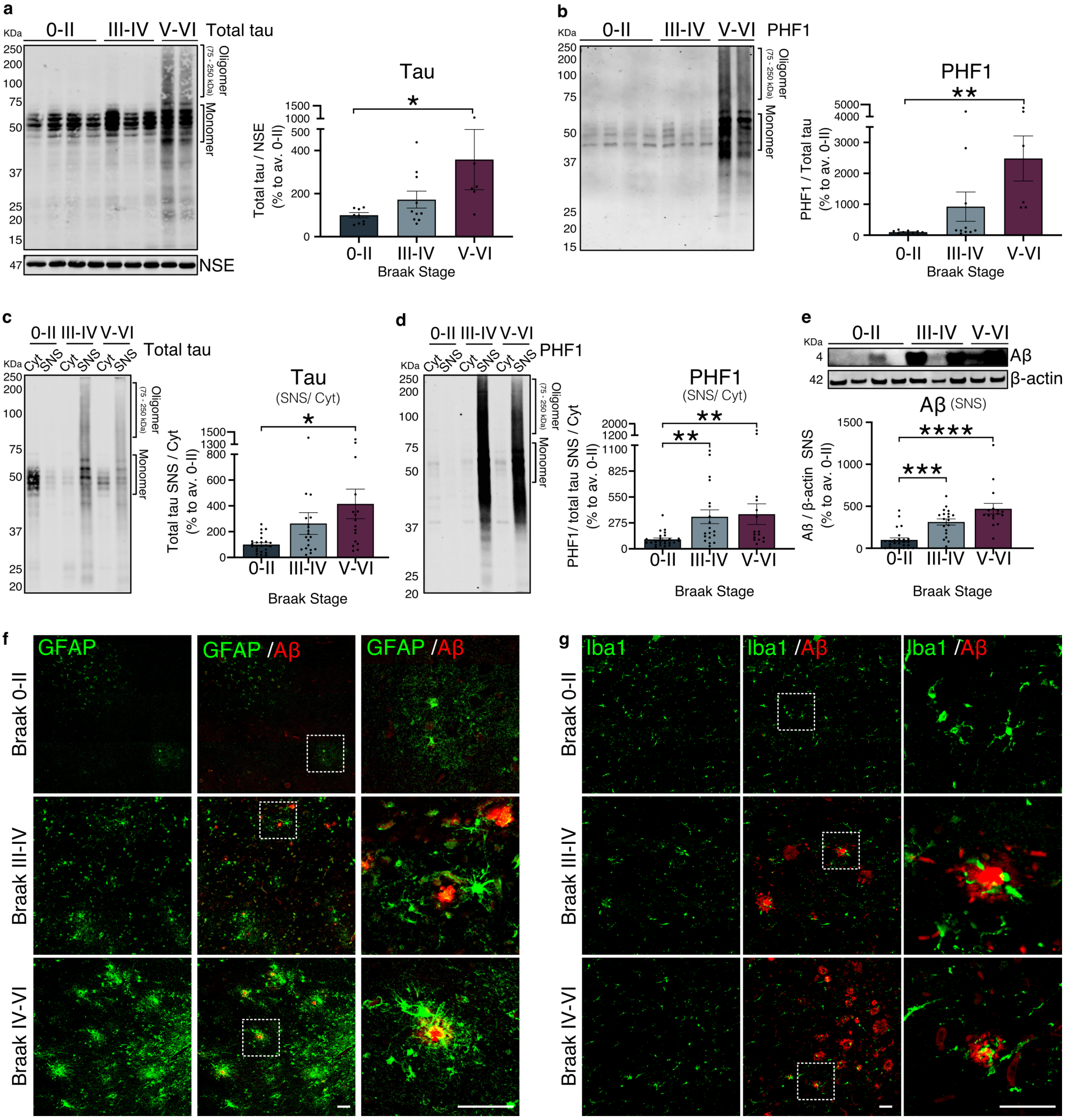

Supplementary Fig. 2. Tau and Aβ alongside GFAP^{+}-astrocytes and Iba1^{+}-microglia in BA9. Representative immunoblots of **(a)** total tau and neuronal specific enolase (NSE) and **(b)** tau phosphorylated at Ser396/404 (PHF1) in BA9 total homogenates. Bar charts show quantification of **(a)** total tau relative to NSE and **(b)** PHF1 relative to total tau in each sample as % of average control (Braak 0-II). n= 10 (0-II), 10 (III-IV), 6 (V-VI). **(c-e)** Representative immunoblots of cytosolic (cyt) and synaptoneurosome (SNS) fractions of BA9 prefrontal cortex immunoblotted with antibodies against **(c)** total and **(d)** tau phosphorylated at Ser396/404 (PHF1) and **e)** Aβ (6E10) and β-actin. Bar charts display the ratio of **(c)** total tau and **(d)**

phosphorylated tau normalised to total tau, in the SNS relative to the cytosolic fraction and **e)** Aβ relative to β-actin in SNS expressed as % of average control (Braak 0-II) n= 25 (0-II), 19 (III-IV), 16 (V-VI). Values are mean ± SEM. Following d'Agostino and Pearson normality test, data was analysed using Kruskal-Wallis test and Dunn´s multiple comparison test. *$p<0.05$, **$p<0.01$, ***$p<0.001$, ****$p<0.0001$. Representative images of prefrontal cortex sections from control (0-II), moderate (III-IV) and severe (V-VI) cases immunolabelled with antibodies against **(f)** GFAP or **(g)** Iba1 (green) and Aβ (6E10, red). Insets indicate areas proximal to Aβ plaques and are displayed at higher magnification. Scale bars: 50 µm. n=5 per group.

Fig S3.

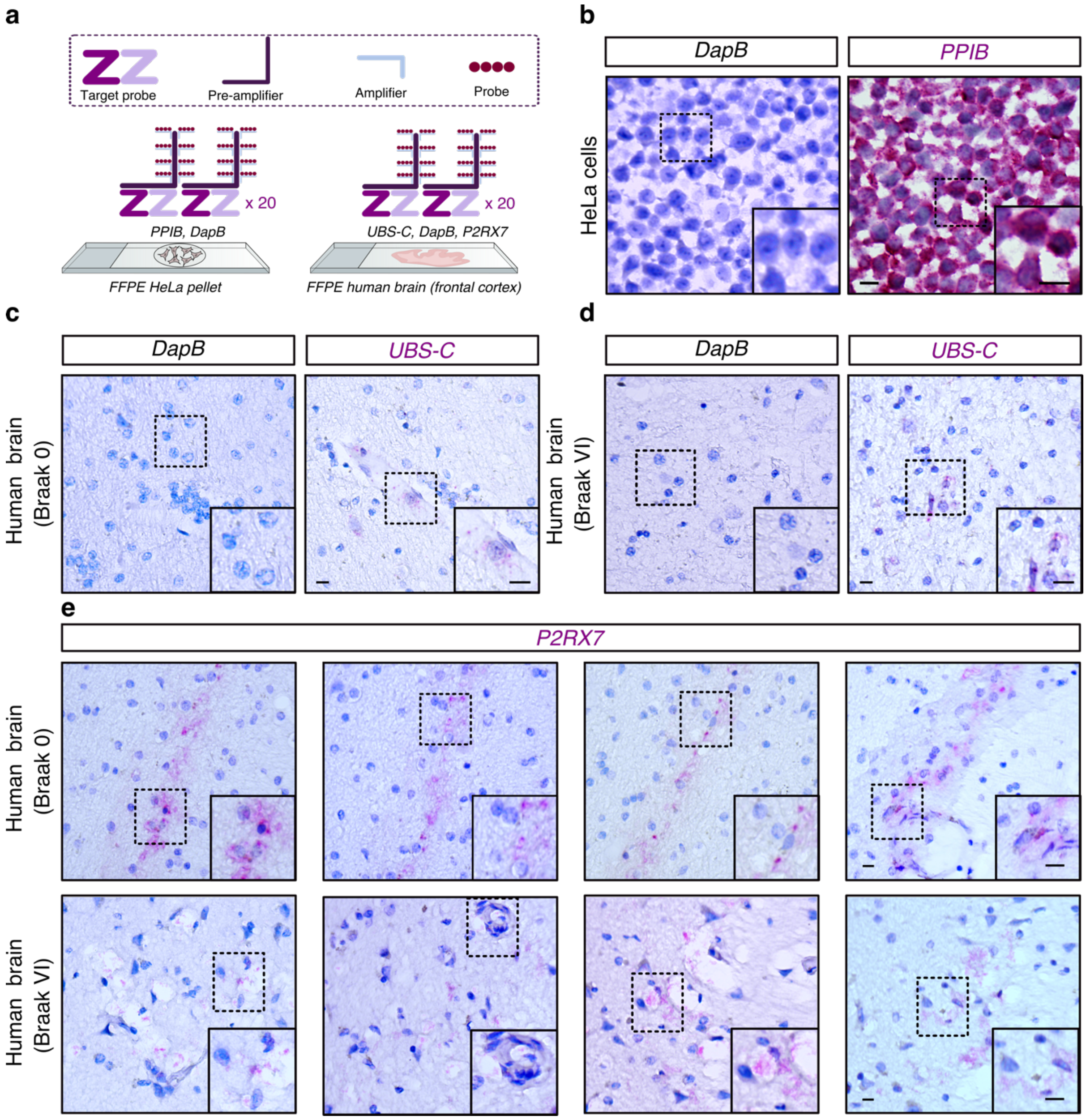

Supplementary Fig. 3. Validation of the RNAscope protocol and detection of *P2RX7* mRNA in human brain. a) Schematic diagram depicting the RNAscope components required for target hybridisation including ZZ target probes, complementary pre-amplified sequence, amplifier and red chromogenic probes. This system was used to target *PPIB* (positive control), UBS-C (positive control) and *DapB* (negative control) mRNA in commercially available FFPE HeLa cells and human control (Braak 0) and AD (Braak VI) FFPE prefrontal cortex sections. **b)** Representative images of HeLA cell pellets hybridised against *DapB* (left) and *PPIB* (right) (n=1 per probe). Representative images of FFPE prefrontal cortex (BA9) from **(c)** control and

(d) AD brain hybridised with probes against *DapB* (left) and *UBS-C* (right). **e)** Representative images of control (upper panel) and AD (lower panel) brain sections hybridised with probes against *P2RX7*. Magnified areas are indicated with a black rectangle. Haematoxylin was used as a counterstain. Scale bar: 10 µm. **(c-e)** n=3 per group, control (Braak 0) and AD (Braak VI).

Fig. S4

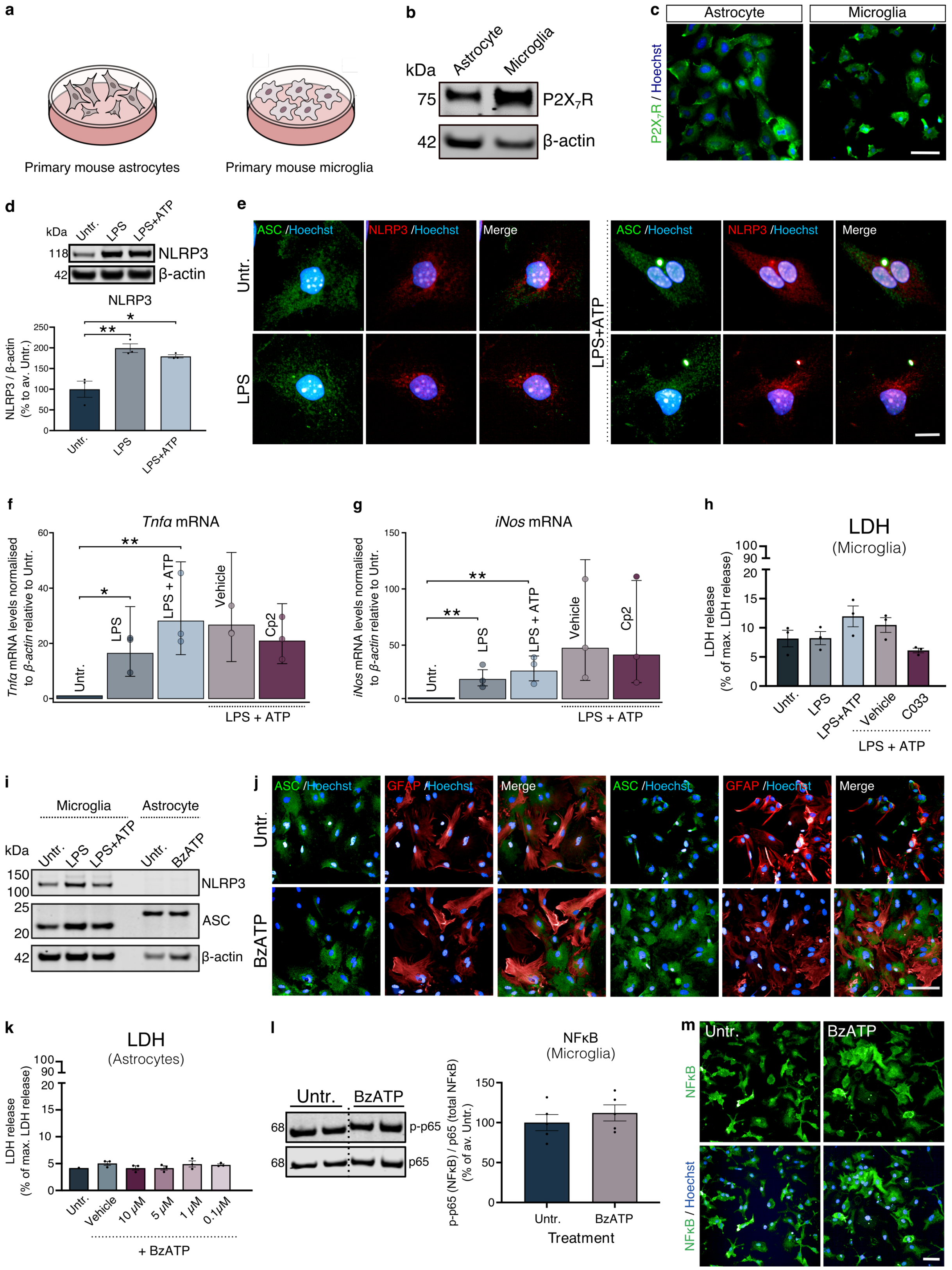

Supplementary Fig. 4. Signalling downstream of $P2X_7R$ in mouse microglia and astrocytes. a) Diagram of mouse primary astrocyte and microglia cultures. **b)** Representative immunoblot of astrocyte and microglia lysates immunoblotted with an antibody against $P2X_7R$

(Alomone). β-actin was used as loading control. **c)** Representative immunofluorescence images of $P2X_7R$ labelling (Alomone) in fixed astrocytes and microglia. Hoescht-33342 was used to stain nuclei. Scale bar: 50 µm. **(d-e)** Microglia cultures untreated (Untr.), primed with 100 ng/mL LPS (3 h) or stimulated with 1 mM ATP (20 min) following LPS-priming (100ng/mL, 3 h). **d)** Representative immunoblot of microglial lysates immunoblotted with an antibody against NLRP3 (1:1000, Adipogen, AG-20B-0014). β-actin was used as loading control. Bar graph shows quantification of NLRP3 normalised to β-actin expressed as a percentage of untreated. **e)** Representative images of microglia immunolabelled with antibodies against ASC (green) and NLRP3 (red, 1:100). Hoechst-33342 was used as a nuclear stain. Scale bar: 10 µm. **f-h)** Microglia cultures untreated (untr.), primed with LPS (100 ng/mL, 3 h), stimulated with 100 ng/mL LPS (3 h) and 1 mM ATP (20 min) and pre-treated with 0.1% DMSO (vehicle) or 1 µM Cp2 for 1 h prior to the addition of ATP in LPS-primed cells. Quantitative mRNA analysis shows the relative abundance of **f)** *Tnfα* and **g)** *iNos* mRNAs normalised to β-actin relative to untreated (n=3). **h)** LDH levels in the supernatant of microglial cultures. Values are the proportion of LDH released into the cell culture medium relative to total LDH amounts released by lysed microglia in each independent experiment, expressed as a percentage of total LDH (n=3). **i**) Microglia cultures untreated (untr.), primed with 100 ng/mL LPS (3 h), LPS (100 ng/mL, 3 h) and stimulated with 1 mM ATP (20 min). Astrocyte cultures treated with BzATP (300 µM, 4h) or untreated (untr.). Representative immunoblots of microglia and astrocyte lysates immunoblotted with antibodies against NLRP3 and ASC. β-actin was used as loading control (n=3). **j)** Representative images of astrocytes untreated (untr.) or BzATP-stimulated (300 µM, 4 h), immunolabelled with antibodies against ASC (green) and GFAP (red, 1:500, Antibodies.com, A85307). Scale bar: 100 µm. **k)** LDH levels in the supernatant of astrocyte cultures under basal conditions (untr), pretreated with 0.1% DMSO (vehicle) or Cp2 at the concentrations indicated for 1 h prior to the addition of BzATP (300 µM, 4 h). Values are the proportion of LDH released into the cell culture medium relative to total LDH amounts released by lysed astrocytes in each independent experiment, expressed as a percentage of total LDH (n=3). **l-m)** Microglia cultures were untreated (untr.) or stimulated with BzATP (300 µM, 4h) Representative immunoblots of microglial lysates probed with antibodies against NFκB (p-p65) phosphorylated at Ser536 and total NFκB (p65 subunit). Bar chart displays quantification of p-NFκB normalised to total NFκB for each sample, expressed as a percentage relative to untreated cells (n=5). **m)** Representative images of microglia immunolabelled with an antibody against NFκB (p65 subunit, green). Hoechst-33342 was used as a nuclear stain. Scale bar: 50 µm (n=3). Values are mean ± **(d,h, k,l)** SEM or **(f,g)** SD. Following Shapiro-Wilk normality tests, data was analysed using a **(d, h, k)** one-way ANOVA test with d) Tukey or (**h,k**) Dunnett´s multiple comparison test, a **(f,g)** Welch´s or **(l)** unpaired t-test.

Fig S5.

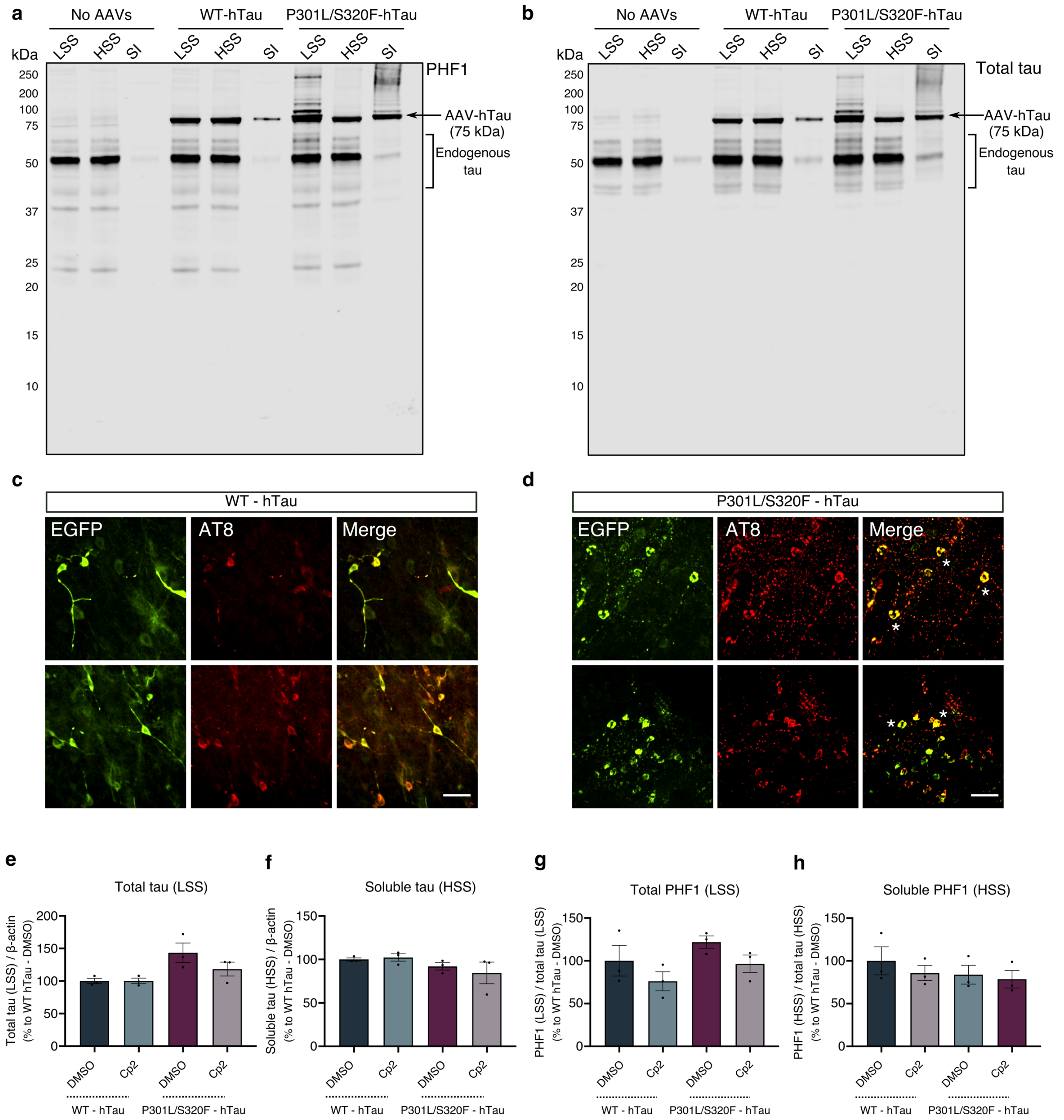

Supplementary Fig. 5. P2X$_7$R antagonism does not affect phosphorylated (PHF1) or total tau levels in total (LSS) and soluble tau fractions (HSS) in BSCs. a-b) Representative immunoblots of BSCs under basal conditions (no AAVs), transduced with WT-hTau-EGFP or P301L/S320F hTau-EGFP at 1x10^{11} vg/mL at 0 DIV and harvested at 28 DIV probed with antibodies against **(a)** tau phosphorylated at Ser396/404 (PHF1) and **(b)** total tau. **(c-d)** Representative confocal images of BSCs transduced with EGFP-tagged WT (left) or P301L/S320F (right) hTau and immunolabelled with an antibody against tau phosphorylated at Ser202/Thr205 (AT8) at 28 DIV. Asterisks indicate dense somatic phosphorylated tau

inclusions. Scale bar: 50 µm. **(e-h)** BSCs transduced with WT or P301L/S320F hTau. Region-matched areas treated with 0.1% DMSO or Cp2 from 14 DIV and harvested at 28 DIV. Bar graphs display the quantification of total tau levels normalised to β-actin **(e)** in LSS (sarkosyl-soluble and insoluble tau) and **(f)** HSS (sarkosyl-soluble) fractions and PHF1 normalised to total tau in **(g)** LSS and **(h)** HSS fractions expressed as a percentage of the control condition (BSCs transduced with WT-hTau and treated with 0.1% DMSO). **(e-h)** Following Shapiro-Wilk normality test, data was analysed using two-way ANOVAs with Sidak´s multiple comparison test (n=3). Two-way ANOVA indicates **(e)** hTau construct ($F_{(1,8)}$ =10.0; p=0.01), drug treatment ($F_{(1,8)}$=1.64, p=0.23), drug treatment x hTau construct ($F_{(1,8)}$=1.68, p=0.23), **(f)** drug treatment ($F_{(1,8)}$=0.14, p=0.71), hTau construct ($F_{(1,8)}$=3.45, p=0.1), drug treatment x hTau construct ($F_{(1,8)}$=0.49, p=0.5), **(g)** drug treatment ($F_{(1,8)}$=4.01, p=0.08), hTau construct ($F_{(1,8)}$=2.95, p=0.12), drug treatment x hTau construct ($F_{(1,8)}$=0.00, p=0.96), **(h)** drug treatment ($F_{(1,8)}$=0.67, p=0.44), hTau construct ($F_{(1,8)}$=0.94, p=0.36), drug treatment x hTau construct ($F_{(1,8)}$=0.14, p=0.72).

Fig. S6

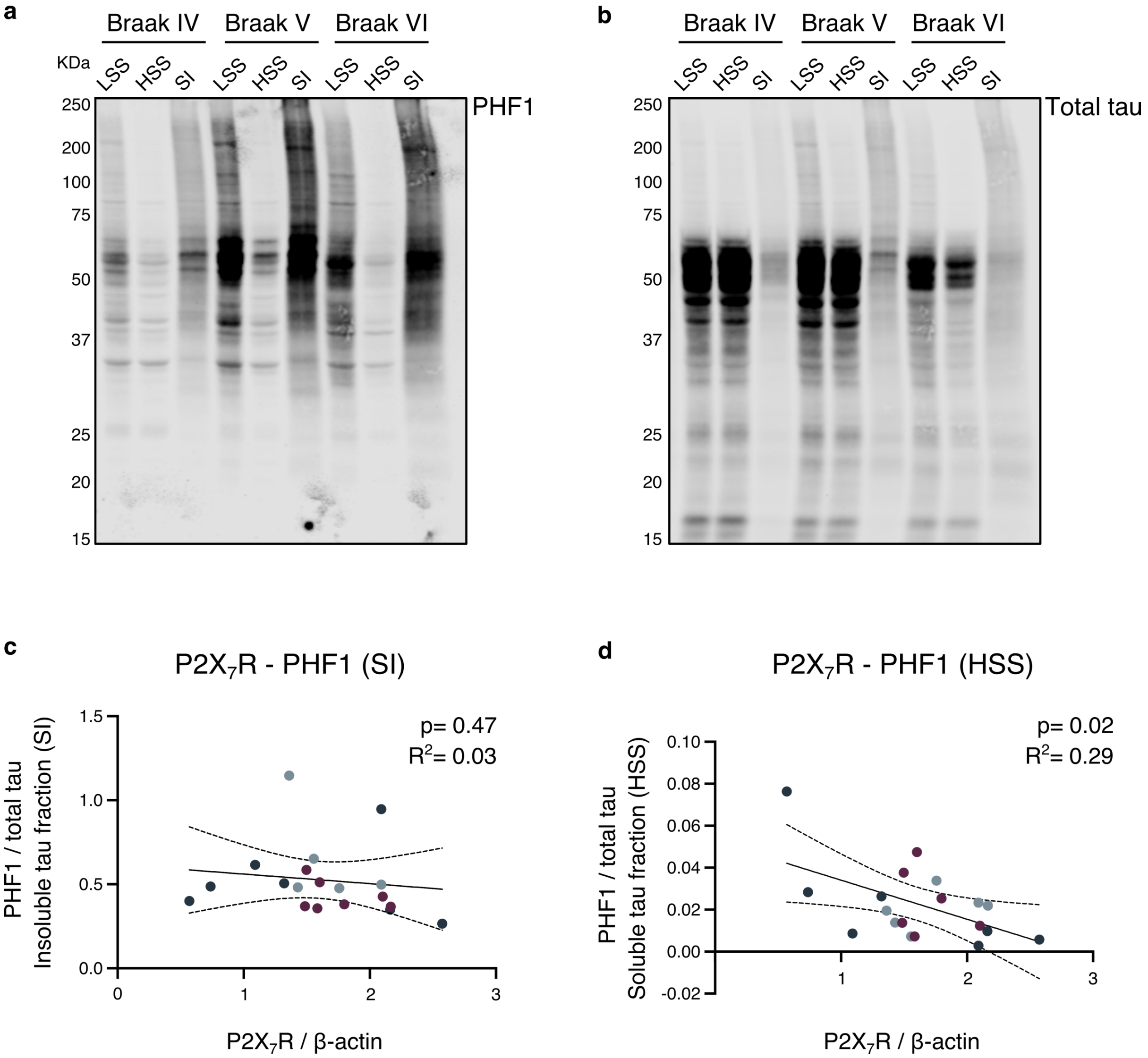

Supplementary Fig. 6. Increased P2X7R levels correlate with a reduction in soluble but not insoluble phosphorylated tau (PHF1) in BA9 AD brain. Representative immunoblots of low-speed supernatant (LSS, soluble + insoluble tau), high-speed supernatant (HSS, soluble tau) and sarkosyl-insoluble (SI, insoluble tau) of BA9 AD brain (Braak IV-VI) immunoblotted with antibodies against **(a)** tau phosphorylated at Ser396/404 (PHF1) and **(b)** total tau. Bar graphs show the correlation analysis of the amounts of P2X7R normalised to β-actin and phosphorylated (PHF1) normalised to total tau in the **(c)** SI and **(d)** HSS fractions (n=19). Colours represent Braak IV (dark blue), Braak V (light blue) and Braak VI (purple) AD samples.

.
