# Supplementary material for: P2X7R influences tau aggregate burden in human tauopathies and shows distinct signalling in microglia and astrocytes
Source: Brain Behav Immun. 2023 Nov;114:414–29. doi: 10.1016/j.bbi.2023.09.011 (PMC10896738; doi:10.1016/j.bbi.2023.09.011)
Supplement: Supplementary data 2 [file mmc2.pdf]

Beltran-Lobo et al. Supplementary Tables

Table S1.

**Sex**	**Age** (y)	**PMD** (h)	**Braak Stage**	**Pathology/Diagnosis**	**Brain Area**	**Use**
M	40	40	0	Control adult brain.	BA9	WB<br>CA
M	90	45	0/I	Control. Mild age-related changes. Braak stage + with mild focal amyloid angiopathy.	BA9	WB
F	87	22	0/I	Control adult brain.	BA9	WB<br>IF
M	78	10	0/I	Control. Ageing changes (mild).	BA9	WB<br>CA<br>RNA<br>IF
					BA21	WB
F	77	21	I	Control adult brain.	BA9	WB
M	82	47	I	Control case. Very early Alzheimer disease pathology – BNE stage I) with focal amyloid angiopathy.	BA9	WB
F	73	27	I	Control. Early ageing changes BNE stage I.	BA9	WB
F	92	17	I	Control brain with some tau deposition.	BA9	WB
F	55	12	I	Control. Minimal tau pathology consistent with HP-tau stage I.	BA9	WB<br>CA
F	81	17	I	Control. Mild ageing changes Braak I.	BA9	WB<br>CA
					BA21	WB
M	84	53	I/II	Control. Control case but with hypoxic-type changes and amyloid angiopathy.	BA9	WB
M	80	48.25	I/II	Control. Normal adult brain consistent with ageing.	BA9	WB
M	81	18	I/II	Control. Old cerebral infarct.	BA9	WB
F	89	41	I/II	Control with mild focal amyloid angiopathy.	BA9	WB

Sex	Age (y)	PMD (h)	Braak Stage	Pathology/Diagnosis	Brain Area	Use
M	86	6	I/II	Control adult brain.	BA9	WB<br>RNA
M	59	50	I/II	Control adult brain.	BA9	WB
F	78	6	I/II	Control. Primary Age related tauopathy (PART) BNE stage 1-2. Mild small vessel disease.	BA9	RNA
F	82	13	II	Control. Argyrophilic grains low to moderate density.	BA9	WB<br>CA
M	82	24	II	Control. Old infarcts in the right cerebral hemisphere.	BA9	WB
F	92	9	II	Control adult case.	BA9	WB
M	79	47	II	Early tau pathology, Braak II, no neuritic plaques.	BA9	WB<br>CA<br>IF
F	84	35	II	Alzheimer changes Braak II consistent with ageing.	BA9	WB<br>CA<br>IF
F	97	39	II	Very mild Alzheimer's disease type changes BNE stage 2, control.	BA9	WB<br>CA
F	86	45	II	Alzheimer's type changes (ageing), BNE stage 2, control.	BA9	WB<br>CA
M	93	33	II	Mild Alzheimer's-type changes Braak II.	BA9	WB<br>CA<br>IF
					BA21	WB
F	90	50	II	Control brain, mild Alzheimer-type changes and mild amyloid angiopathy.	BA21	WB

Sex	Age (y)	PMD (h)	Braak Stage	Pathology/Diagnosis	Brain Area	Use
M	81	12.5	III	Alzheimer changes consistent with Braak III and amyloid angiopathy.	BA9	WB
F	92	19.5	III	Alzheimer's disease (moderate – limbic stage) Braak III. Cerebral infarcts.	BA9	WB
F	98	3.5	III	Alzheimer's disease BNE IV, CERAD probable: TDP-43 pathology (hippocampus and amygdala).	BA9	WB
						CA
F	89	36.5	III	Alzheimer's disease pathology (BNE stage IV) with amyloid angiopathy and limbic TDP-43 pathology but cognitively not significantly impaired.	BA9	WB
						CA
M	86	52	III	Ageing changes (AD modified Braak III).	BA9	WB
						CA
						IF
					BA21	WB
F	70	38	III	Possible AD (CERAD), Braak III (limbic), BNE stage III.	BA9	WB
						CA
M	92	11	III	Mild Alzheimer-type changes Braak III.	BA9	WB
						CA
						RNA
					BA21	WB
M	88	79	III/IV	Alzheimer's Disease (modified Braak (BNE) stage III-IV. Mild amyloid angiopathy.	BA9	WB
M	84	86	IV	Alzheimer's disease (modified Braak (BNE) stage IV) with moderate amyloid angiopathy.	BA9	WB
						CRR

Sex	Age (y)	PMD (h)	Braak Stage	Pathology/Diagnosis	Brain Area	Use
F	95	47	IV	Alzheimer's disease (modified Braak (BNE) stage IV) with moderate amyloid angiopathy and moderate to severe cerebrovascular changes and limbic TDP-43 pathology (Joseph stage II).	BA9	WB<br>CRR
F	86	55.5	IV	Alzheimer's disease (modified Braak (BNE) stage IV) with mild amyloid angiopathy.	BA9	WB<br>CRR
M	91	48	IV	Alzheimer's disease pathology (modified Braak (BNE) stage IV), Thal phase IV with moderate to severe amyloid angiopathy.	BA9	WB
M	98	53	IV	Alzheimer's Disease (modified Braak (BNE) stage IV, Mild amyloid angiopathy.<br>Mild limbic TDP-43 proteinopathy, watershed infarcts.	BA9	WB<br>CRR
F	89	56	IV	AD HP tau stage 4, severely affecting limbic system and moderately extending to neocortex.	BA9	WB<br>CA<br>IF
M	92	70	IV	Alzheimer's disease (BNE stage 4).	BA9	WB<br>CA<br>CRR
M	82	28	IV	Alzheimer's disease (modified Braak stage IV) with limbic TDP-43 pathology.<br>Widespread severe amyloid angiopathy; diffuse microvascular cerebrovascular pathology.	BA9	WB<br>CA<br>RNA<br>IF
					BA21	WB
F	92	29.5	IV	Alzheimer's disease Braak IV.<br>Tau positive grain disease.	BA9	WB
F	83	22	IV	AD (limbic stage-modified Braak IV) with moderate-severe amyloid angiopathy.	BA9	WB<br>CA<br>RNA<br>CRR<br>IF
					BA21	WB

Sex	Age (y)	PMD (h)	Braak Stage	Pathology/Diagnosis	Brain Area	Use
M	86	53	IV	AD (modified Braak IV) with extensive severe amyloid angiopathy.	BA9	WB<br>CA<br>CRR<br>IF
					BA21	WB
M	83	77	V	Alzheimer’s disease BNE modified Braak stage V with extensive amyloid angiopathy.<br>mild to moderate small vessel disease in frontal and occipital white matter.	BA9	WB<br>CRR
M	86	26	V	AD Braak V with moderate amyloid angiopathy.	BA9	WB<br>CA<br>CRR
F	84	24	V	Alzheimer’s disease Braak V.	BA9	WB<br>CA<br>CRR<br>IF
F	97	12	V	Alzheimer’s disease Braak V.	BA9	WB<br>CA
F	80	13	V	AD Braak V with mild amyloid angiopathy.	BA9	WB<br>CA<br>RNA<br>CRR
					BA21	WB
F	82	69	V	AD Braak V with mild amyloid angiopathy.	BA9	WB<br>CA<br>CRR<br>IF
					BA21	WB
M	73	35	V/VI	Alzheimer’s disease Braak VI BNE 5.	BA9	WB
F	83	26.5	VI	Alzheimer’s disease Braak VI	BA9	WB<br>CRR
M	72	5.5	VI	Alzheimer’s disease Braak VI with marked amyloid angiopathy.	BA9	WB<br>RNA

Sex	Age (y)	PMD (h)	Braak Stage	Pathology/Diagnosis	Brain Area	Use
F	95	48	VI	Alzheimer's disease (modified Braak stage VI, Thal phase V with mild amyloid angiopathy and limbic stage. Dementia with Lewy bodies and limbic TDP pathology.	BA9	WB<br>CRR
M	85	27	VI	Alzheimer's disease Braak VI, Thal phase V and moderate degree of cerebrovascular pathology and limbic and early cortical TDP-43 pathology.	BA9	WB
F	89	38.5	VI	Alzheimer's disease (modified Braak (BNE) stage VI) with moderate amyloid angiopathy and dementia with Lewy bodies (limbic stage) and limbic TDP-43 pathology.	BA9	WB<br>CRR
M	81	74	VI	AD Braak VI.	BA9	WB<br>CA<br>CRR
F	87	48	VI	AD Braak VI with moderate amyloid angiopathy.	BA9	WB<br>CA
M	72	5	VI	AD Braak VI with marked amyloid angiopathy.	BA9	WB<br>CA<br>CRR<br>IF
F	91	28.5	VI	Alzheimer's disease Braak VI.	BA9	WB<br>CA<br>CRR<br>IF
F	69	16	VI	Alzheimer's disease Braak VI.	BA21	WB
F	84	36	VI	Alzheimer's disease Braak VI.	BA21	WB
M	80	41	VI	Alzheimer's disease Braak VI with severe amyloid angiopathy.	BA9	WB<br>CA<br>CRR<br>IF
					BA21	WB
F	89	6	VI	Alzheimer's disease Braak VI.	BA9	RNA

Supplementary Table 1. Characteristics of human post-mortem samples.

Age is shown in years (y), post-mortem delay (PMD) in hours (h), sex, pathology/diagnosis and Braak stage. BA9 and BA21 tissues used for protein quantification, mRNA detection and correlation analysis are indicated as follows: western blotting (WB), cytokine array (CA), RNAscope (RNA), correlation analysis of $P2X_7R$ and tau species (CRR) and immunofluorescence (IF).

Table S2.

ID	5' – sequence – 3'	Length
**Cloning primers**		
Hind3-Kozak-P2X$_7$R_fwd	CCCAAGCTTGCCGCCACCATGCCCGCCTGCTGCAGCTG	38
BamH1-P2X$_7$R_rev	TCTGGATCCATAAGGGCTCTTGAAGCCGG	29
**qPCR primers**		
β-actin_fwd	CTATTGGCAACGAGCGGTTC	20
β-actin_rev	GCACTGTGTTGGCATAGAGG	20
CCL2_fwd	ACCTGCTGCTACTCATTCAC	20
CCL2_rev	TCTGGACCCATTCCTTCTTG	20
CXCL1_fwd	AATGAGCTGCGCTGTCAGTG	20
CXCL1_fwd	AAGCCTCGCGACCATTCTTG	20
IL-6_fwd	AGACCTGTCTATACCACTTC	20
IL-6_rev	CAAGTGCATCATCGTTGTTC	20
iNOS_fwd	CAAGATGGCCTGGAGGAATG	20
iNOS_rev	TACAGTTCCGAGCGTCAAAG	20
Lcn2_fwd	CAGAAGGCAGCTTTACGATG	20
Lcn2_rev	CCTGGAGCTTGGAACAAATG	20
TNFα_fwd	CCACGCTCTTCTGTCTACTG	20
TNFα_rev	GAGGGAGGCCATTTGGGAAC	20

Supplementary Table 2: Oligonucleotide sequences for human P2X$_7$R and mouse β-actin, CCL2, CXCL1, IL-6, iNOS, Lcn2 and TNFα.

Table S3.

Gene ID	Gene name	TCX All median	Control-AD Log2FC	Control-AD p-adj	Control-PSP Log2FC	Control-PSP p-adj
ENSG00000175591	*P2RY2*	1.53	**1.15**	**0.000016**	0.64	0.13570
ENSG00000089041	*P2RX7*	6.07	**0.86**	**0.000595**	0.52	0.17648
ENSG00000099957	*P2RX6*	3.21	**-0.55**	**0.001336**	-0.35	0.17555
ENSG00000169860	*P2RY1*	3.20	**0.95**	**0.001682**	0.58	0.22489
ENSG00000083454	*P2RX5*	3.44	**-0.55**	**0.003448**	0.05	0.87464
ENSG00000171631	*P2RY6*	0.39	**0.82**	**0.007523**	-0.15	0.77820
ENSG00000174944	*P2RY14*	2.98	**0.65**	**0.008008**	0.01	0.98585
ENSG00000135124	*P2RX4*	2.24	**-0.20**	**0.032758**	-0.38	0.05142
ENSG00000182162	*P2RY8*	-2.44	0.67	0.063860	-0.24	0.73925
ENSG00000169313	*P2RY12*	4.88	0.43	0.267798	-0.21	0.77158
ENSG00000244165	*P2RY11*	5.80	-0.08	0.280261	0.05	0.72551
ENSG00000186912	*P2RY4*	-1.68	0.37	0.284159	0.06	0.93768
ENSG00000078589	*P2RY10*	-3.18	-0.19	0.498200	-0.35	0.38611
ENSG00000181631	*P2RY13*	2.98	0.06	0.850081	-0.45	0.30850

Supplementary Table 3. *P2RX* and *P2RY* family mRNA levels in temporal cortex (TCX) from AD and PSP brain relative to matched controls. Data from the Mayo RNA-seq cohort (AMP-AD consortium) (Allen et al., 2016). Fold-change (FC), adjusted p-value (p-adj).

Table S4.

Gene ID	Gene name		3M CRND8 vs WT		6M CRND8 vs WT		12M CRND8 vs WT		20M CRND8 vs WT	
		avg FPKM	log2FC	p-adj	log2FC	p-adj	log2FC	p-adj	log2FC	p-adj
ENSMUSG00000020787	*P2rx1*	0.15	0.17	1.00	-0.26	0.30	0.22	0.50	0.29	0.44
ENSMUSG00000029503	*P2rx2*	0.07	0.44	1.00	-0.21	NA	0.86	NA	-0.56	0.35
ENSMUSG00000027071	*P2rx3*	0.48	0.08	1.00	0.12	0.45	-0.18	0.34	0.02	0.96
ENSMUSG00000029470	*P2rx4*	4.35	-0.06	1.00	0.14	0.03	0.09	0.22	0.10	0.21
ENSMUSG00000005950	*P2rx5*	0.89	0.01	1.00	-0.19	0.24	0.00	1.00	0.02	0.96
ENSMUSG00000022758	*P2rx6*	1.59	0.17	0.68	0.02	0.85	0.03	0.83	0.11	0.34
ENSMUSG00000029468	*P2rx7*	1.87	0.00	1.00	-0.05	0.68	**0.21**	**2.85E-02**	**3.77E-01**	**8.75E-06**
ENSMUSG00000027765	*P2ry1*	1.20	-0.05	1.00	-0.18	0.28	0.20	0.30	0.29	0.13
ENSMUSG00000050921	*P2ry10*	0.02	0.04	1.00	-0.08	NA	0.35	NA	-0.15	0.94
ENSMUSG00000036353	*P2ry12*	12.46	-0.03	1.00	0.09	0.65	0.29	0.09	0.60	4.40E-05
ENSMUSG00000036362	*P2ry13*	3.98	-0.04	1.00	0.19	0.22	**0.62**	**2.09E-06**	0.83	6.39E-11
ENSMUSG00000036381	*P2ry14*	1.08	-0.03	1.00	-0.28	0.03	-0.10	0.60	0.15	0.49
ENSMUSG00000032860	*P2ry2*	0.17	0.03	1.00	0.31	0.21	0.44	0.07	**0.88**	**1.72E-06**
ENSMUSG00000044359	*P2ry4*	0.01	-0.39	1.00	-0.46	NA	0.60	NA	0.06	0.97
ENSMUSG00000048779	*P2ry6*	1.35	0.22	1.00	**0.60**	**6.78E-05**	**0.56**	**6.37E-05**	**0.92**	**1.46E-12**

Supplementary Table 4. *P2rx* and *Pry* family mRNA levels in forebrain from CRND8 APP transgenic mice relative to age-matched littermate wild-type mice. Data from the AMP-AD Knowledge Portal (doi: 10.7303/syn3157182) (McFarland et al., 2021). Fold-change (FC), adjusted p-value (p-adj).

Table S5.

Human Cytokine Array<br>Human brain (BA9, prefrontal cortex)								
**Cytokines/Acute phase proteins**	**Control (0-II)**		**Moderate AD (III-IV)**			**Severe AD (V-VI)**		
**Statistics**	**Mean**	**±SEM**	**Mean**	**±SEM**	**p-value**	**Mean**	**±SEM**	**p-value**
**CCL1**	100	15.17	274.9	87.00	0.14	330.0	77.91	0.04 (*)
**ICAM-1**	100	28.33	158.2	28.24	0.15	348.8	79.95	0.005 (**)
**IL-10**	100	16.09	252.2	78.77	0.12	381.0	72.35	0.007 (**)
**G-CSF**	100	28.17	252.1	82.60	0.12	347.4	69.25	0.02 (*)
**GM-CSF**	100	20.73	229.4	67.96	0.11	275.5	63.06	0.07
**IL-1α**	100	25.52	242.1	80.31	0.14	326.6	76.40	0.04 (*)
**IL-1RA**	100	29.04	219.1	69.70	0.25	285.3	64.54	0.13
**IL-2**	100	24.30	243.3	77.63	0.05	324.5	65.42	0.004 (**)
**IL-4**	100	19.99	199.8	61.63	0.15	308.3	60.21	0.008 (**)
**IL-5**	100	23.78	211.0	69.69	0.31	276.1	67.98	0.07
**IL-8**	100	20.20	190.2	57.32	0.28	289.0	62.52	0.03 (*)
**IL-12p70**	100	22.82	245.5	73.97	0.07	339.7	70.39	0.004 (**)
**IL-13**	100	28.58	242.8	80.40	0.17	291.8	67.94	0.11
**IL-16**	100	26.00	208.4	64.14	0.29	298.1	69.34	0.09
**IL-17A**	100	27.24	227.1	77.38	0.12	292.7	59.86	0.02 (*)
**IL-17E**	100	29.10	247.3	81.53	0.21	321.6	77.79	0.12
**IL-18**	100	26.68	276.5	91.64	0.09	363.9	73.50	0.02 (*)
**IL-21**	100	28.19	251.2	80.97	0.15	320.5	74.77	0.06
**IL-27**	100	23.49	267.7	94.10	0.26	407.3	91.18	0.03 (*)
**IL-32a**	100	29.29	261.0	87.08	0.20	356.3	89.54	0.05
**MIF**	100	29.83	226.6	70.82	0.22	265.9	60.03	0.13
**PAI-1**	100	36.22	204.7	61.87	0.08	237.0	47.96	0.05
**TNFα**	100	24.83	221.9	69.11	0.15	245.0	51.95	0.07
**TREM-1**	100	23.27	211.4	64.55	0.23	277.9	58.05	0.04 (*)

**IFN-γ**	100	20.33	208.1	59.22	0.18	274.3	54.16	0.02 (*)
**RANTES**	100	25.28	192.8	63.05	0.27	230.3	54.39	0.15
**MIP**	100	29.76	232.1	78.65	0.19	290.0	64.44	0.08
**CD40L**	100	25.03	242.7	84.47	0.23	283.8	61.41	0.09
**C5a**	100	21.07	194.8	62.08	0.45	248.1	51.40	0.05
**CXCL10**	100	20.60	229.7	66.70	0.04 (*)	289.2	58.24	0.02 (*)
**CXCL11**	100	23.05	241.3	78.89	0.08	327.1	66.74	0.01 (**)
**CXCL12**	100	18.58	205.9	53.92	0.15	319.7	66.85	0.02 (*)

Supplementary Table 5: Human cytokines and acute phase proteins in BA9 AD (Braak III-IV, V-VI) and control brain (Braak 0-II). Data is mean ± SEM expressed relative to control (Braak 0-II). n=10 per group (Braak stage 0-II, III-IV, V-VI). Following Shapiro-Wilk normality test, data was analysed using a non-parametric Kruskal-Wallis test with Dunn’s multiple comparison test.

Table S6.

Gene ID	Gene name		2.5M rTg4510 vs WT		4.5M rTg4510 vs WT		6M rTg4510 vs WT	
		avg FPKM	log2FC	p-adj	log2FC	p-adj	log2FC	p-adj
ENSMUSG00000020787	*P2rx1*	0.15	0.17	NA	-0.52	0.347352	-0.21	0.69517
ENSMUSG00000029503	*P2rx2*	0.05	0.16	NA	0.30	NA	0.70	NA
ENSMUSG00000027071	*P2rx3*	0.45	0.13	0.73	-0.22	0.515357	-0.05	0.88673
ENSMUSG00000029470	*P2rx4*	2.09	0.08	0.65	0.12	0.446813	0.17	0.14940
ENSMUSG00000005950	*P2rx5*	0.72	0.16	0.66	-0.06	0.940312	-0.09	0.75242
ENSMUSG00000022758	*P2rx6*	1.73	0.05	0.78	0.04	0.900985	0.10	0.45236
ENSMUSG00000029468	*P2rx7*	1.65	0.20	0.17	**0.25**	**0.034468**	0.40	**0.00003**
ENSMUSG00000027765	*P2ry1*	1.75	-0.06	0.79	0.15	0.427773	0.04	0.83276
ENSMUSG00000050921	*P2ry10*	0.01	0.43	NA	0.40	NA	0.22	NA
ENSMUSG00000036353	*P2ry12*	13.10	0.07	0.73	0.02	0.969342	0.36	0.00051
ENSMUSG00000036362	*P2ry13*	4.65	0.18	0.42	0.03	0.963902	0.27	0.08557
ENSMUSG00000036381	*P2ry14*	0.30	-0.02	0.97	0.02	0.986379	-0.17	0.63915
ENSMUSG00000032860	*P2ry2*	0.15	0.62	NA	0.66	0.056467	0.97	0.00085
ENSMUSG00000044359	*P2ry4*	0.01	0.03	NA	0.29	NA	0.43	NA

ENSMUSG00000048779	*P2ry6*	0.93	0.32	0.29	**0.91**	**0.000001**	**0.83**	**0.00001**

Supplementary Table 6. *P2rx* and *P2ry* family mRNA levels in forebrain from rTg4510 MAPT transgenic mice relative to age-matched littermate wild-type mice. Data from the AMP-AD Knowledge Portal (doi: 10.7303/syn3157182). Fold-change (FC), adjusted p-value (p-adj).
