# Supplementary material for: P2X7R influences tau aggregate burden in human tauopathies and shows distinct signalling in microglia and astrocytes
Source: Brain Behav Immun. 2023 Nov;114:414–29. doi: 10.1016/j.bbi.2023.09.011 (PMC10896738; doi:10.1016/j.bbi.2023.09.011)

$P2X_7R$ influences tau aggregate burden in human tauopathies and shows distinct signalling in microglia and astrocytes

Western blot membranes

Fig. 1a-b. Immunobloted with anti-$P2X_7R$ (Alomone, APR-004, 1:200) and ß-actin (abcam, Ab8226, 1:500)

Fig. 1a BA9 prefrontal cortex. Membrane was cropped and then incubated with primary antibody. The purple square indicates a severe AD case remove from the study (not quantified) because it did not match the neuropathological requirements (low Aß, and phosphorylated tau).

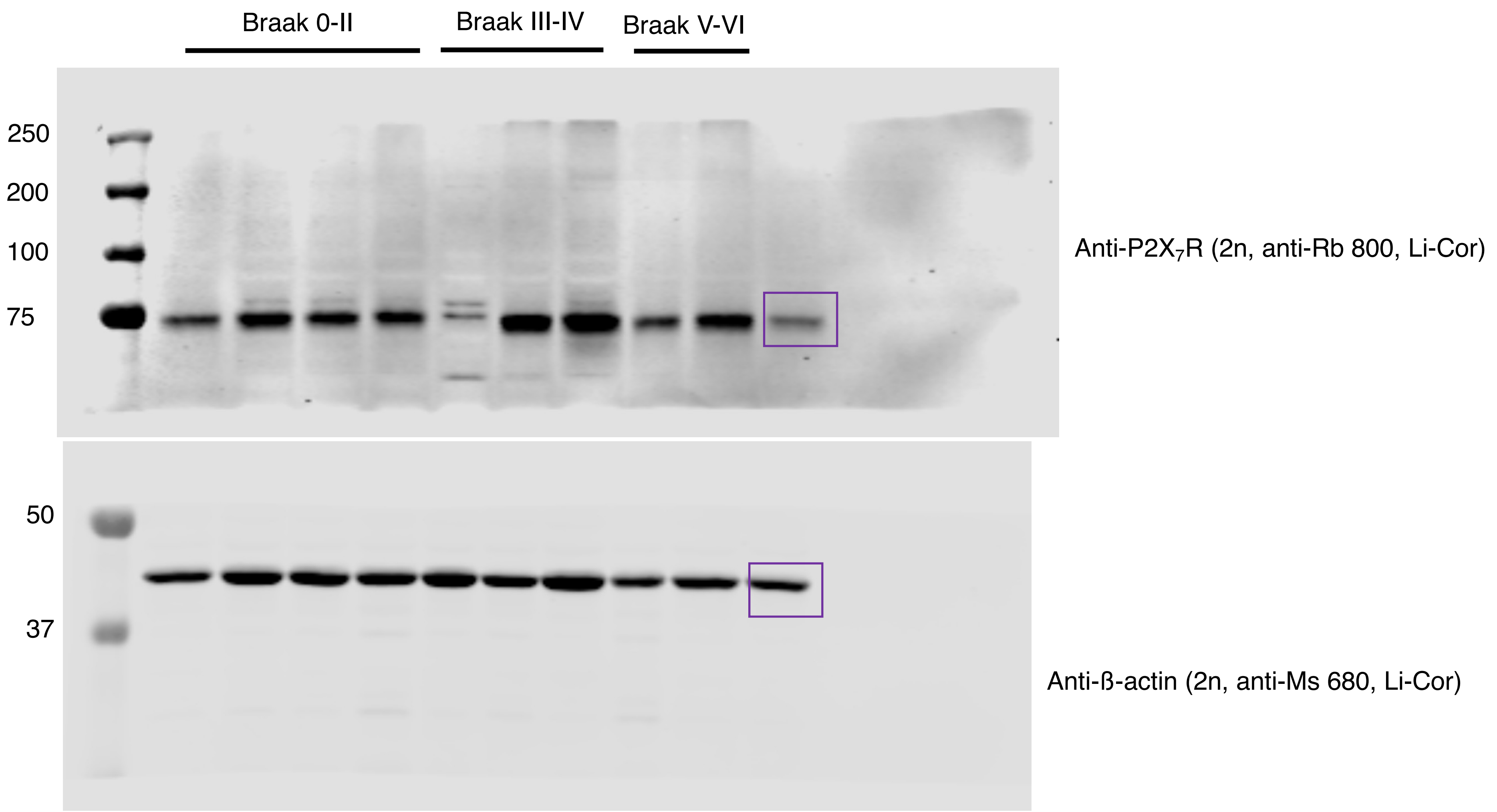

Fig. 1a-b. Immunobloted with anti-$P2X_7R$ (Alomone, APR-004, 1:200) and ß-actin (abcam, Ab8226, 1:500)

Fig. 1b BA21 temporal cortex. Membrane was cropped and then incubated with primary antibody.

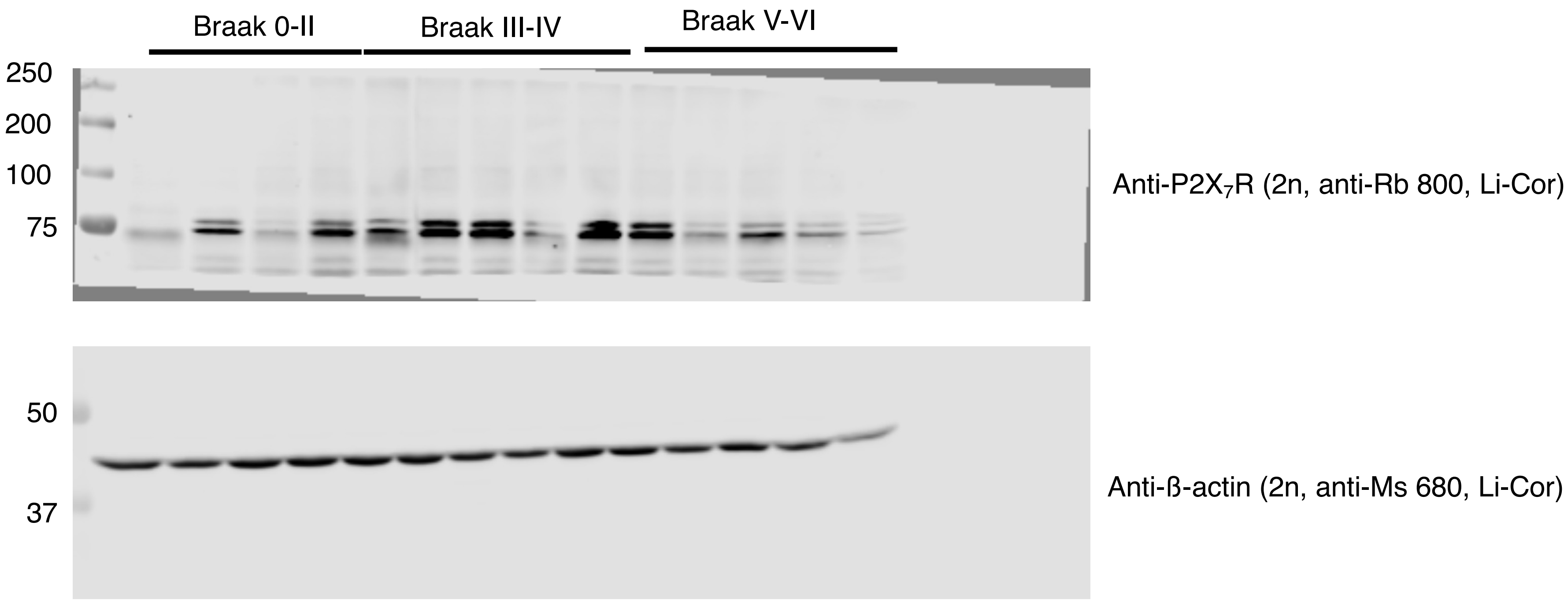

Fig. 1c-d. BA9 total homogenates immunobloted with antibodies against PSD-95 (Cell signalling, 3450, 1:1000) and NSE (1:10000, DAKO, M0873) in this order. The purple rectangle indicates a case quantified but not included in the main figure.

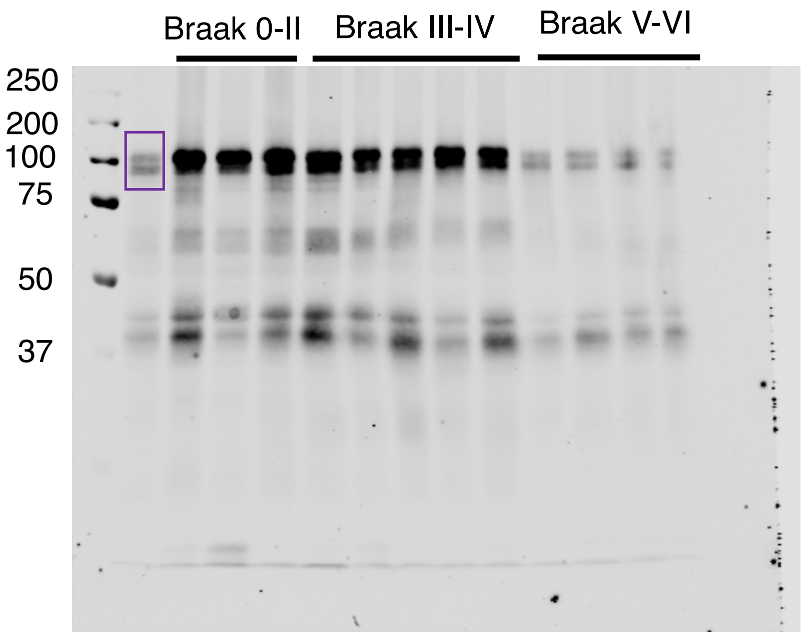

1) Anti-PSD95 (2n, anti-Rb 800, Li-Cor)

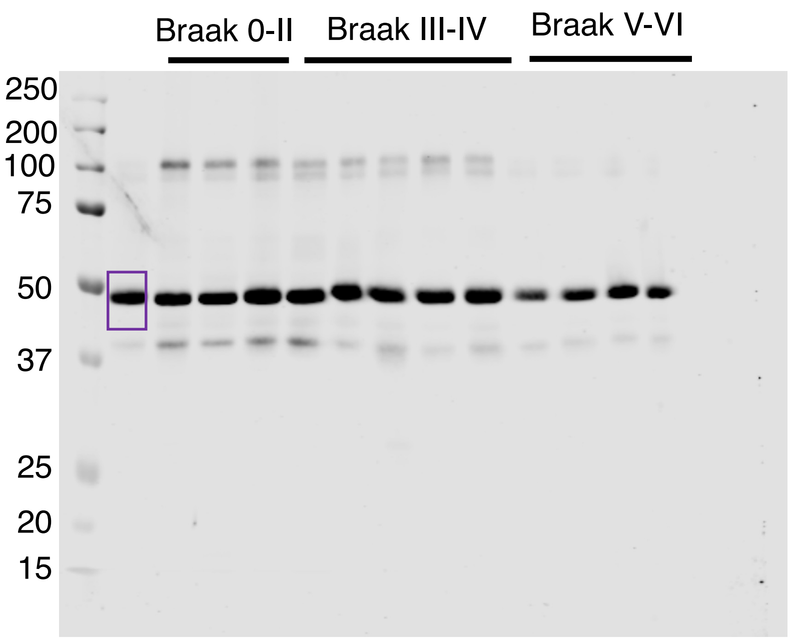

2) Anti-NSE (2n, anti-Ms 680, Li-Cor)

Fig. 1d. BA9 total homogenates immunobloted with antibodies against Synaptophysin or SYP (Santa Cruz, Sc-17750, 1:1000), NSE (1:10000, DAKO, M0873). The purple rectangle indicates a case quantified but not included in the main figure.

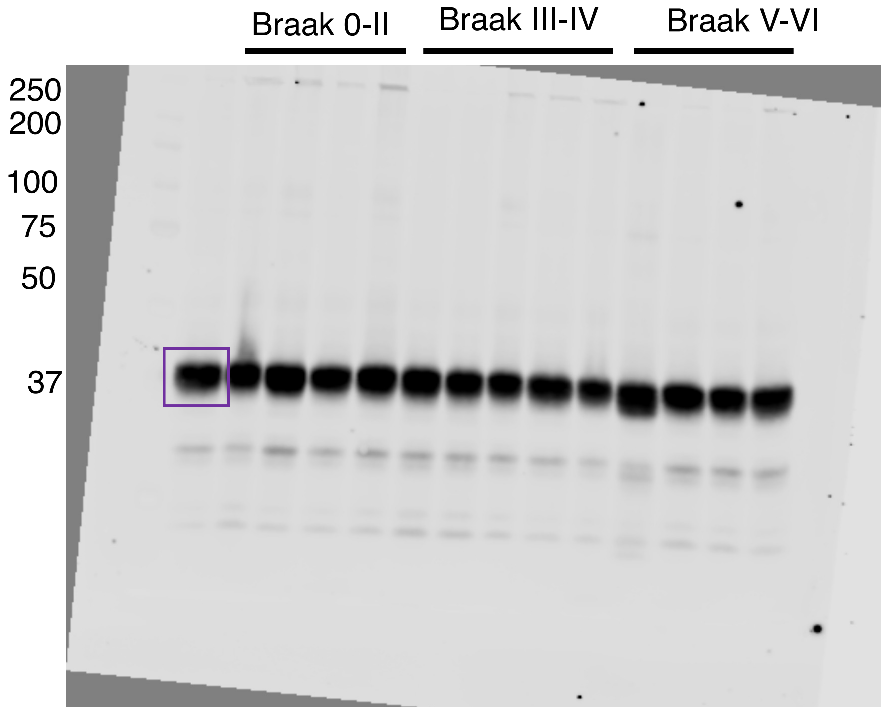

1) Anti-SYP (2n, anti-Ms 800, Li-Cor)

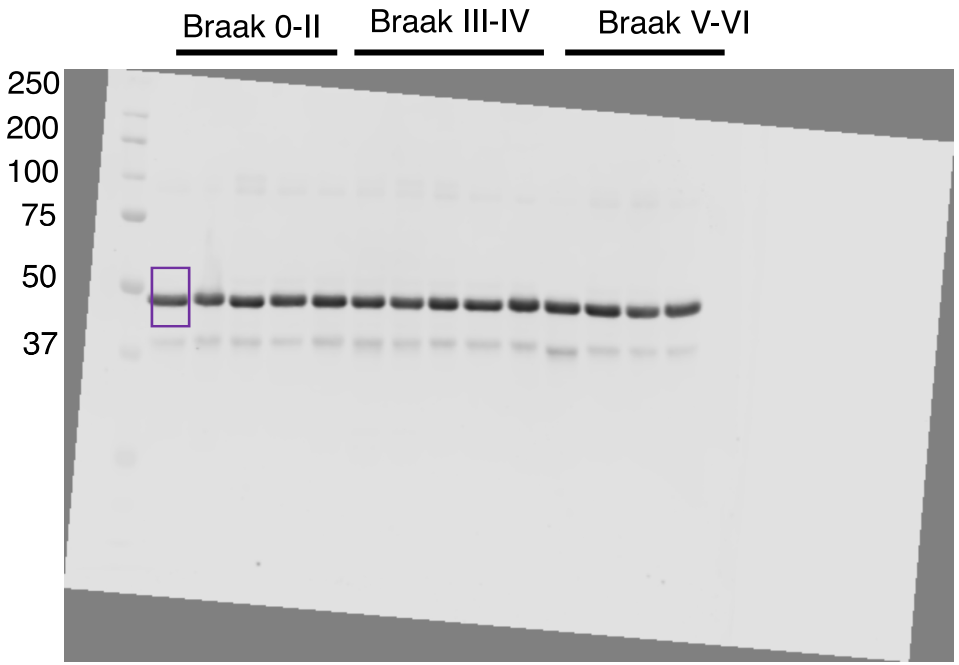

2) Anti-NSE (2n, anti-Ms 680, Li-Cor)

Fig. 1e. BA21 total homogenates immunobloted with antibodies against PSD-95 (Cell signalling, 3450, 1:1000), NSE (1:10000, DAKO, M0873).

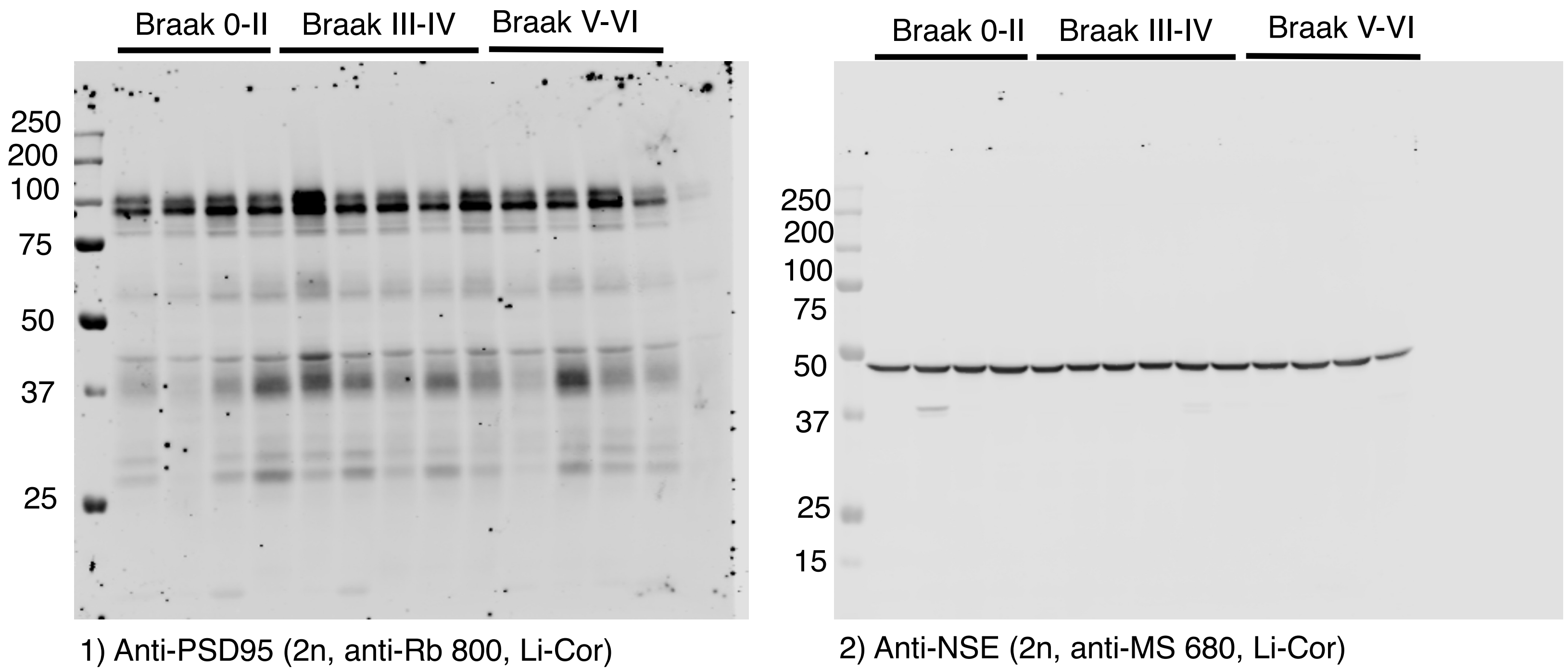

Fig. 1f. BA21 total homogenates immunobloted with antibodies against Synaptophysin or SYP (Santa Cruz, Sc-17750, 1:1000), NSE (1:10000, DAKO, M0873).

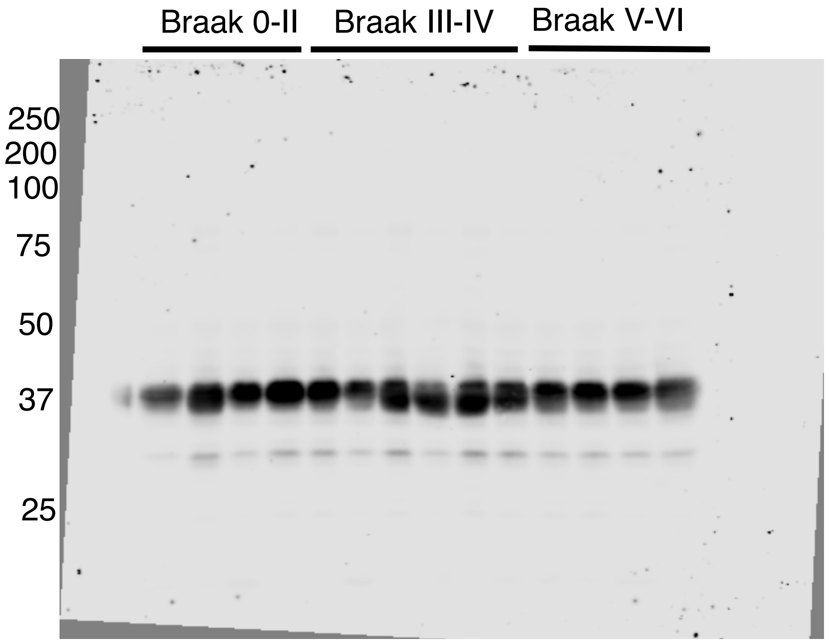

1) Anti-SYP (2n, anti-Ms 800, Li-Cor)

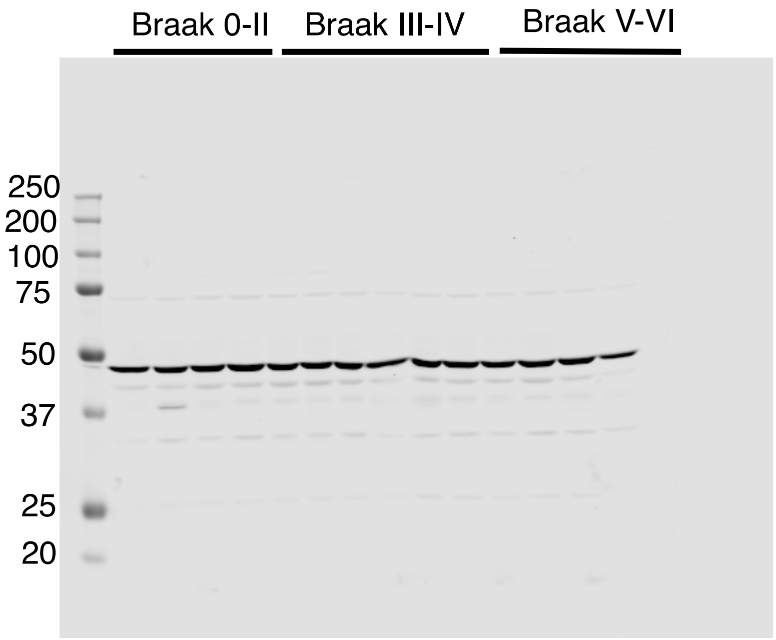

2) Anti-NSE (2n, anti-Ms 680, Li-Cor)

Fig. 4b Astrocyte lysates immunoblotted with antibodies against Lcn2 (R&D systems, AF1857, 1:500) and Aldh1L1 (1:100, UCDavid/NIH NeuroMab facility, N103/39). Membrane was cut prior to incubation with primary antibodies. Purple rectangles indicate cell treatments that were not included in this study (e.g. treatment with Aß oligomeric species) whereas the green rectangle indicates the ones that are part of the main figure.

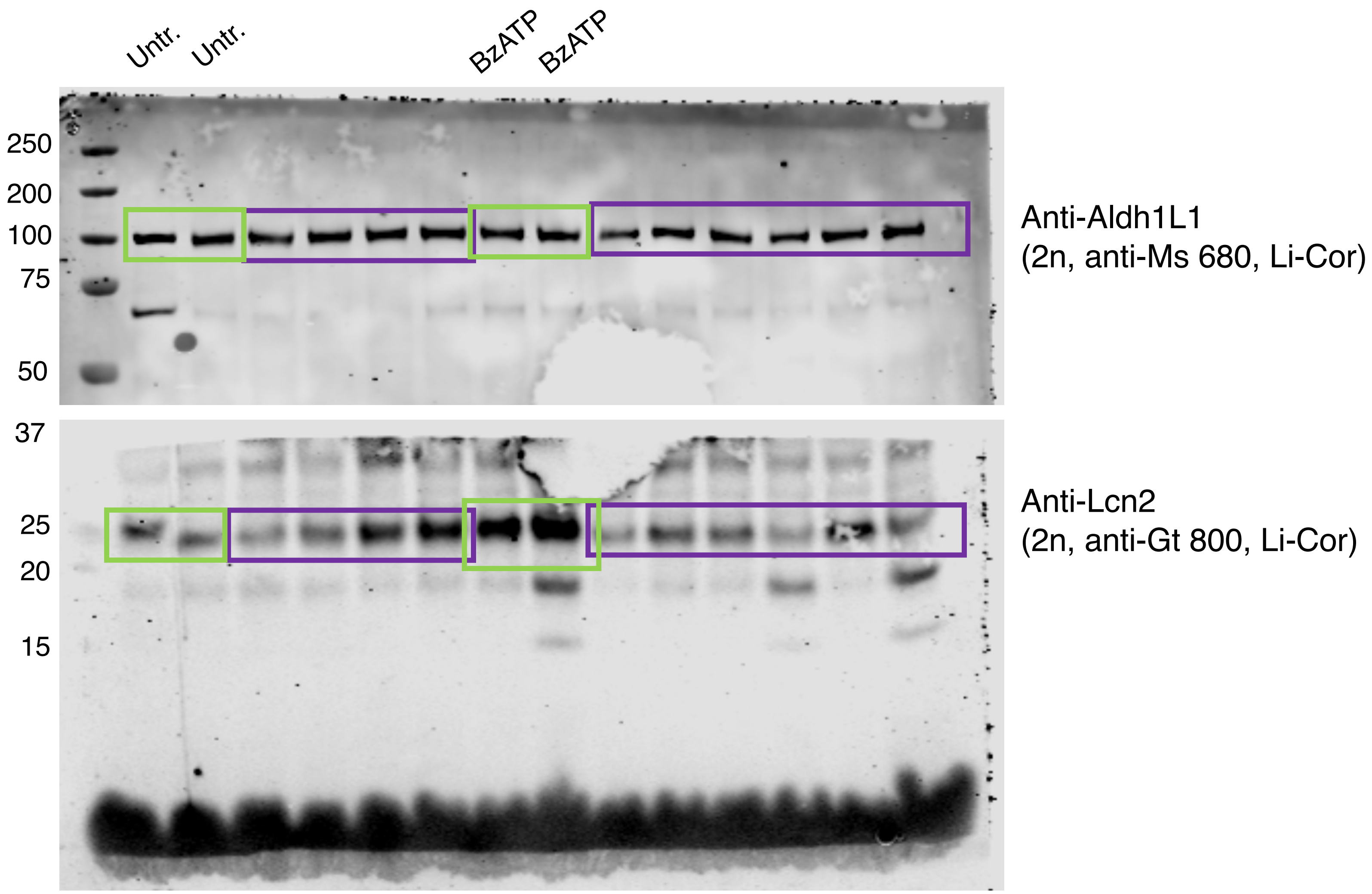

Fig. 4d Astrocyte lysates immunoblotted with antibodies against Lcn2 (R&D systems, AF1857, 1:500) and Aldh1L1 (1:100, UCDavid/NIH NeuroMab facility, N103/39).

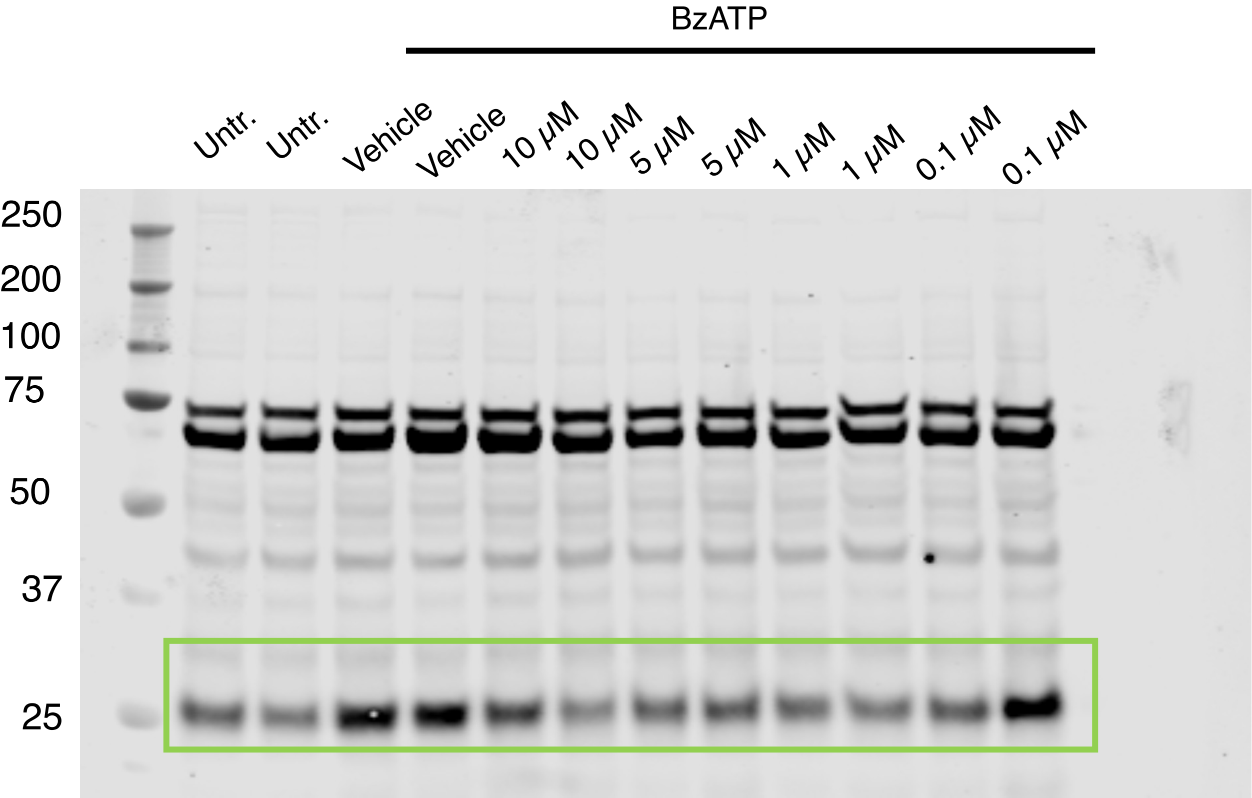

Anti-Lcn2 (in green)
(2n, anti-Gt 800, Li-Cor)

This membrane was previously incubated with antibodies against phosphorylated NFkB and total NFkB, hence the bands around 68 kDa

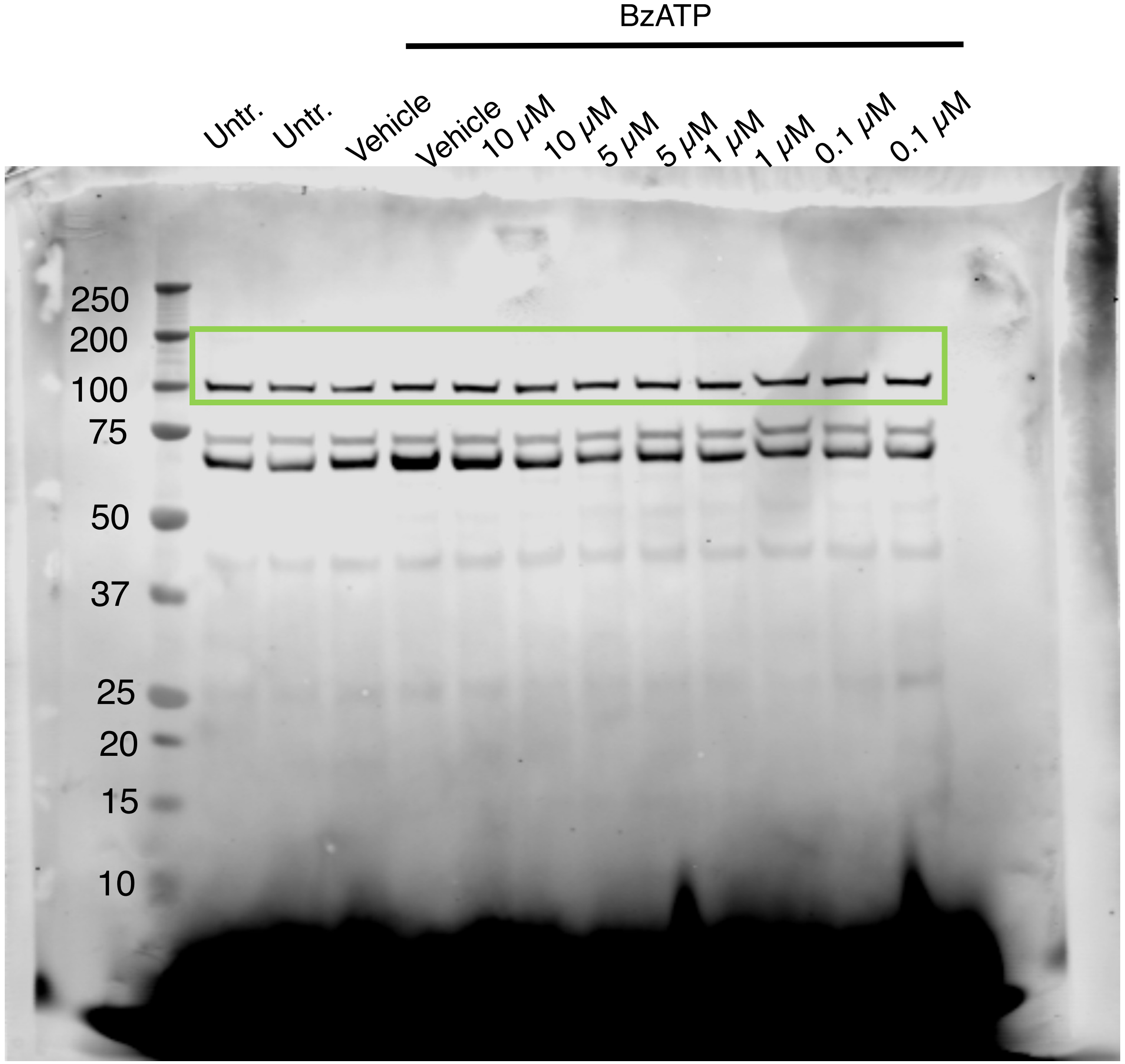

Anti-Aldh1L1 (in green)
(2n, anti-Ms 680, Li-Cor)
This membrane was previously incubated with antibodies against phosphorylated NFkB and total NFkB, hence the bands around 68 kDa

Fig. 4f Astrocyte lysates immunoblotted with antibodies phosphorylated NFkB p65 (1:1000, Cell Signalling, 3033) and total NFkB p65 (1:1000, Cell signalling, 6956). Purple rectangles indicate cell treatments that were not included in this study (e.g. treatment with Aß oligomeric species) whereas green rectangles indicate the parts of the membrane that are part of the main figure.

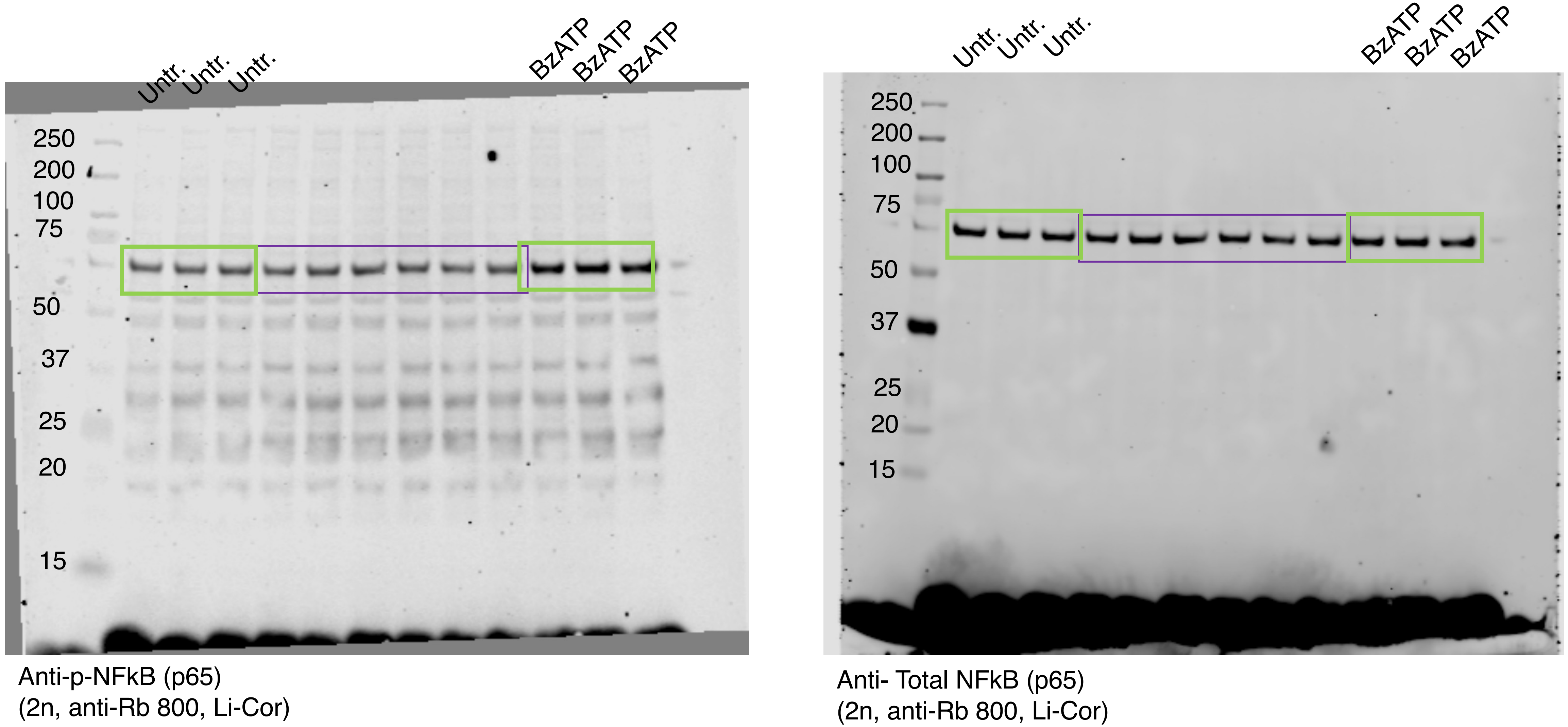

Fig. 4g Astrocyte lysates immunoblotted with antibodies phosphorylated NFkB p65 (1:1000, Cell Signalling, 3033) and total NFkB p65 (1:1000, Cell signalling, 6956). Purple rectangles indicate a condition that was quantified but not included in the main figure.

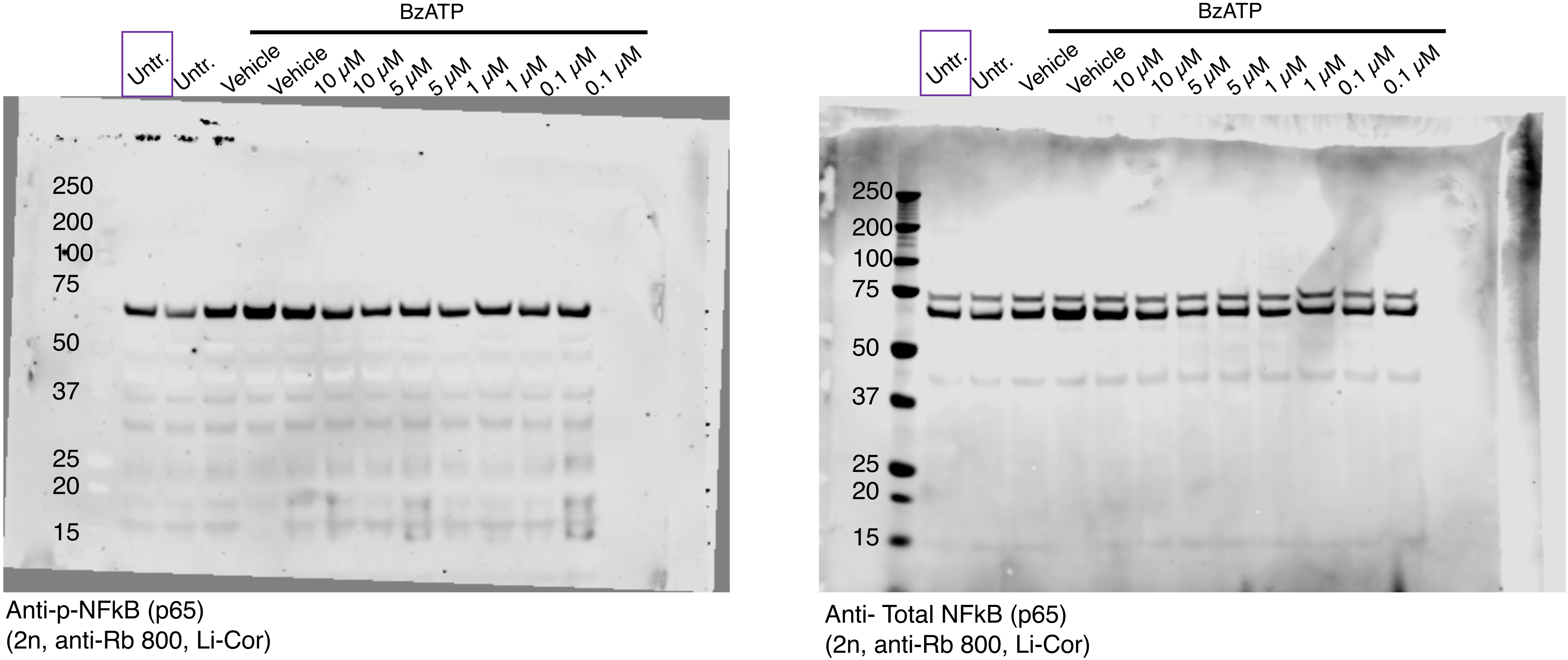

Fig. 5**c-d** Organotypic slice culture homogenates immunoblotted with antibodies against 1) PHF1 (Ms, Peter Davies, 1:1000), 2) total tau (DAKO, A0034, 1:10000), and 3) ß-actin (1:5000, abcam, Ab8226) in this order. ß-actin is indicated with a green rectangle.

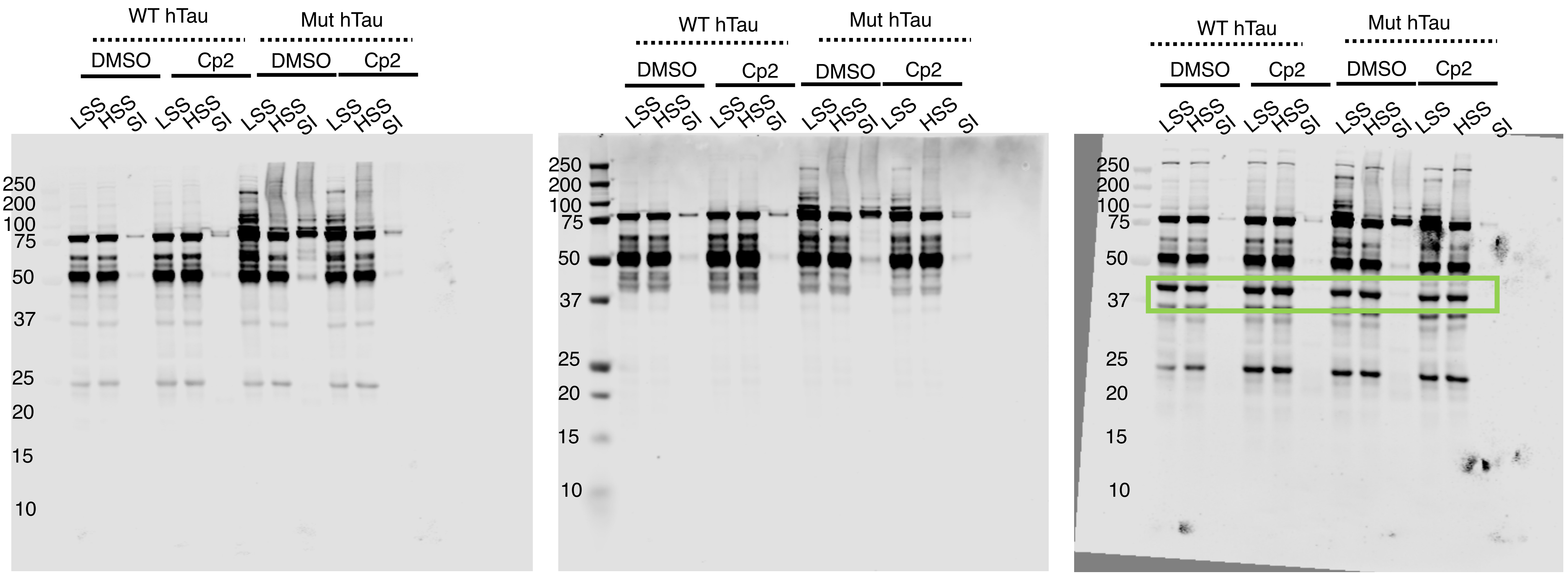

Anti-PHF1
(2n, anti-Ms 800, Li-Cor)

Anti-total tau
(2n, anti-Rb 680, Li-Cor)

Anti- ß-actin
(2n, anti-Ms 800, Li-Cor)

Fig. 5**e-f** Organotypic slice culture homogenates immunoblotted with antibodies against 1) PHF1 (Ms, Peter Davies, 1:1000), 2) total tau (DAKO, A0034, 1:10000), and 3) PSD-95 (Millipore, MAB1596, 1:1000) in this order. PSD-95 is indicated with a green rectangle.

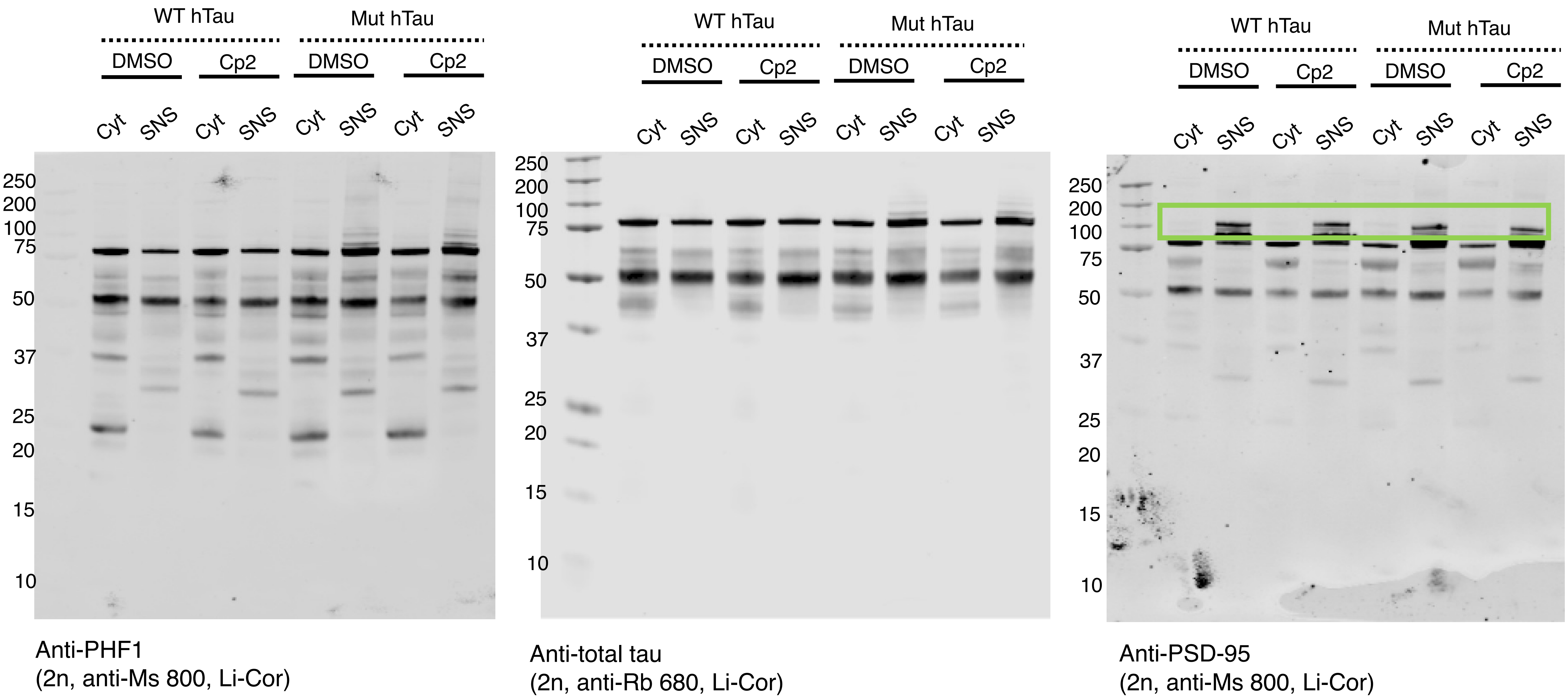

Fig. S1c HEK293 lysates of cells transiently expressing two clones of $P2X_7R$ (C1, C2) or nontransfected immunoblotted with anti-$P2X_7R$ antibody (Novus Biologicals, NBP1-37775, 1:200), FLAG (Sigma-Aldrich, F3165, 1:1000), ß-actin (1:5000, Abcam, Ab8226). Membranes were cut before incubation with primary antibodies.

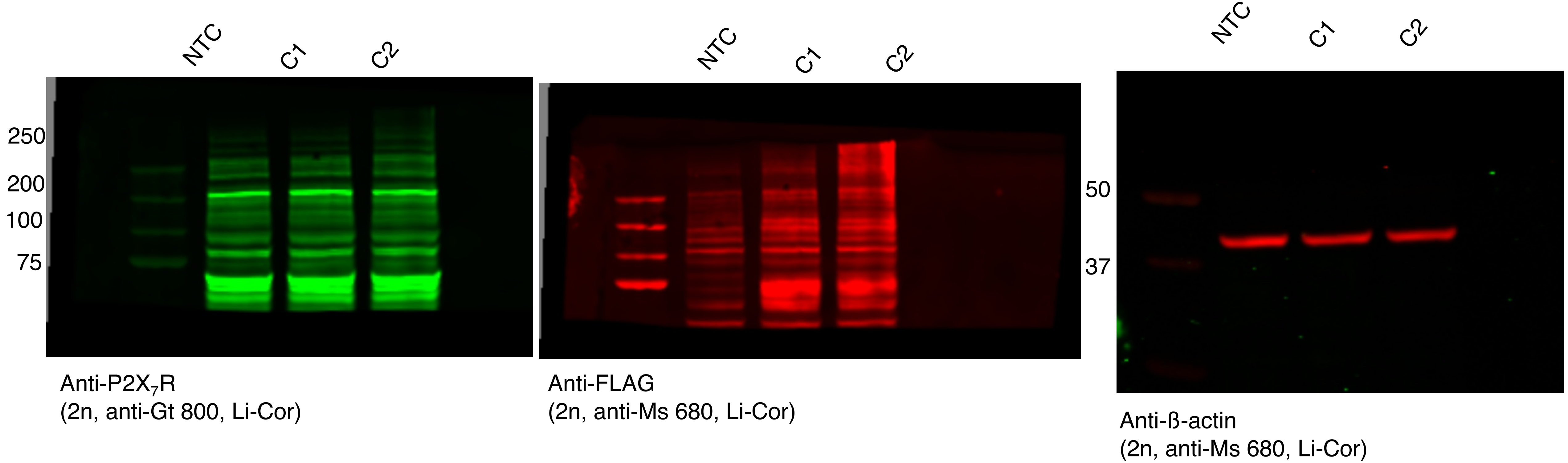

Fig. S1d HEK293 lysates of cells transiently expressing two clones of $P2X_7R$ (C1, C2) or nontransfected immunoblotted with anti-$P2X_7R$ antibody (Thermo-Fischer, PA5-29274, 1:100), FLAG (Sigma-Aldrich, F3165, 1:1000), ß-actin (1:5000, Abcam, Ab8226). Membranes were cut before incubation with primary antibodies.

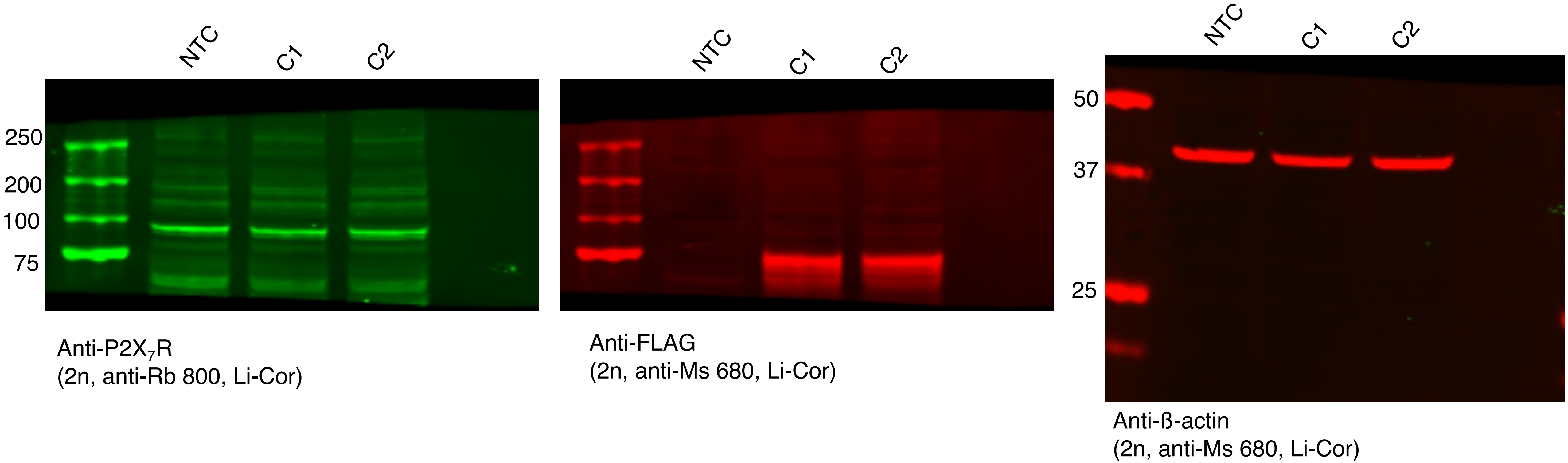

Fig. S1e. HEK293 lysates of cells transiently expressing two clones of P2X$_7$R (C1, C2) or nontransfected immunoblotted with anti-P2X$_7$R antibody (Alomone, APR-004, 1:200), FLAG (Sigma-Aldrich, F3165, 1:1000), ß-actin (1:5000, Abcam, Ab8226). Membranes were cut before incubation with primary antibodies.

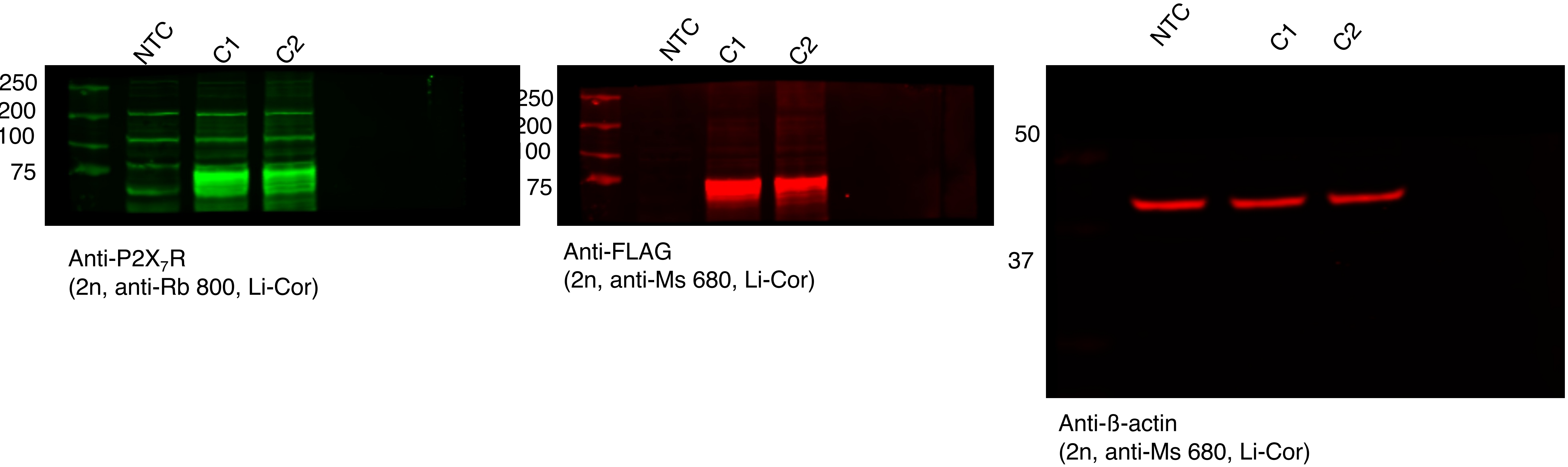

Fig. S1f HEK293 lysates of cells transiently expressing two clones of $P2X_7R$ (C1, C2) or nontransfected immunoblotted with anti-$P2X_7R$ antibody (Proteintech, 28207-1-AP, 1:100), FLAG (Sigma-Aldrich, F3165, 1:1000), ß-actin (1:5000, Abcam, Ab8226). Membranes were cut before incubation with primary antibodies.

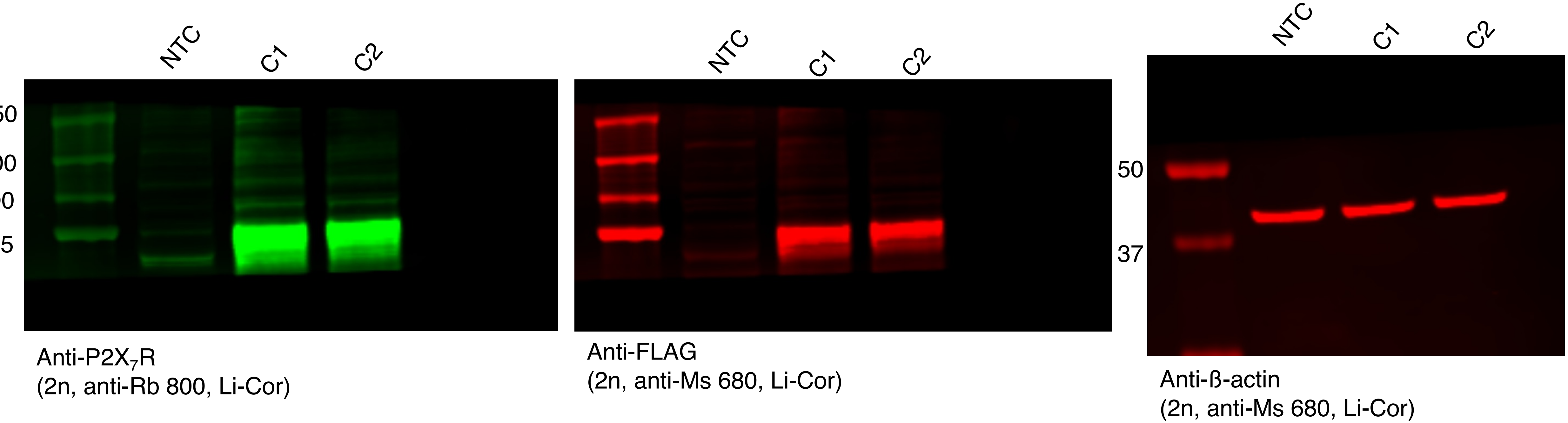

Fig. S2a BA9 total brain homogenates immunoblotted with antibodies against total tau (DAKO, A0034, 1:10000) and NSE (DAKO, M0873). The purple rectangle indicates a severe AD case remove from the study (not quantified) because it did not match the neuropathological requirements (low Aß, and phosphorylated tau).

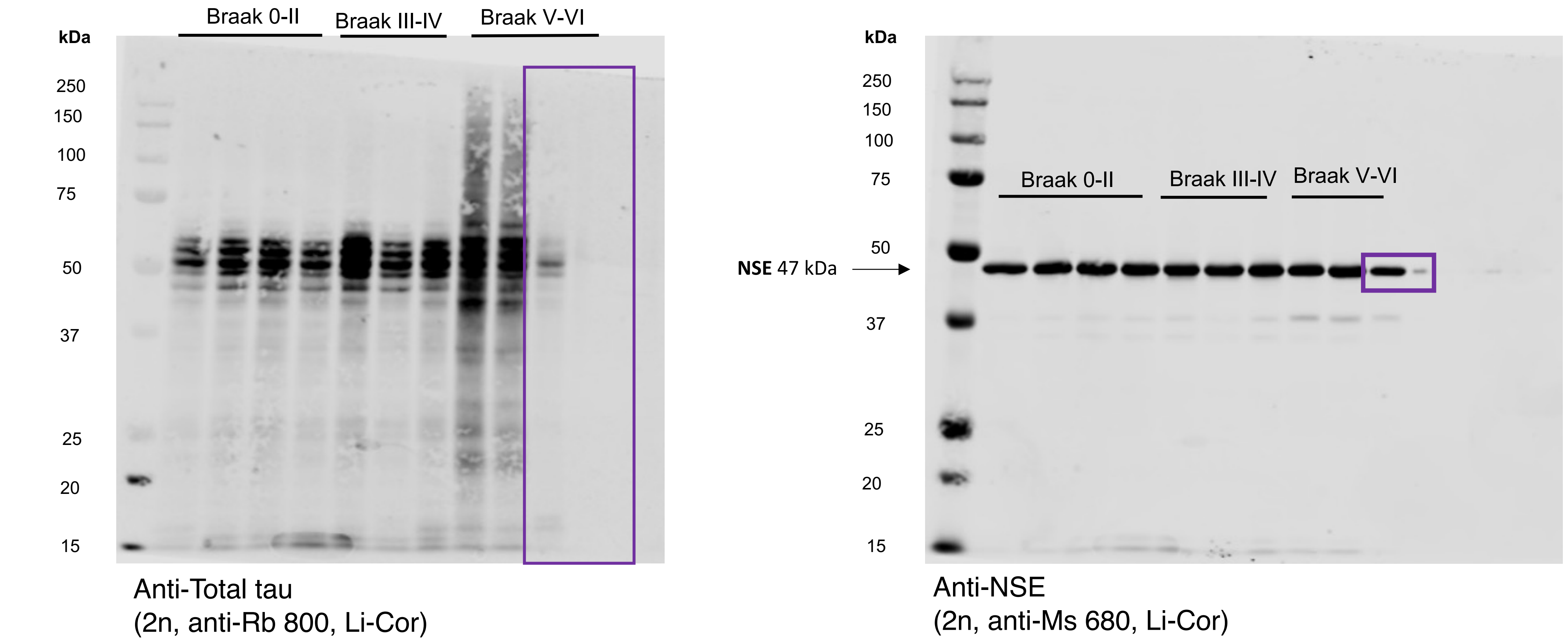

Fig. S2b BA9 total brain homogenates immunoblotted with antibodies against total tau (DAKO, A0034, 1:10000) and PHF1 (Peter Davies, 1:1000). The purple square indicates a severe AD case remove from the study (not quantified) because it did not match the neuropathological requirements (low Aß, and phosphorylated tau).

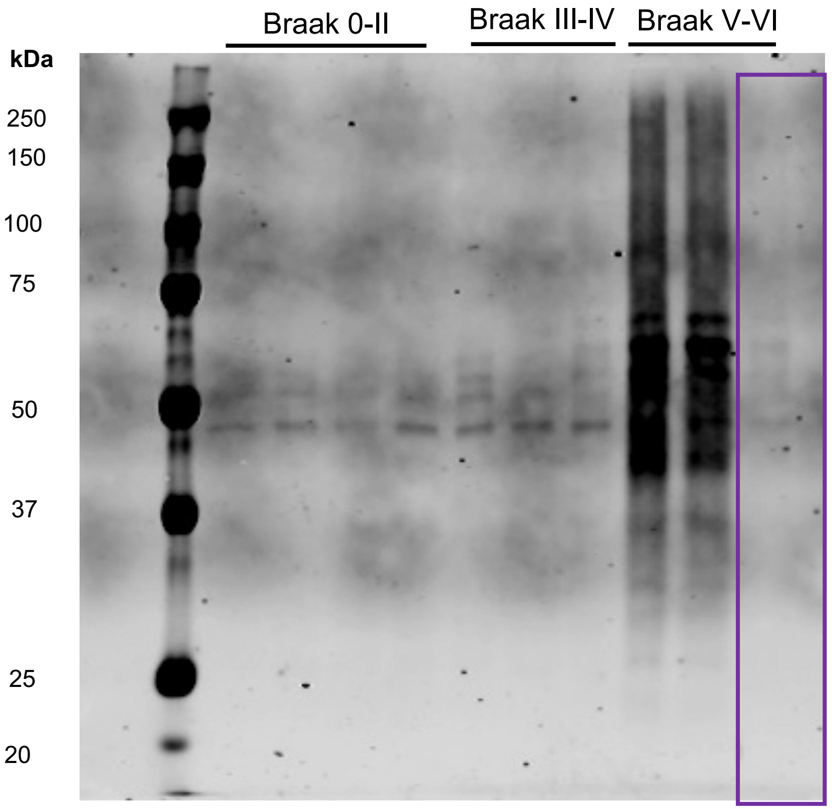

Anti-PHF1
(2n, anti-Ms 800, Li-Cor)

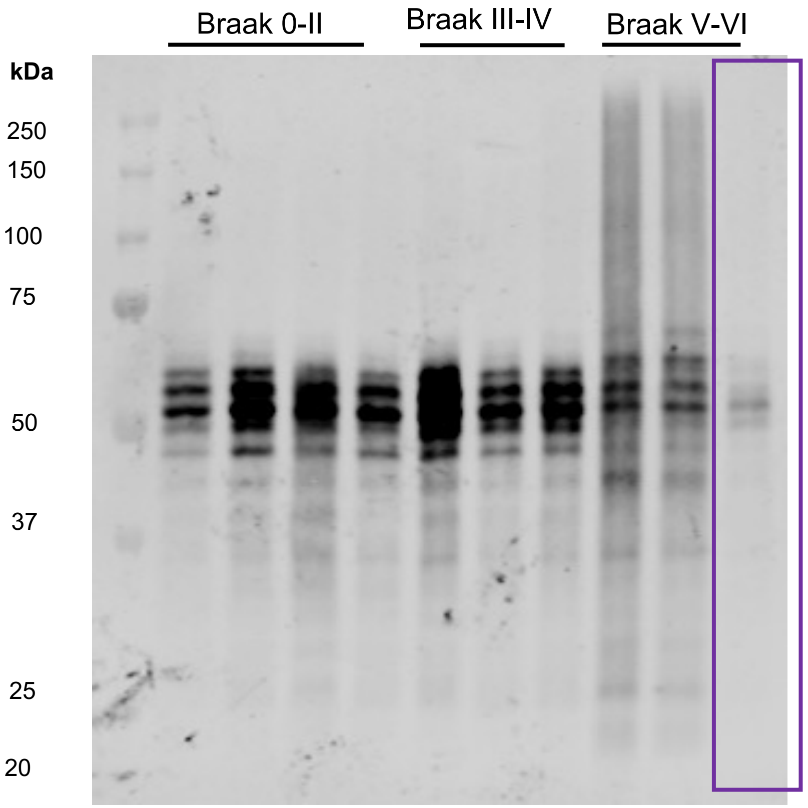

Anti-Total tau
(2n, anti-Rb 680, Li-Cor)

Fig. S2c-d SNS and cytosolic fractions from BA9 AD and control brain immunoblotted with antibodies against total tau (DAKO, A0034, 1:10000) and PHF1 (Peter Davies, 1:1000). The green rectangle the part of the blot used as a main figure.

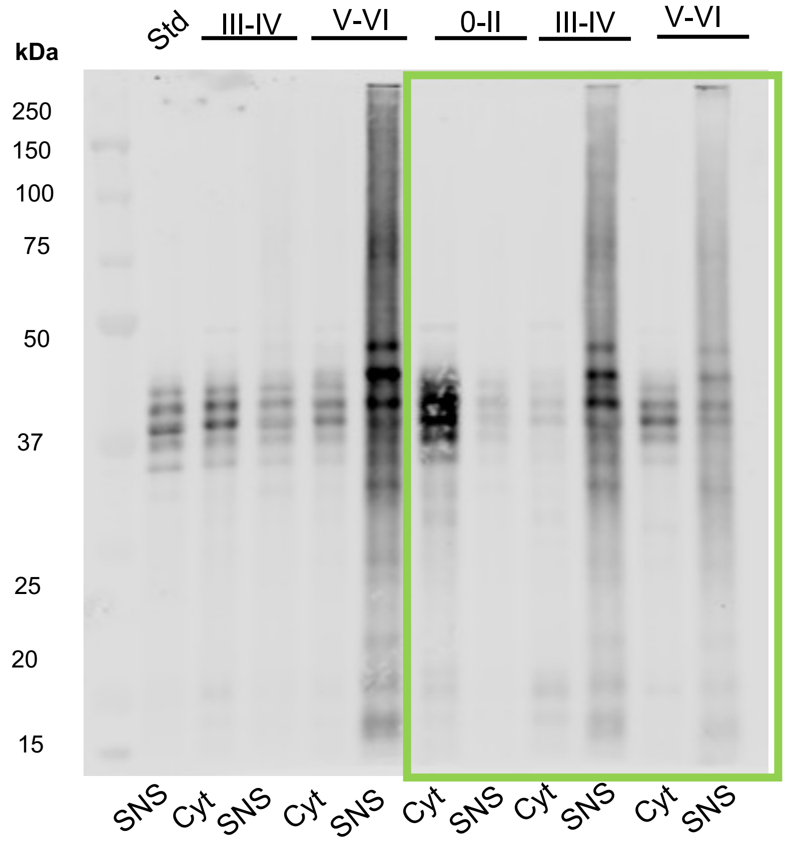

Anti-Total tau
(2n, anti-Rb 680, Li-Cor)

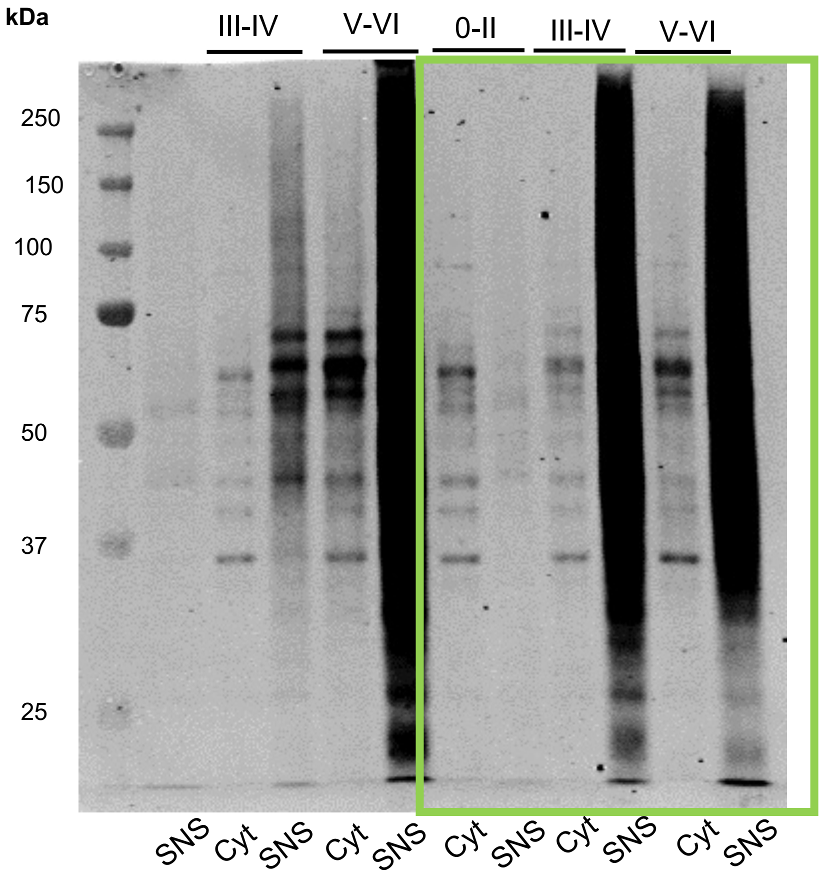

Anti-PHF1
(2n, anti-MS 800, Li-Cor)

Fig. S2e SNS fraction from BA9 AD and control brain immunoblotted with antibodies against Aß clone 6E10 (BioLegend, 803001, 1:200) and ß-actin (1:5000, Abcam). The purple rectangle indicates a severe AD case remove from the study (not quantified) because it did not match the neuropathological requirements (low Aß, and phosphorylated tau). The green square represents the part of the membrane included in the main figure.

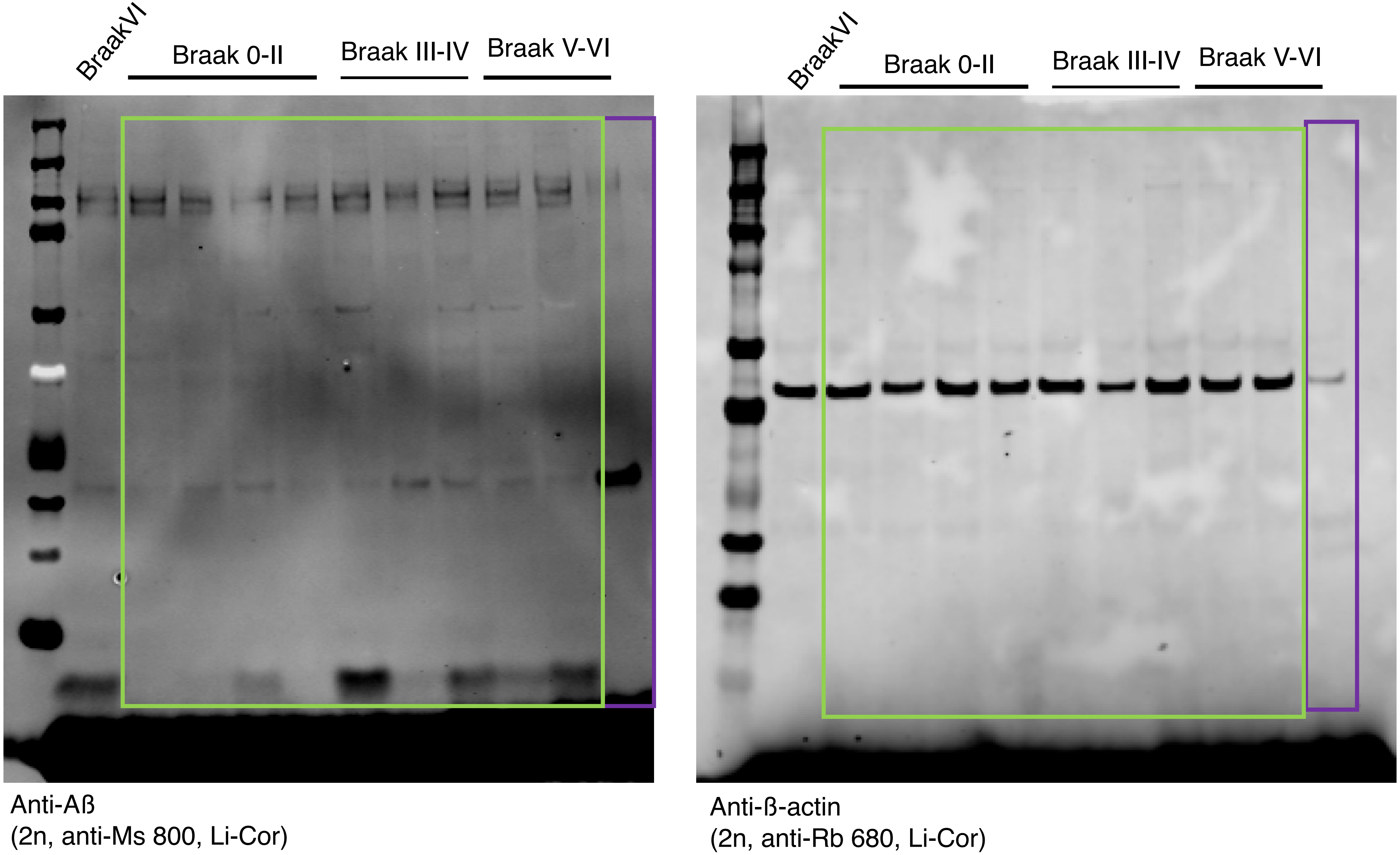

Fig. S4b Mouse Astrocytic, microglial (and neuron) lysates immunoblotted with an antibody against $P2X_7R$ (1:200, Alomone, APR-004) and ß-actin (1:5000, Abcam, Ab8226). The membrane was cut prior to incubation with primary antibodies. The green rectangle indicates the part of the blot used in the main figure.

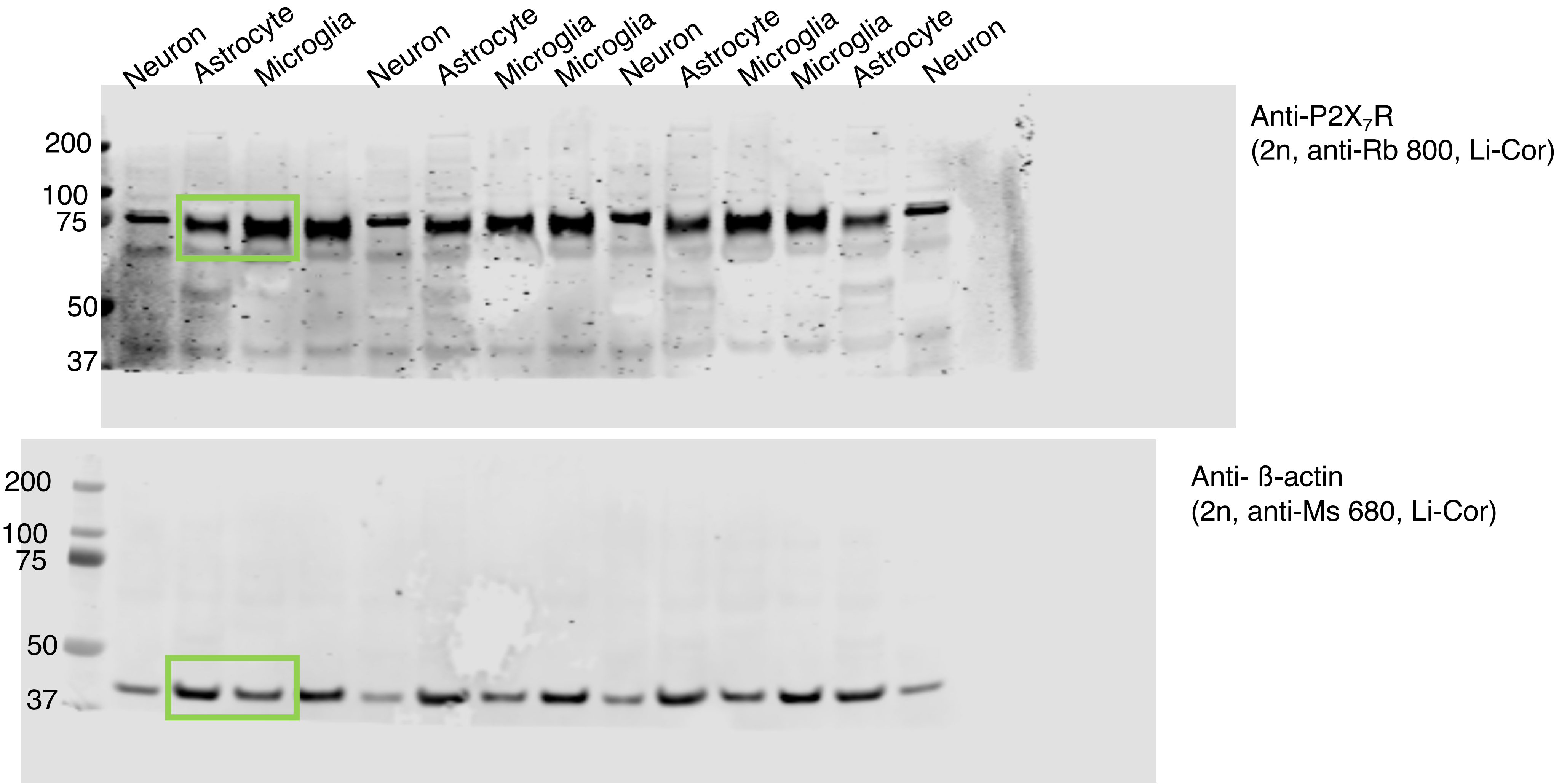

Fig. S4d Mouse microglial lysates immunoblotted with an antibody against NLRP3 (1:1000, Adipogen, AG-20B-0014-C100) and ß-actin (1:5000, Abcam, Ab8226). The green rectangle indicates the part of the blot used in the figure.

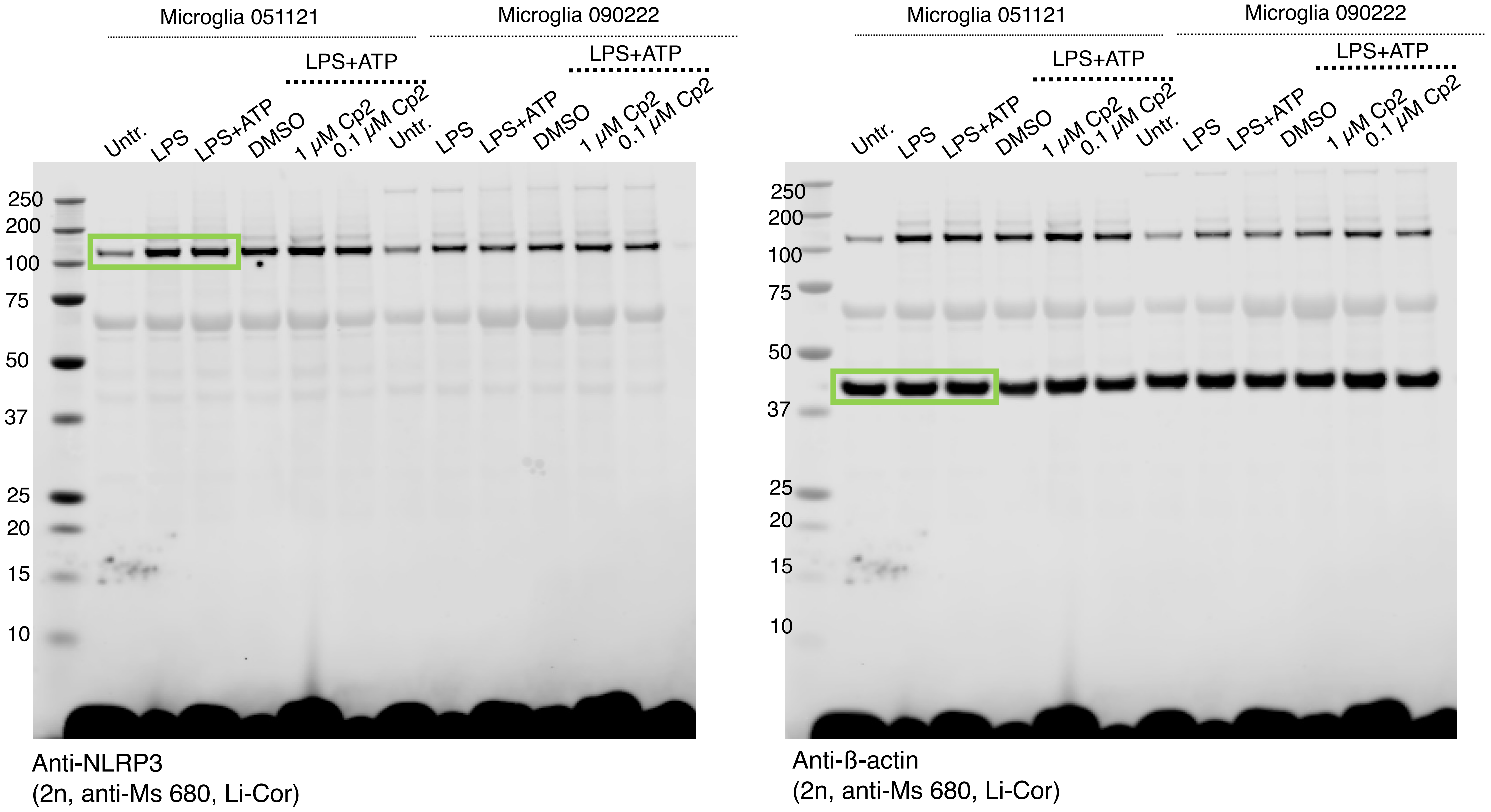

Fig. S4i Mouse astrocyte and microglial lysates immunoblotted with an antibody against NLRP3 (1:1000, Adipogen, AG-20B-0014-C100), ASC (1:1000, Adipogen, AG-25B-0006-C100) and ß-actin (1:5000, Abcam, Ab8226) in this order. The green rectangle indicates the part of the blot used in the figure.

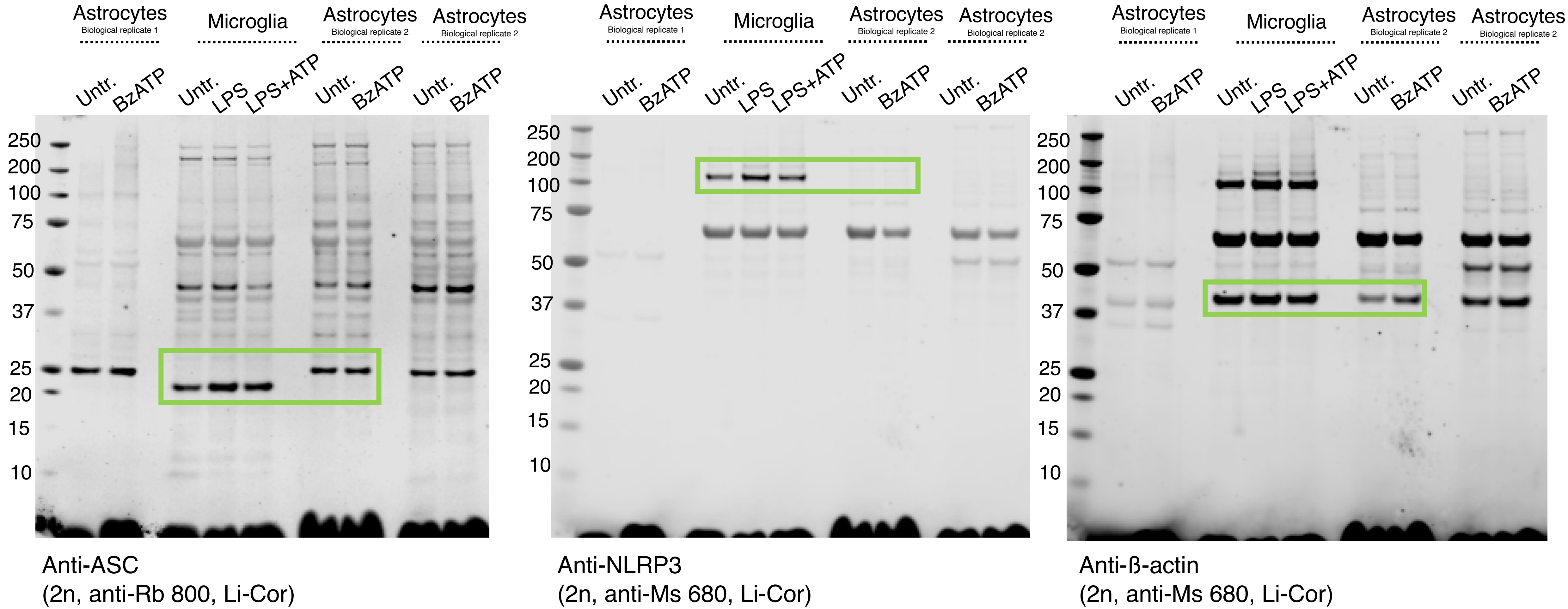

Fig. S4l Mouse microglial lysate immunoblotted with antibodies against phosphorylated NFkB p65 (1:1000, Cell Signalling, 3033) and total NFkB p65 (1:1000, Cell Signalling, 6956). Purple rectangles indicate cell treatments that were not included in this study (e.g. treatment with Aß oligomeric species) whereas green rectangles indicate the part of the membrane used for the main figure.

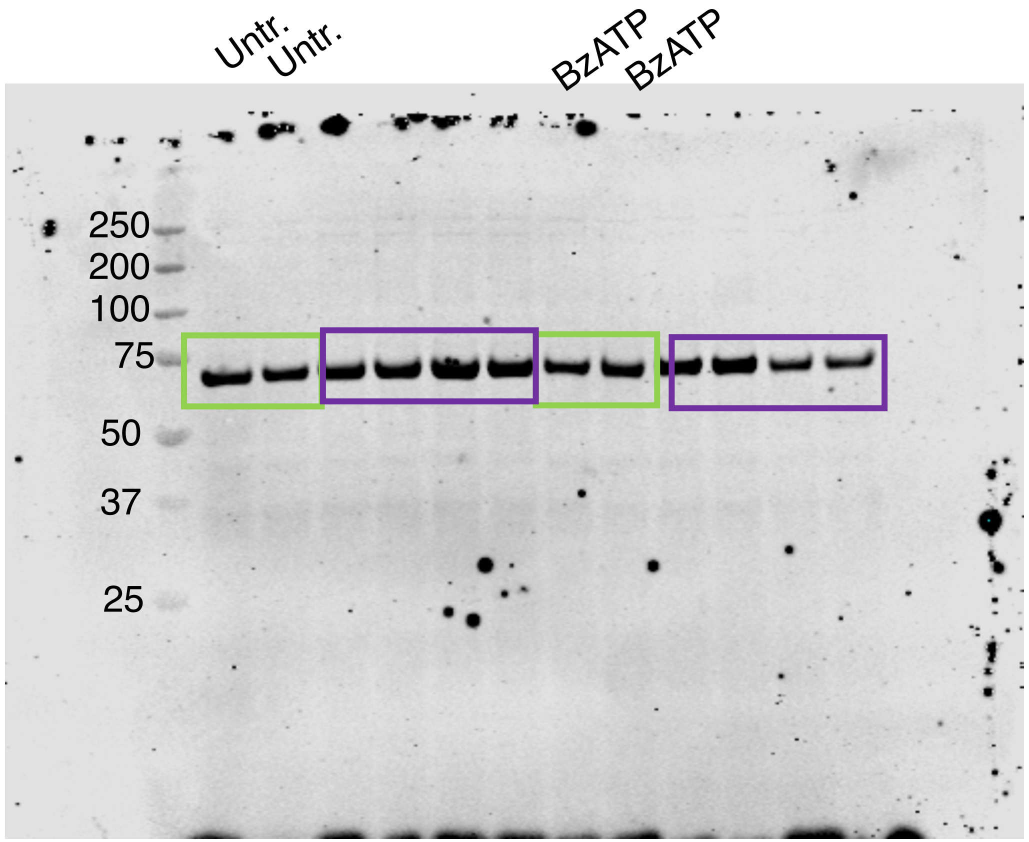

Anti-p-NFkB (p65)
(2n, anti-Rb 800, Li-Cor)

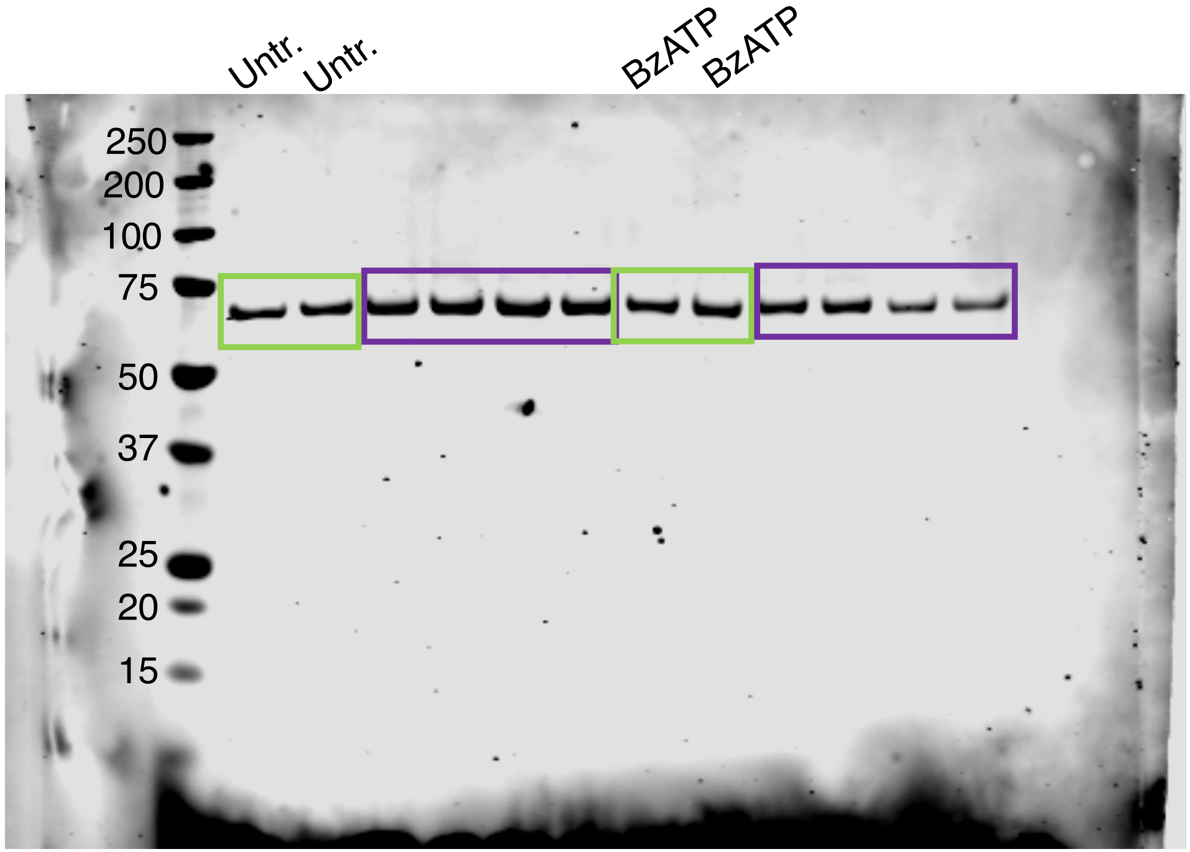

Anti- total NFkB (p65)
(2n, anti-Ms 680, Li-Cor)

Fig. S5a-b Organotypic slice culture homogenates immunoblotted with antibodies against 1) PHF1 (Ms, Peter Davies, 1:1000), 2) total tau (DAKO, A0034, 1:10000) in this order.

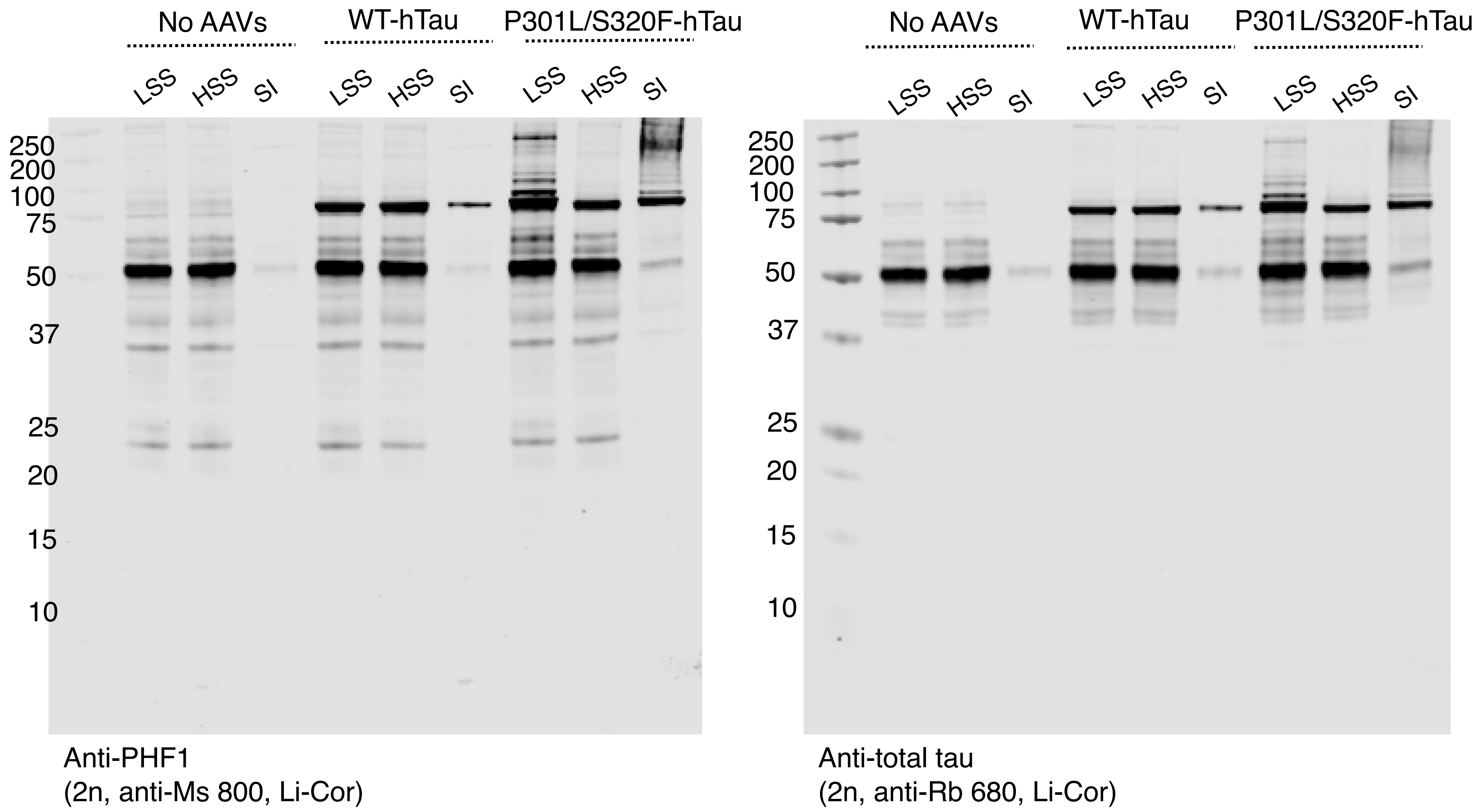

Fig. S6 Human BA9 AD brain sarkosyl-extraction homogenates immunoblotted with antibodies against 1) PHF1 (Ms, Peter Davies, 1:1000), 2) total tau (DAKO, A0034, 1:10000), and 3) in this order. Standard (Std) is a total homogenate of a severe (Braak VI) case used in every blot for normalisation. Green rectangles indicate the part of the membrane used for the main figure.

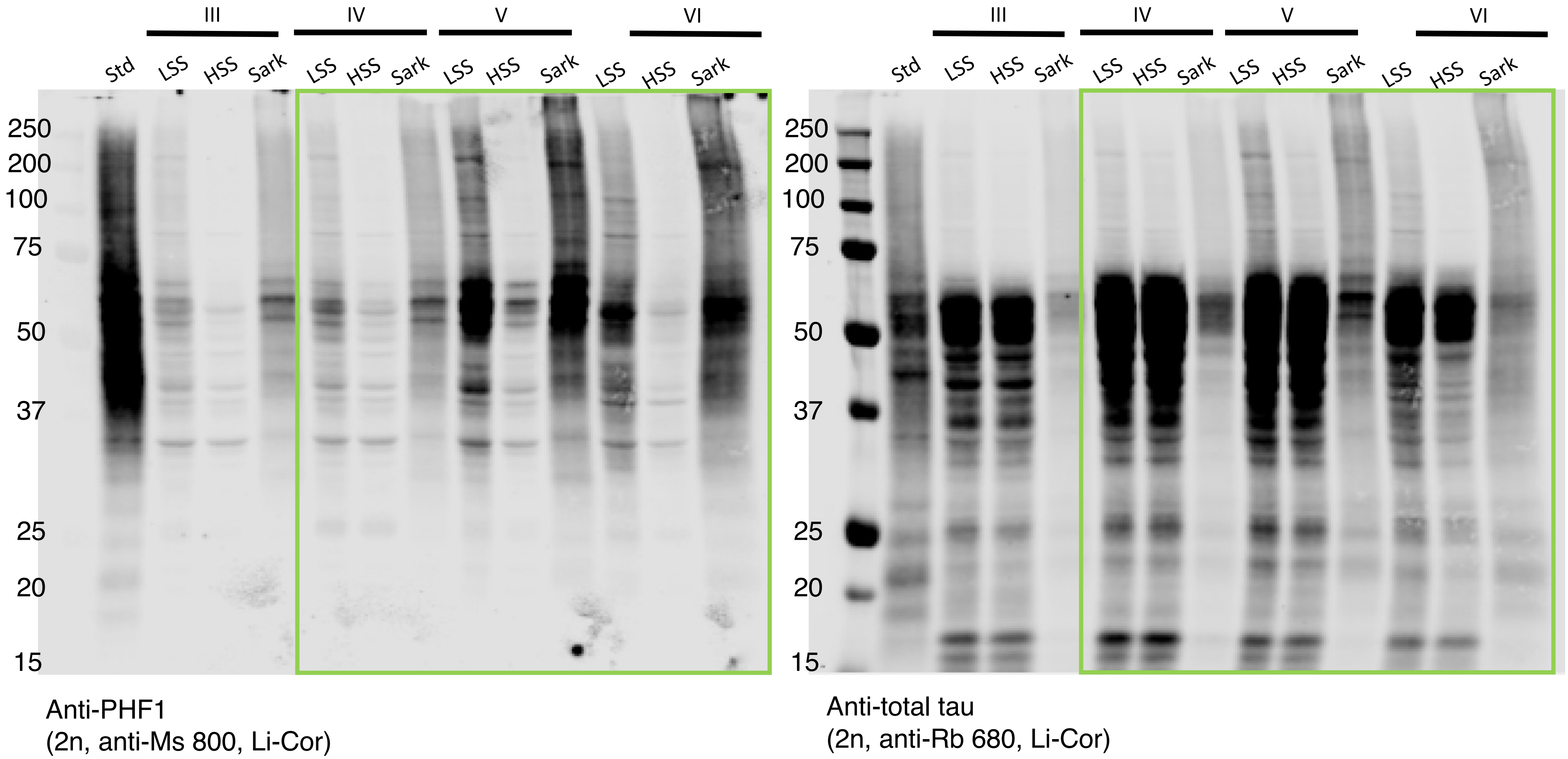

Supplement: Supplementary data 3 [file mmc3.pdf]
